# Interplay between β-Diimino and β-Diketiminato Ligands in Nickel Complexes Active in the Proton Reduction Reaction

**DOI:** 10.1021/acs.inorgchem.2c02150

**Published:** 2022-10-05

**Authors:** Navid Jameei Moghaddam, Marcos Gil-Sepulcre, Jia-Wei Wang, Jordi Benet-Buchholz, Carolina Gimbert-Suriñach, Antoni Llobet

**Affiliations:** † Institute of Chemical Research of Catalonia (ICIQ), Barcelona Institute of Science and Technology (BIST), Avda. Països Catalans 16, 43007Tarragona, Spain; ‡ Departament de Química Física i Inorgànica, Universitat Rovira i Virgili, Marcel·lí Domingo s/n, 43007Tarragona, Spain; § Departament de Química, Universitat Autònoma de Barcelona, Cerdanyola del Vallès, 08193Barcelona, Spain

## Abstract

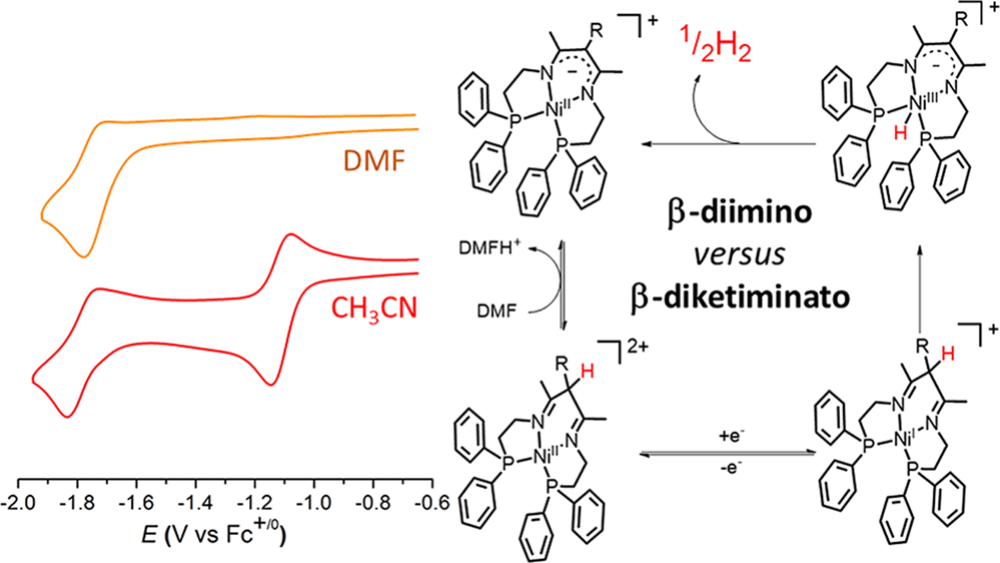

Two Ni complexes are reported with κ^4^-P_2_N_2_ β-diimino (BDI) ligands with the general formula [Ni(XBDI)](BF_4_)_2_, where BDI is *N*-(2-(diphenylphosphaneyl)ethyl)-4-((2-(diphenylphosphaneyl)ethyl)imino)pent-2-en-2-amine and X indicates the substituent in the α-carbon intradiimine position, X = H for **1**(BF_4_)_2_ and X = Ph for **2**(BF_4_)_2_. Electrochemical analysis together with UV–vis and NMR spectroscopy in acetonitrile and dimethylformamide (DMF) indicates the conversion of the β-diimino complexes **1^2+^** and **2^2+^** to the negatively charged β-diketiminato (BDK) analogues (**1-H**)^+^ and (**2-H**)^+^ via deprotonation in DMF. Moreover, further electrochemical and spectroscopy evidence indicates that the one-electron-reduced derivatives **1^+^** and **2^+^** can also rapidly evolve to the BDK (**1-H**)^+^ and (**2-H**)^+^, respectively, via hydrogen gas evolution through a bimolecular homolytic pathway. Finally, both complexes are demonstrated to be active for the proton reduction reaction in DMF at *E*_app_ = −1.8 V vs Fc^+/0^, being the active species the one-electron-reduced derivative **1-H** and **2-H**.

## Abstract 2

The conversion of a β-diimino Ni(II) complex to the corresponding β-diketiminato Ni(II) derivative upon one-electron reduction and hydrogen gas formation is demonstrated, highlighting the potential role of these types of ligands as proton relays in catalytic processes such as the proton reduction to hydrogen transformation.

## Introduction

β-Diketiminates (often abbreviated as *nacnac* or BDK) are a family of ligands that have been widely used for synthesizing a large amount of complexes with different metal centers, all across the periodic table.^1,2^ Since their introduction in 1968,^3−6^ BDK ligands have gained lots of interest because of their versatility and tunability as well as their strong binding to metal centers. The variation of the ligand’s steric and electronic properties is possible by changing the substituent on the imine N atom and the α-carbons.^7,8^ In addition, although BDK have been long considered spectator ligands, several reports show that they can also act as non-innocent ligands.^9−12^ There is a considerable diversity of bonding modes from pure σ to a combination of σ to π donation for β*-*diketiminato ligands to metal complexes, in which most of the coordination modes are metal center dependent. The most common is the κ^2^-N,N′ bidentate coordination through the two nitrogen atoms, affording a six-membered metalacyclic ring.^1,13^

**Figure 1:**
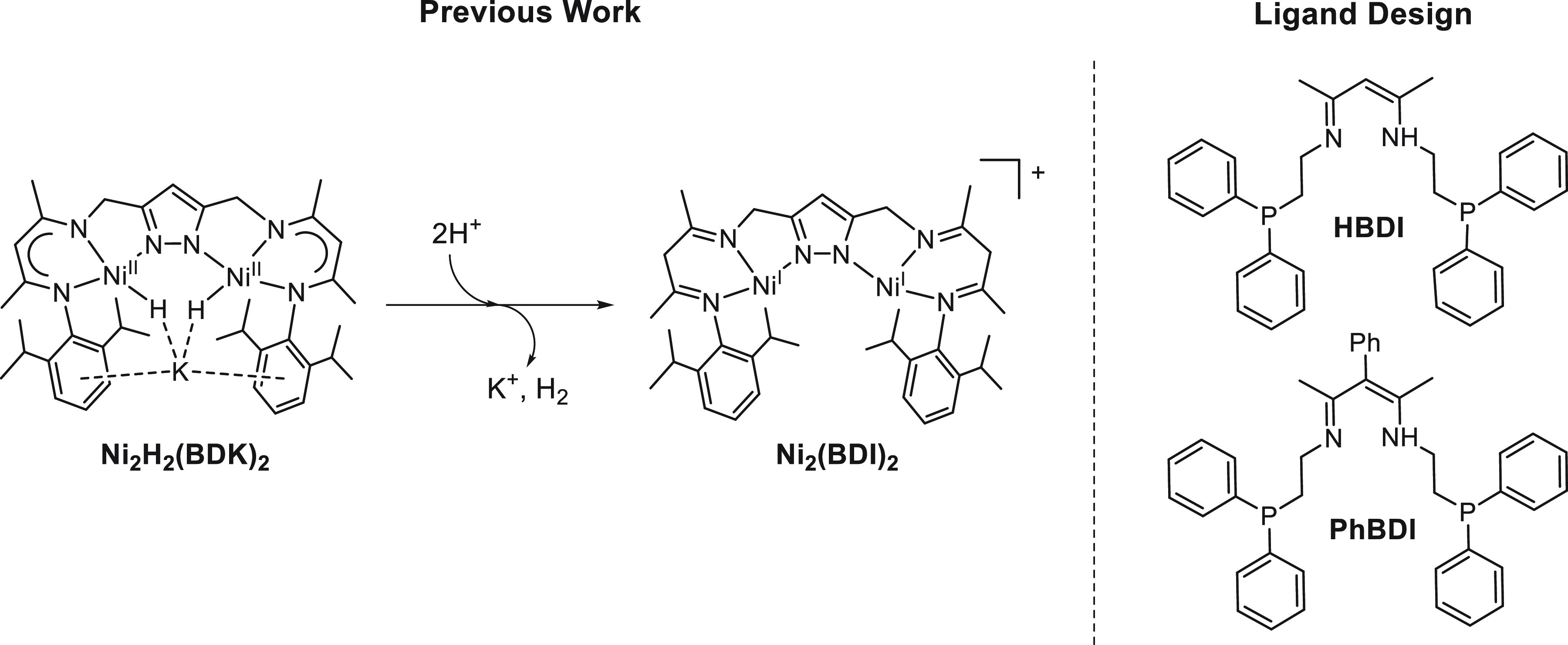
(Left) Stoichiometric H_2_ evolution from a dihydrido dinickel bis(β-diketiminato) complex reported in the literature.^22,23^ (Right) β-Diimino ligands containing two phosphine arms designed in this work as precursors to tetradentate β-diketiminato complexes.

First-row transition metal β-diketiminato complexes have been used for a wide variety of applications including stoichiometric and catalytic transformations such as functionalization of alkenes, cross-coupling reactions, and energy-related applications.^14−20^ In addition, late transition metal complexes have been used for synthesizing low coordination metal centers mimicking the active site of metalloproteins for the conversion of organic substrates but also the activation of small molecules such as carbon dioxide or dinitrogen.^14,21^ More recently, Meyer and coworkers have published a dinuclear bis(β-diketiminato) complex bridged by a pyrazolate ring, which has been shown to be involved in the stoichiometric proton reduction reaction to produce hydrogen through a proposed metal–metal and metal–ligand cooperative two-electron reductive process.^22,23^ In their work, they fully characterized the dihydride species **Ni_2_H_2_(BDK)_2_** in Figure 1 (left) with two Ni(II) centers, prepared from the corresponding bromido-bridged derivative in the presence of KHBEt_3_. The species **Ni_2_H_2_(BDK)_2_** converts to the corresponding bis(β-diimino) derivative **Ni_2_(BDI)_2_** containing two three-coordinated Ni(I) centers in the presence of an acid source, as demonstrated by a full range of spectroscopic and magnetic techniques.

In this work, we exploit the ability of BDI/BDK ligands in proton to hydrogen conversion by designing two tetradentate symmetric κ^4^-P_2_N_2_ diimino ligands containing two aliphatic diphenylphosphine arms giving enough flexibility to allow tetra coordination with required geometry (Figure 1, right). While the BDK/BDI moiety was expected to provide a non-redox-active but useful proton relay fragment, the two coordinated phosphine groups would provide extra coordination sites while allowing to stabilize metallic low oxidation states, thanks to the π-back bonding ability of phosphine ligands. Two different ligands have been designed with different groups (HBDI and PhBDI) in the α-carbon intradiimine position, tuning the acidity of the BDI proton to give the corresponding BDK derivative. Thus, we describe the synthesis, spectroscopic, and electrochemical characterization of the nickel complexes derived from HBDI and PhBDI and their conversion to the corresponding β-diketiminato derivatives in the context of the proton reduction reaction to produce hydrogen.

## Results and Discussion

### Synthesis and Structural Characterization of Complexes 1(BF_4_)_2_ and 2(BF_4_)_2_

**Scheme 1:**
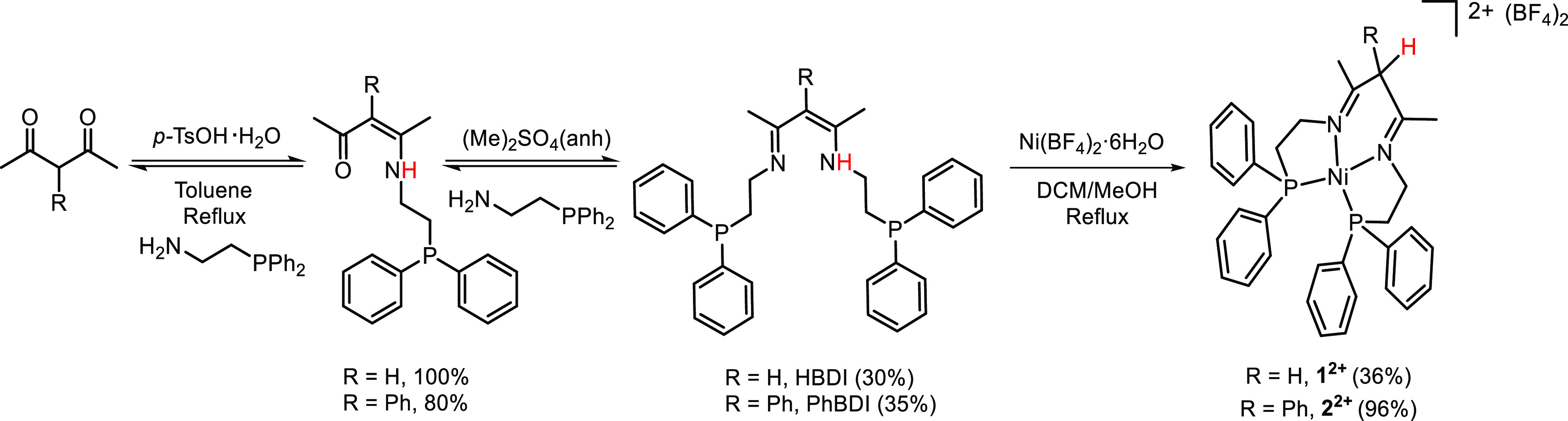
Synthesis of Ligands and Complexes Described in This Work

Symmetrical κ^4^-P_2_N_2_ ligands derived from β-diketiminate are scarce in the literature, despite the well-known capacity of both BDK and phosphine groups to stabilize coordination complexes.^24,25^ Other examples include only one phosphine arm connected to the BDK ligand.^20,26^ HBDI and PhBDI ligands in Figure 1 and Scheme 1 are two novel tetradentate ligands with two nitrogen and two phosphorous donors that differ on the substituent of the α-C position (H for HBDI and Ph for PhBDI). The synthesis of the ligands HBDI and PhBDI was performed through two consecutive condensation steps in oxygen and moisture free conditions. Briefly, the first condensation was performed by refluxing the corresponding acetylacetone and 2-(diphenylphosphaneyl)ethylamine in the presence of a catalytic amount of acid in toluene, and the second condensation was performed using dimethyl sulfate in a solvent-free reaction with yields of 30 and 35% for HBDI and PhBDI, respectively (Scheme 1). The synthesis of Ni complexes from the ligands HBDI and PhBDI is straightforward using Ni(BF_4_)_2_·6H_2_O as metal precursor, obtaining moderate to good reaction yields (36 and 96% for complexes **1(BF_4_)_2_** and **2(BF_4_)_2_**, respectively). The lower yield obtained for **1(BF_4_)_2_** is due to the difficulties in the purification of the compound, which requires a slow crystallization process in a mixture of acetonitrile/diethyl ether at −30 °C, as described in detail in the Experimental Section.

**Figure 2:**
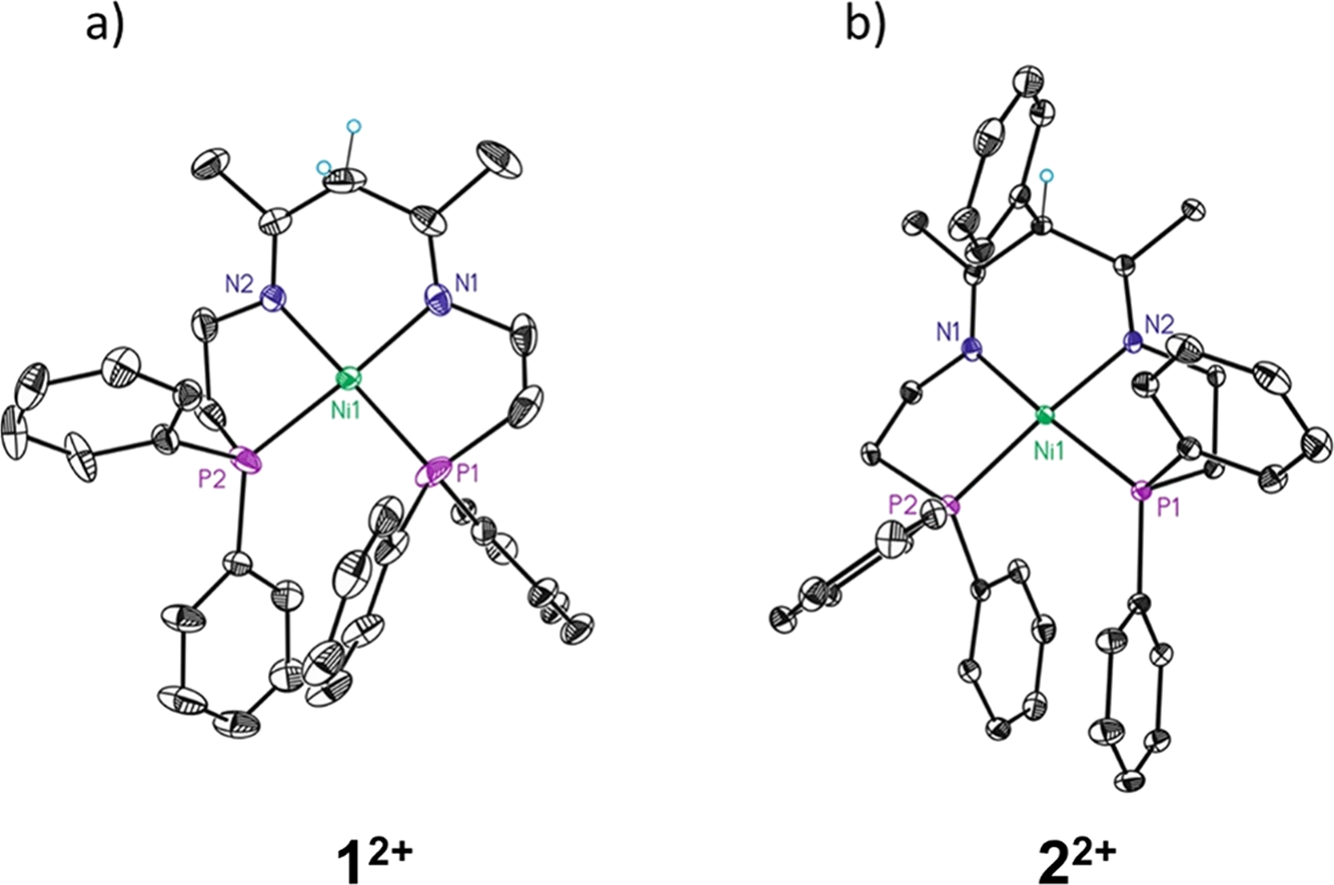
ORTEP representation of **1^2+^**(**a**) and **2^2+^**(**b**) at 50% probability level (hydrogens are omitted for further clarification, except for α-carbon hydrogen). Color code: C, dark gray; N, blue; Ni, green; P, purple.

Single crystals suitable for X-ray diffraction analysis were grown by layering Et_2_O on top of a saturated solution of complexes **1(BF_4_)_2_** or **2(BF_4_)_2_** in CH_3_CN while keeping the mixture at −30 °C. The ORTEP representations of the cationic part of both complexes, shown in Figure 2, reveal a distorted square planar geometry with the angles of 94.56° (N1-Ni-N2), 83.06° (N2-Ni-P2), 86.48° (N1-Ni-P1), and 95.85° (P1-Ni-P2) for **1^2+^** and 92.60° (N1-Ni-N2), 86.93° (N1-Ni- P1), 83.28° (N2-Ni-P2), and 96.64° (P1-Ni-P2) for **2^2+^**. The imine C=N bond lengths ranging from 1.279(8) to 1.281(1) Å are significantly shorter than the C–N bonds linking the phosphine arms, which are in the range of 1.476(4)–1.495(3) Å. These results are in agreement with localized imine double bonds within a nonplanar ligand core with the sp^3^-hybridized α-carbon lying above the plane of the backbone, giving rise to a boat conformation.^27^

**Figure 3:**
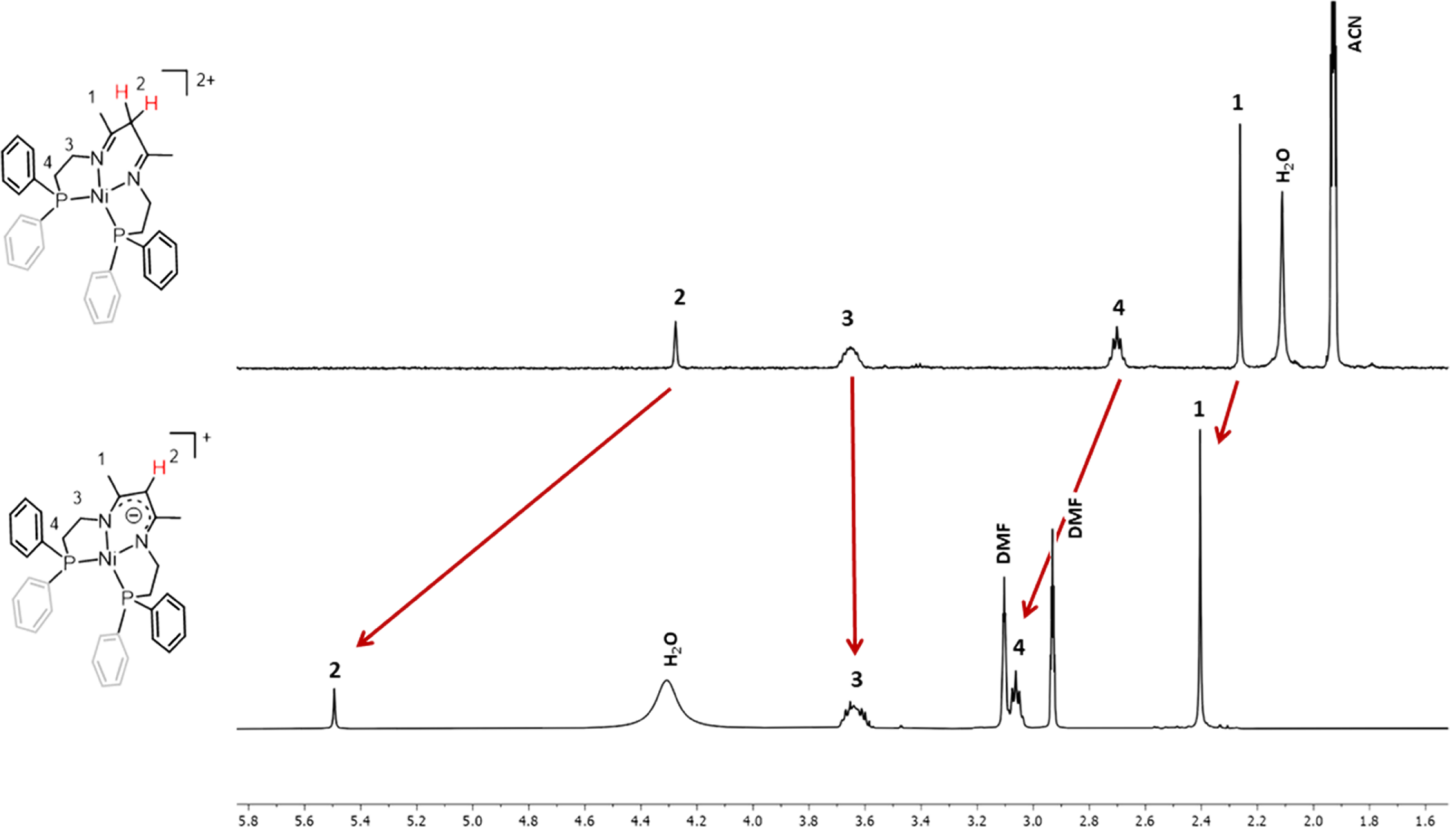
Comparison of the ^1^H NMR spectra of complex **1(BF_4_)_2_** in CD_3_CN (top) and DMF-d_7_ (bottom).

Compounds **1(BF_4_)_2_** and **2(BF_4_)_2_** are Ni(II) d^8^ diamagnetic complexes and could be fully characterized by NMR spectroscopy (Figure 3 and Figures S1–S26 in the Supporting Information). The ^1^H NMR spectra in CD_3_CN show the characteristic resonances of the α-imino protons of the sp^3^ carbon at 4.29 ppm integrating 2H for **1(BF_4_)_2_** and at 5.50 ppm integrating 1H for **2(BF_4_)_2_**. A single set of resonances per methylene group of the ligand scaffold is indicative of a symmetric structure of the complexes. This is confirmed by the ^31^P{H} NMR spectra in CD_3_CN, which show a unique singlet resonance for the two phosphine ligands at 49.64 and 47.15 ppm for **1(BF_4_)_2_** and **2(BF_4_)_2_**, respectively.

**Figure 4:**
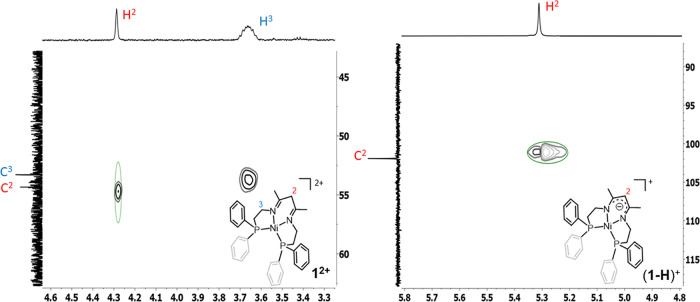
Zoom of the HSQC spectra of **1(BF_4_)_2_** in CD_3_CN (left) and DMF-d_7_ (right).

All the resonances corresponding to complex **1(BF_4_)_2_** are significantly shifted when the NMR analysis is performed in deuterated dimethylformamide (DMF-d_7_) (Figure 3 and Figures S11 and S23 in the SI). In particular, the α-imino proton appears at 5.31 ppm, accounting for a 1.02 ppm downfield shift compared to the resonance in CD_3_CN. In addition, it integrates for one proton instead of two, suggesting a deprotonation of the neutral β-diimino ligand to form a negatively charged β-diketiminato derivative (Figure 3). This is confirmed by the ^13^C NMR spectrum, where the chemical shift of the C=N imino carbon is upfield shifted from 179.97 ppm in CD_3_CN to 161.81 ppm in DMF-d_7_ and the α-carbon is downfield shifted from 53.71 ppm in CD_3_CN to 102.16 ppm in DMF-d_7_. The assignment of the α-carbon is confirmed by the clear crossed signals in the HSQC spectra (see Figure 4 and Figures S5 and S15 in the SI). This impressive shift can only be explained by the changes in hybridization and formal charge of the carbon atoms going from a β-diimino to a β-diketiminate ligand. A similar phenomenon is observed for complex **2(BF_4_)_2_** where the resonance corresponding to the α-CH proton disappears (Figure S25). Likewise, the C=N carbon shifts from 181.52 ppm in CD_3_CN to 144.81 ppm in DMF-d_7_ and the α-carbon from 67.97 ppm in CD_3_CN to 112.96 ppm in DMF-d_7_ (Figure S26). ^2^D-NMR experiments of both complexes in DMF-d_7_ rule out the possible α-imino proton exchange with the solvent and support the deprotonation of the β-diimino ligand (Figure S27). Indeed, the acidity of the α-CH proton is rather high, and it is increased once coordinated to the metal center, so that even very weak bases such as DMF induce the deprotonation of the ligand.^2,28^

**Figure 5:**
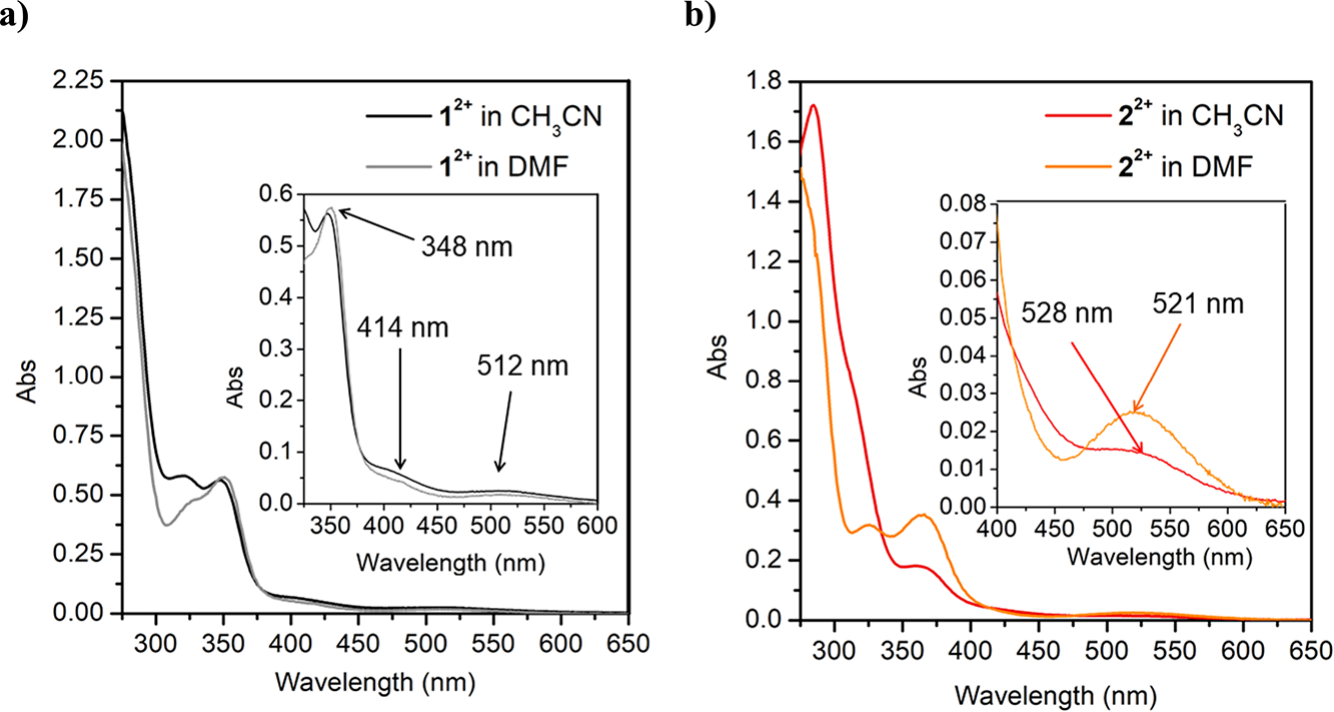
UV–vis spectra of 0.125 mM of (a) **1(BF_4_)_2_** in CH_3_CN (black) and DMF (gray) and (b) **2(BF_4_)_2_** in CH_3_CN (red) and DMF (orange).

The β-diimino compounds **1(BF_4_)_2_** and **2(BF_4_)_2_** and the β-diketiminato derivatives (hereafter (**1-H**)**BF_4_** and (**2-H**)**BF_4_**) were also characterized by UV–vis spectroscopy (Figure 5; see also Figures S38 and S39). Complex **1(BF_4_)_2_** in acetonitrile shows an intense absorption below 300 nm and two well-defined absorption bands at 323 nm (ε = 3720 M^–1^ × cm^–1^) and 348 nm (ε = 4528 M^–1^ × cm^–1^) with a shoulder at 414 nm (ε = 460.72 M^–1^ × cm^–1^) (black trace in Figure 5a). These bands are attributed to intraligand π–π* transitions. Finally, a very weak absorption band at 512 nm (ε = 196.56 M^–1^ × cm^–1^) appears that is assigned to the d–d transition of the low-spin square planar Ni(II) complex.^29^

As discussed above, dissolving **1(BF_4_)_2_** in DMF affords the β-diketiminato derivative, which shows a very similar UV–vis spectrum (gray trace in Figure 5a) with exactly the same bands but less intense at 323 nm (ε = 3050 M^–1^ × cm^–1^), a shoulder at 414 nm (ε = 356.56 M^–1^ × cm^–1^), and 512 nm (ε = 134.24 M^–1^ × cm^–1^). In contrast, the band at 348 nm (ε = 4578 M^–1^ × cm^–1^) is slightly more intense (Figure 5a, inset). The differences in the absorption spectra of **2(BF_4_)_2_** and (**2-H**)**BF_4_** are more pronounced, although the band positions are also very similar (Figure 5b, red and orange traces, respectively). The spectrum in CH_3_CN shows bands at 284 nm (ε = 13770 M^–1^ × cm^–1^), a shoulder at 313 nm (ε = 6499 M^–1^ × cm^–1^), and two other bands at 365 nm (ε = 1507 M^–1^ × cm^–1^) and 528 nm (ε = 112.72 M^–1^ × cm^–1^). In comparison, **2(BF_4_)_2_** in DMF shows bands at 270 nm partially overlapped with the solvent subtraction noise, at 325 nm (ε = 2526 M^–1^ × cm^–1^), 365 nm (ε = 2832 M^–1^ × cm^–1^), and 521 nm (ε = 193.6 M^–1^ × cm^–1^).

### Electrochemical Characterization

The redox properties of complexes **1^2+^** and **2^2+^** were investigated by cyclic voltammetry (CV) in anhydrous acetonitrile or dimethylformamide containing 0.1 M [(*n*-Bu)_4_N](PF_6_)(TBAPF_6_) as a supporting electrolyte in a three-electrode one-compartment cell consisting of a glassy carbon working electrode, Pt as a counter electrode, and Ag/AgNO_3_ solution in acetonitrile (0.01 M Ag/AgNO_3_, 0.1 M TBAPF_6_) as a reference electrode, under nitrogen atmosphere. Ferrocene was used as the internal reference, which was added at the end of each experiment.

**Figure 6:**
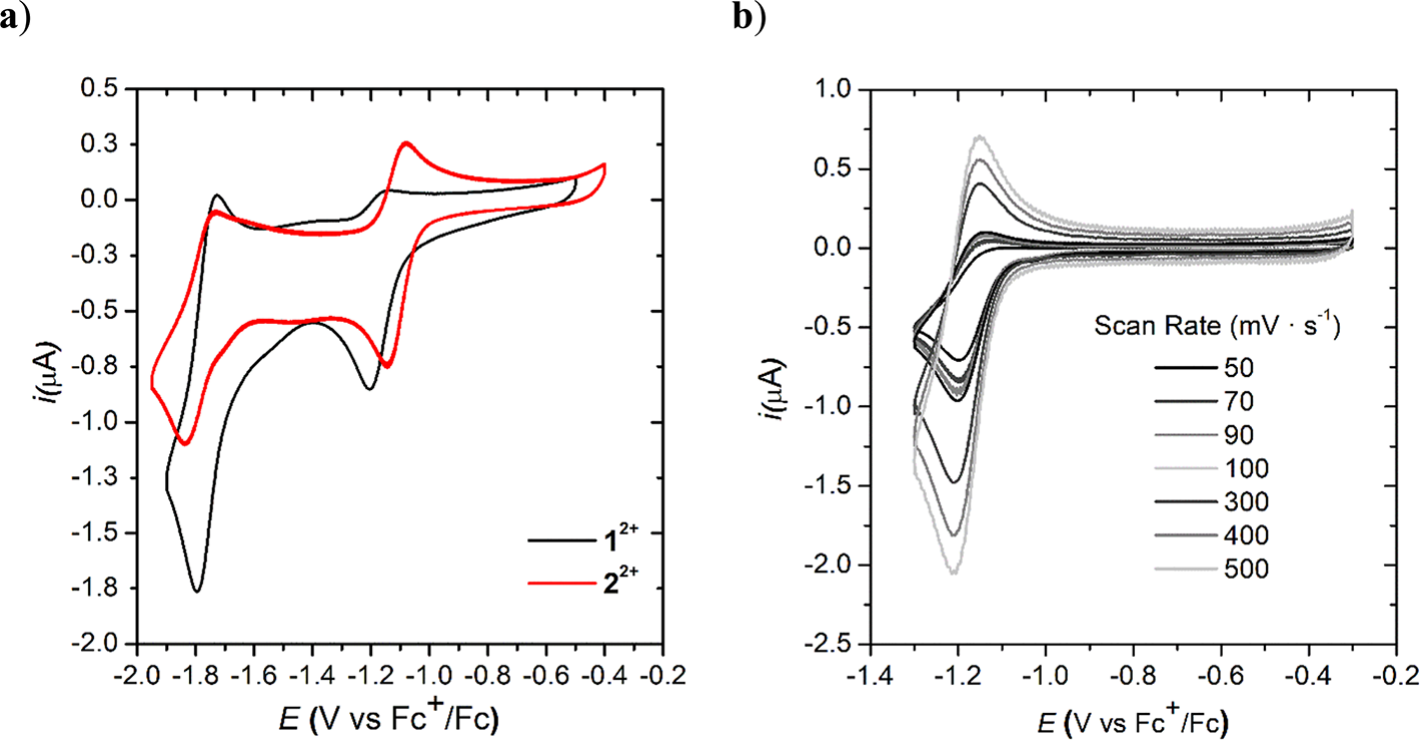
(a) Cyclic voltammetry experiments of 0.5 mM solution of **1^2+^** (black) and **2^2+^** (red) in CH_3_CN and a scan rate of 100 mV/s. (b) Comparison of voltammograms of **1^2+^** in CH_3_CN at different scan rates.

Cyclic voltammetry of **1^2+^** in acetonitrile solution shows two reduction waves. The first one is irreversible with *E*_pc_ = −1.20 V vs Fc^+/0^, and the second one is quasi-reversible with *E*_1/2_ = −1.76 V vs Fc^+/0^ (Δ*E* = 66 mV) (Figure 6a, black trace; see also Table 1 and Figure S33). At higher scan rates (υ ≥ 0.3 V/s), the first reduction peak becomes reversible (*E*_1/2_ = −1.17 V vs Fc^+/0^, Δ*E* = 60 mV; Figure 6b), indicating that a chemical reaction follows the electrochemical step.^30^ A plot of redox peak current varies linearly with the square root of the scan rate (*i_pc_*, *i_pa_* vs υ^1/2^) for both redox events, which is expected from homogeneous diffusion-controlled electrochemical events (Figure S28 in the SI).

**Table 1:** Redox Potential Data for Complexes 1^2+^ and 2^2+^ in CH_3_CN and DMF^b^

solvent	complex	*E*^1^_pa_	*E*^1^_pc_	*E*^1^_1/2_	Δ*E*^1^ (mV)	*E*^2^_pa_	*E*^2^_pc_	*E*^2^_1/2_	Δ*E*^2^ (mV)
CH_3_CN	**1^2+^**	–1.14	–1.20	–1.17	60^a^	–1.73	–1.79	–1.76	66
	**2^2+^**	–1.08	–1.14	–1.11	59	–1.74	–1.83	–1.78	90
DMF	**1^2+^**					–1.72	–1.8	–1.76	80
								
	**2^2+^**					–1.72	–1.8	–1.76	80

a Peak separation potential at high scan rates (υ = 0.3 V/s).

b All potentials are reported in V versus Fc^+/0^.

The electrochemical behavior of complex **2^2+^** is similar to that of **1^2+^** showing a first redox wave at *E*_1/2_ = −1.11 V vs Fc^+/0^ (Δ*E* = 59 mV) and a second one at *E*_1/2_ = −1.78 V vs Fc^+/0^ (Δ*E* = 90 mV), accounting for an anodic shift of 67 mV for the former and only 20 mV cathodic shift for the latter (Figure 6a, red trace; see also Figures S29 and S33 in the SI). Thus, the first redox event from a formal Ni(II) to Ni(I) is influenced by the electron-withdrawing nature of the phenyl ring in the α-position of the diimino complex. The reversibility of this first redox wave at slow scan rates of ≤0.2 V/s is fully reversible for **2^2+^** as opposed to **1^2+^**, suggesting that the chemical reaction following the first reduction is less favored for **2^2+^**.

**Figure 7:**
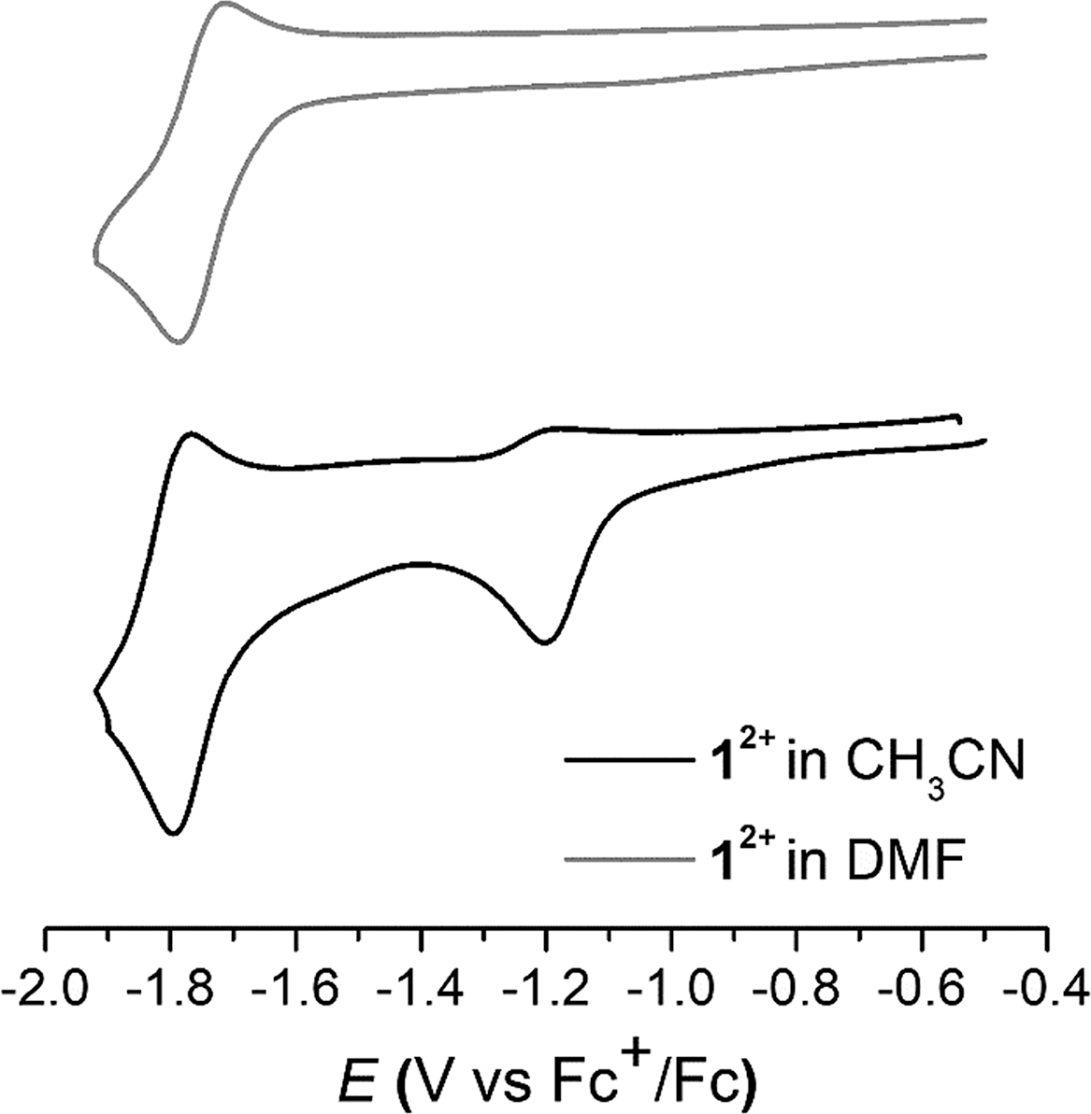
Cyclic voltammogram of **1^2+^** in CH_3_CN (bottom) and DMF (top).

**Figure 8:**
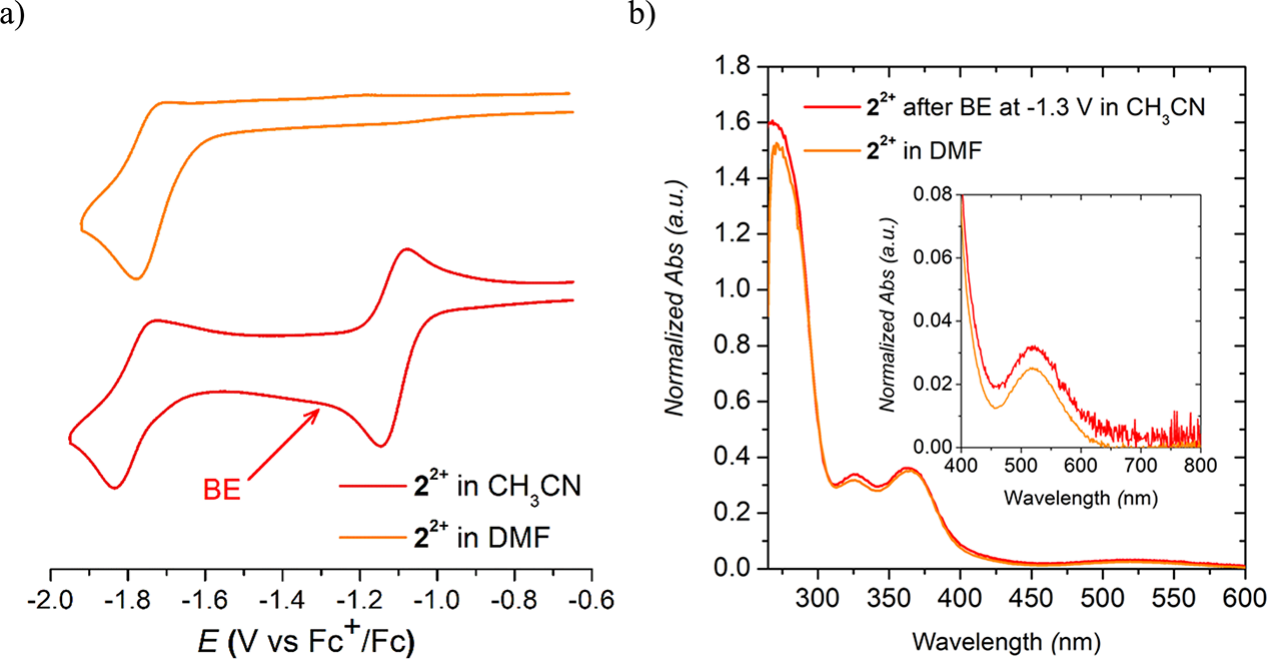
(a) Cyclic voltammogram of **2^2+^** in CH_3_CN (bottom) and DMF (top). The red arrow indicates the applied potential in the bulk electrolysis (BE) experiment (*E*_app_ = −1.3 V vs Fc^+/0^). (b) UV–vis spectra of **2^2+^** after bulk BE at *E*_app_ = −1.3 V vs Fc^+/0^ (red) compared to the UV–vis of (**2-H**)^**+**^ in DMF (orange). See Figure S35 for the details in the BE experiment.

Cyclic voltammetry of the β-diketiminato derivatives (**1-H**)^**+**^ and (**2-H**)^**+**^ in DMF shows only one quasi-reversible peak appearing at the same potential for both complexes with (**2-H**)^**+**^ showing lower reversibility (*E*_1/2_ = −1.76 V vs Fc^+/0^, Δ*E* = 80 mV; Figures 7 and 8a and Table 1; see also Figures S30 and S31 in the SI). Compound (**2-H)^+^** was also characterized by performing a CV of **2^2+^** in acetonitrile in the presence of triethylamine, obtaining the same electrochemical profile (Figure S32). Spectroelectrochemical experiments in an optically transparent thin-layer electrochemical (OTTLE) cell show isosbestic points for the conversion of (**1-H**)^**+**^ and (**2-H**)^**+**^ to the neutral **1-H** and **2-H** derivatives, respectively (Figures S34 and S35 in the SI). The process is reversible, as demonstrated by the full recovery of the initial species in the reverse scan. Thus, the low reversibility of the redox process in DMF is associated to a slow electrochemical process, which is enhanced in the case of the α-phenyl-substituted derivative (**2-H**)^**+**^, suggesting that geometrical rearrangement is involved in the process and is hindered by the bulky phenyl group.

The low potential of the reduction of (**1-H**)^**+**^ and (**2-H**)^**+**^ (*E*_1/2_ = −1.76 V) compared to the first reduction observed for the parent complexes **1^2+^** (*E*_1/2_ = −1.17 V) and **2^2+^** (*E*_1/2_ = −1.11 V) accounts for a cathodic shift of 580 and 650 mV, respectively, and is in agreement with the negatively charged nature of the β-diketiminato versus the neutral β-diimino ligands.

The peak position of the unique redox event observed for (**1-H**)^**+**^ and (**2-H**)^**+**^ exactly matches that of the second redox event observed for **1^2+^** and **2^2+^** (compare black and gray in Figure 7 and red and orange in Figure 8a; see also Table 1). These results suggest a putative conversion of the mono-reduced β-diimino complexes **1^+^** and **2^+^** to the β-diketiminato complexes (**1-H**)^**+**^ and (**2-H**)^**+**^ within the time scale of the experiment. This hypothesis is supported by spectroscopic analysis of the one-electron-reduced species derived from **2^2+^**. This was achieved by performing a bulk electrolysis (BE) experiment at −1.3 V vs Fc^+/0^ of a solution of the β-diimino complex **2^2+^** in acetonitrile (Figure 8b and Figure S37 in the SI). UV–vis analysis of the resulting mixture matches the profile of the spectrum of the β-diketiminato (**2-H**)^**+**^ (Figure 8b). An analogous experiment using compound **1^2+^** leads to decomposition of the complex during the long time of the bulk electrolysis experiment, highlighting the lower stability of this complex compared to the α-phenyl-substituted analogue **2^2+^**.

**Scheme 2:**
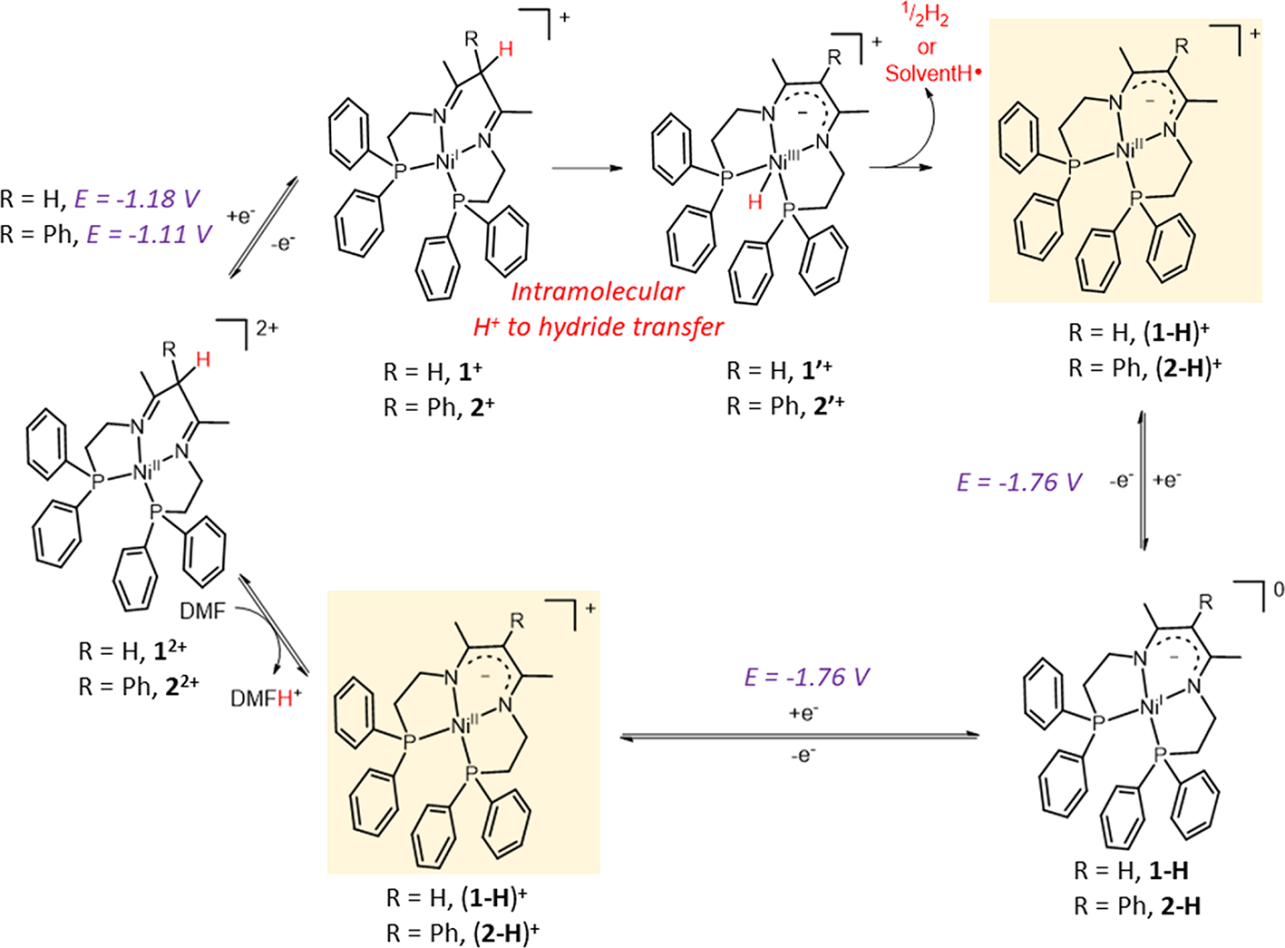
Proposed Electrochemical and Chemical Events during Reduction of **1^2+^** and **2^2+^** in CH_3_CN (top) and DMF (bottom) Showing the Interconversion between β-Diimino and β-Diketiminato Complexes

The conversion of **1^+^** and **2^+^** into (**1-H**)^**+**^ and (**2-H**)^**+**^, respectively, implies a formal loss of a hydrogen atom, which seems unlikely from the product resulting from the one-electron reduction (Scheme 2, top). Instead, we propose a conversion of the mono-reduced formally Ni(I) complexes to a Ni(III) hydride after intramolecular proton transfer from the acidic α-diimino ligand to form the hydride derivative (Ni(III)-H, **1**′^**+**^). Such a transformation of low-valent Ni complexes is ubiquitous in molecular hydrogen evolution catalysis, but it usually takes place in an intermolecular fashion from external acid sources or through an intramolecular proton transfer from pending protonated amino groups.^31^ For complexes **1^2+^** and **2^2+^**, the process is favored by the relatively high acidity of the diimino ligand and close proximity of the two reactive sites.^33^ The resulting Ni(III) hydride could then be involved in a homolytic bimolecular Ni-H cleavage releasing a molecule of H_2_ or to a hydrogen atom abstraction by a solvent molecule via metal hydride hydrogen atom transfer (MHAT) pathways, producing in both cases the β-diketiminato derivatives (**1-H**)^**+**^ and (**2-H**)^**+**^ (Scheme 2, top right).^32−34^ The latter show the same electroreduction feature in CH_3_CN and in DMF at *E*_1/2_ = −1.76 V vs Fc^+^/Fc (Scheme 2, bottom). The transformation of diimino to diketiminate upon reduction and hydrogen atom loss, either by H_2_ evolution or by a MHAT process, evidences the non-innocent nature of this type of ligands that can get involved in cooperative metal–ligand reactivity. A bulk electrolysis experiment of a solution of **2^2+^** at the first one-electron reduction wave (*E*_app_ = −1.2 V vs Fc^+^/Fc) in the absence of any proton source followed by analysis of the headspace of the cathodic compartment by gas chromatography coupled to thermal conductivity detector (GC-TCD) indicates the evolution of hydrogen gas, while no hydrogen gas was detected in an analogous blank experiment performed in the absence of the complex (Figure S36 in the SI). These results not only confirm that the hydrogen atom loss occurs via hydrogen gas production but also indicate a putative involvement of a Ni(III) hydride as a key intermediate for hydrogen production. In this context, a recent report has demonstrated both metal–metal and metal−β-diketiminate ligand cooperative stoichiometric hydrogen evolution by a bimolecular dihydrido nickel complex in the presence of a proton source (Figure 1).^22,23^ Further, the dihydride derivative was stable enough to be isolated and characterized but only in the presence of an acid source, and thanks to the cooperative protonation of the BDK/BDI ligand, the complex was able to evolve H_2_ gas. In contrast, in the case of complexes **1^2+^** and **2^2+^** reported here, the putative hydrido species **1**′^**+**^ and **2**′^**+**^ are too reactive to be isolated, and they rapidly transform into (**1-H**)^**+**^ and (**2-H**)^**+**^, respectively.

### Electrochemical Proton Reduction

The electrochemical behavior of **1^2+^** and **2^2+^** in the presence of acid was performed in CH_3_CN and DMF using *p*-toluene sulfonic acid (*p*-TsOH) (pK_a_ = 8.6 in CH_3_CN).^35^ In acetonitrile solution, the addition of acid results in a slight increase in the current of the first reduction wave for both complexes **1^2+^** and **2^2+^** and a loss of reversibility for both (Figures S40 and S41 in the SI). Upon further addition, the peak intensity continues to increase but quickly reaches a plateau after adding approximately 2–3 equivalents of acid. A similar phenomenon is observed when [DMFH](CF_3_SO_3_) is used as acid (pK_a_ = 6.8 in CH_3_CN; Figure S42 in the SI). The early saturation of the wave intensity suggests an irreversible reaction promoted by the presence of acid but with poor or null hydrogen evolution catalytic nature. Indeed, bulk electrolysis experiments at *E*_app_ = −1.2 V vs Fc^+/0^ in the presence of 20 equivalents of *p*-TsOH·H_2_O did not generate any hydrogen gas, as confirmed by analyzing the headspace of the cathodic compartment of the electrochemical cell using GC-TCD. Thus, we propose that under these conditions, the one-electron-reduced species **1^+^** and **2^+^** or their Ni(III)-hydride derivatives **1**′^**+**^ and **2**′^**+**^ protonate to form the intermediate species (**1 + H**)^2**+**^ and (**2 + H**)^2**+**^ (Scheme S1 in the Supporting Information). Such species could lead to the electroreduction of one or two of the imino ligands to form the amino counterparts (formal hydrogenation of the C=N group). Such transformations have been proposed and even confirmed for related nickel and cobalt complexes containing imino ligands.^36−38^ Since the irreversible conversion of complexes **1^2+^** and **2^2+^** prevented the hydrogen evolution reaction, we did not further investigate this process but focused on the proton reduction ability of **1^2+^** and **2^2+^** in DMF at lower potential.

**Figure 9:**
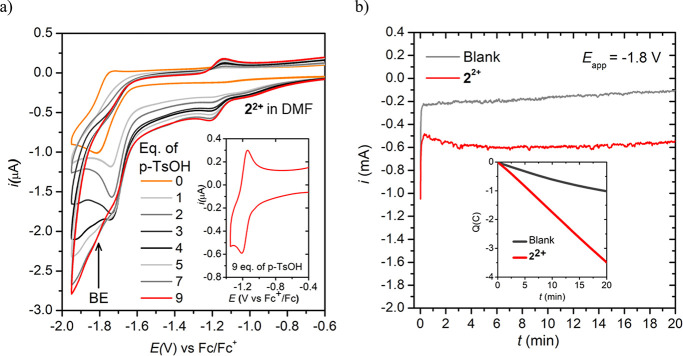
(a) Cyclic voltammetry experiments of a 0.5 mM solution of **2^2+^** in DMF (0.1 M TBAPF_6_) after consecutive addition of *p*-TsOH·H_2_O at a scan rate of 0.1 V/s; WE, GC (Φ = 1 mm); CE, Pt; REF electrode, Ag/AgNO_3_ (Ag wire in a 0.01 M solution of AgNO_3_ and 0.1 M TBAPF_6_ in acetonitrile). (b) Bulk electrolysis experiments at *E*_app_ = −1.8 V vs Fc^+/0^ in the presence of 60 eq. *p*-TsOH·H_2_O in the presence (red) and in the absence (gray) of a 0.5 mM solution of **2^2+^** in DMF (0.1 M TBAPF_6_); WE, GC rod (*S* = 3.1 cm^2^); CE, Pt mesh; REF electrode, Ag/AgNO_3_ (Ag wire in a 0.01 M solution of AgNO_3_ and 0.1 M TBAPF_6_ in acetonitrile), *V*_cathode_ = *V*_anode_ = 5 mL.

**Scheme 3:**
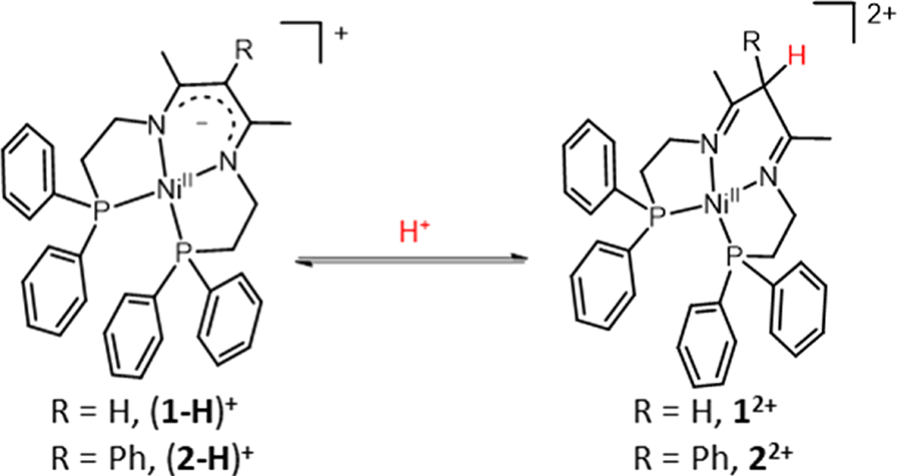
Equilibria between β-Diketiminato and β-Diimino Complexes (**1-H**)^**+**^/**1^2+^** and (**2-H**)^**+**^/**2^2+^**, Highly Dependent on Acid Concentration and Acid Strength

Figure 9a shows the cyclic voltammetry profile after successive additions of *p*-TsOH in a solution of **2^2+^** in DMF. The reversible wave at −1.8 V associated with the one-electron reduction of (**1-H**)^**+**^ and (**2-H**)^**+**^ becomes irreversible and increases in intensity with increasing concentration of acid. In addition, an increase in the current is also observed at *E* < −1.8 V, which cannot be attributed solely to the blank (see Figure S43 in the SI). A characteristic reversible wave at *E* = −1.1 V is also observed, attributed to the redox signature of the first reduction of **2^2+^**, which results from the protonation of (2**-H**)^**+**^ after a few additions of acid. This phenomenon is very clear at 9 equivalents of *p*-TsOH (Figure 9a, inset). These results show how subtle changes in acid concentration and acid strength can displace the equilibrium between the β-diimino and β-diketiminato complexes (Scheme 3).^39−41^ A very similar result is obtained using compound **1^2+^**, as shown in Figure S44 in the SI.

To assess the proton reduction ability of **1^2+^** and **2^2+^** in DMF, a bulk electrolysis experiment at *E*_app_ = −1.8 V vs Fc^+/0^ in the presence of 60 equivalents of *p*-TsOH was performed. After 20 min, the headspace of the electrochemical cell was analyzed by GC-TCD, confirming the formation of hydrogen gas. The accumulated charge and accumulated hydrogen gas in the bulk electrolysis experiments are significantly higher than those observed for analogous experiments performed in the absence of complexes, confirming the role of **1^2+^** and **2^2+^** in promoting the hydrogen evolution reaction (Figure 9b and Figures S44b and S47 in the SI). Taking into account the pK_a_ of *p*-TsOH in DMF, an approximate overpotential of 0.78 V is calculated for the HER by **1^2+^** and **2^2+^** (see the Supporting Information).^42,43^ Complex **2^2+^** shows a fairly stable current over the whole 20 min period at about −0.60 mA (−0.19 mA/cm^2^), and complex **1^2+^** shows a lower current intensity (−0.36 mA, −0.11 mA/cm^2^) that starts to decrease slowly after 8 min, suggesting a higher stability for the former. Cyclic voltammetry experiments performed immediately after the bulk electrolysis experiments show the characteristic redox feature of complex **2^2+^** at *E* = −1.1 V (after 1.8 turnover numbers), while complex **1^2+^** is more difficult to identify due to the irreversible nature of the first reduction, which shows to be very broad and pointing to partial decomposition (Figure S45 in the SI). Faradaic efficiencies of 33 and 35% were obtained for **1^2+^** and **2^2+^**, respectively. A rinse test of the working electrode performed after the bulk electrolysis experiment suggests that part of the decomposition might be related to deposited species on the electrode since a broad reduction wave in the range of *E* = −1.1 to −1.7 V vs Fc^+/0^ is observed in both cases (**1^2+^** and **2^2+^**) and that might be responsible for the slight increase of current in this range of potentials (Figure S46 in the SI). Importantly, no significant increase in the current is observed at the bulk electrolysis potential (*E*_app_ = −1.8 V vs Fc^+/0^) for the rinsed electrode when compared to a blank electrode, supporting the homogeneous molecular nature of the catalytic process.

The fact that **1^2+^** and **2^2+^** are able to trigger hydrogen evolution in the presence of protons in DMF at *E*_app_ = −1.8 V suggests that at this low potential, the decomposition pathways observed at lower potentials and attributed to the reduction of the imine motifs are avoided. Thus, the reduction of the key hydride species **1**′^**+**^ and **2**′^**+**^ in Scheme 2 is proposed as the driving force of the hydrogen evolution reaction through the formal Ni(II)-hydride species **1**′ and **2**′ (see also Scheme S2 in the Supporting Information). Alternatively, the formal Ni(I) diimino derivatives **1^+^** and **2^+^** could also be reduced to formal Ni(0) complexes that could lead to hydrogen, most likely through a Ni(I)-hydride complex. However, the latter route seems less likely taking into account the possible conversion of the involved β-diimino species to the β-diketiminato derivatives by fast intramolecular proton to hydride transfer.

## Conclusions

The spectroscopic and electrochemical characterization of the two novel nickel κ^4^-P_2_N_2_ β-diimino (BDI) complexes **1^2+^** and **2^2+^** shows the interconversion of the BDI ligand to a β-diketiminato (BDK) ligand upon reduction and hydrogen atom loss. Two pathways are possible for this transformation, both starting from a metal-based reduction of the initial Ni(II) in **1^2+^** and **2^2+^** to form a formal Ni(I) species that rapidly evolves to the Ni(III)-hydride derivatives **1**′^**+**^ and **2**′^**+**^ upon intramolecular deprotonation of the α-carbon acidic proton in the BDI ligand. At this point, **1**′^**+**^ and **2**′^**+**^ can be involved either in hydrogen evolution through bimolecular reductive elimination or in the metal hydride-induced hydrogen atom transfer (MHAT) reaction to the acetonitrile solvent. Both routes would lead to the formation of the BDK complexes (**1**-H)^**+**^ and (**2**-H)^**+**^. A bulk electrolysis experiment of **2^2+^** in the absence of acid at the first reduction wave potential leads to hydrogen gas formation detected in the headspace of the cathodic compartment, proving that the bimolecular reductive elimination is the preferred pathway.

Hydrogen detection experiments in the presence of acid sources rule out a hydrogen evolution reaction in the first reduction wave of **1^2+^** and **2^2+^** (*E*_app_ = −1.2 V vs Fc^+/0^), presumably due to the electroreduction of the imino ligands that evolve to the amino derivatives in the presence of protons and blocking the reductive elimination route toward H_2_. In contrast, hydrogen evolution is demonstrated in the second reduction wave where the decomposition route is partially overcome by further reduction of intermediates **1**′^**+**^ and **2**′^**+**^ at low potentials (*E*_app_ = −1.8 V vs Fc^+/0^). Importantly, the conversion of (**1**-H)^**+**^ and (**2**-H)^**+**^ to **1^2+^** and **2^2+^**, respectively, in the presence of acid is clearly demonstrated by the recovery of the first reduction wave of **2^2+^** in DMF. Overall, these results highlight the easy interconversion between BDI and BDK ligands, depending on subtle changes in the acid strength of species in the media and their concentration and manifest their non-innocent character as well as their potential role as proton relays in catalytic processes.

## Experimental Section

All organic reagents and metal precursors were purchased from Sigma-Aldrich and used without further purification, unless otherwise stated. Anhydrous solvents were taken from a solvent purification system (SPS).

### Synthesis of 4-((2-(Diphenylphosphaneyl)ethyl)amino)pent-3-en-2-one

A round-bottom flask equipped with a Dean–Stark apparatus was charged with a catalytic amount of *p*-toluene sulfonic acid (0.04 g) and the setup subjected to vacuum–nitrogen cycles to provide an inert atmosphere. A solution of acetyl acetone (0.43 g, 4.36 mmol) in 20 mL of dry and degassed toluene was added by cannula, and the mixture was stirred under nitrogen atmosphere at RT. After 1 h of stirring, 2-diphenylphosphinoethylamine (1 g, 4.36 mmol) in 20 mL of dry and degassed toluene was added, and the reaction mixture was warmed up to reflux under nitrogen atmosphere. After 24 h, the reaction was cooled down to room temperature, and the volatiles were removed by reduced pressure, resulting in a yellow oily compound, identified as the desired compound by NMR spectroscopy (1.35 g, 4.36 mmol, 100%). ^1^H NMR (500 MHz, C_6_D_6_) δ 11.36 (s, 1H), 7.38–7.29 (m, 4H), 7.16–7.08 (m, 6H), 4.92 (s, 1H), 3.00–2.91 (m, 2H), 2.13 (s, 3H), 2.05–1.95 (m, 2H), 1.32 (s, 3H). ^13^C NMR (126 MHz, C_6_D_6_) δ 194.6, 161.5, 138.6–138.5 (m), 133.2, 95.8, 40.2, 29.1, 18.3. ^31^P{H} NMR (202 MHz, C_6_D_6_) δ −18.22.

### Synthesis of (2,4)-*N*-(2-(Diphenylphosphaneyl)ethyl(diphenylphosphaneyl)ethyl)imino)pent-2-en-2-amine, HBDI

Inside the glovebox, 4-((2-(diphenylphosphaneyl)ethyl)amino)pent-3-en-2-one (1 g, 3.21 mmol) and dimethyl sulfate (0.4 mL, 4.4 mmol) were stirred for 30 min. During this time, fume appeared and the reaction mixture became dense. The reaction mixture was maintained overnight without stirring. Then, 2-diphenylphosphinoethylamine (1 g, 4.36 mmol) in 2 mL of dry and degassed methanol was added and stirred, forming a viscous red oil. After 3 h, sodium methoxide (0.17 g 3.21 mmol) dissolved in 10 mL of dry and degassed methanol was added and immediately after the mixture became cloudy and a white precipitate started to appear. The reaction stirring continued for another 3 h. The white precipitate was filtered, washed with 100 mL of MeOH, and dried under vacuum (500 mg, 0.96 mmol, 30%). ^1^H NMR (500 MHz, C_6_D_6_) δ 7.59–7.49 (m, 8H), 7.16–7.07 (m, 12H), 4.63 (s, 1H), 3.49–3.37 (m, 4H), 2.59–2.52 (m, 4H), 1.66 (s, 6H). ^13^C NMR (126 MHz, C_6_D_6_) δ 160.1, 139.9, 139.8, 133.4, 133.39, 95.4, 44.0, 43.8, 31.6 (d, *J* = 10.7 Hz), 19.41. ^31^P{H} NMR (202 MHz, C_6_D_6_) δ −17.26. Anal. Calcd: C, 75.84; H, 6.94; N, 5.36. Found: C, 75.04; H, 6.62; N, 5.26; ESI-Mass: calcd for [C_33_H_36_N_2_P_2_] = 522.2. Found: [M + H]^+^*m*/*z*: 523.2.

### Synthesis of 4-((2-(Diphenylphosphaneyl)ethyl)amino)-3-phenylpent-3-en-2-one

A round-bottom flask equipped with a Dean–Stark apparatus containing a catalytic amount of *p*-toluene sulfonic acid (0.04 g) and 3-phenyl-2,4-pentanedione (0.76 g, 4.36 mmol) was subjected to vacuum–nitrogen cycles to provide an inert atmosphere. Dry and degassed toluene (20 mL) was added by a cannula, and the mixture was stirred under nitrogen atmosphere at RT. After 1 h, a solution of 3-diphenylphosphinoethylamine (1 g, 4.36 mmol) in 20 mL of dry and degassed toluene was added, and the reaction mixture was heated up to reflux under nitrogen. After 24 h, refluxing of the reaction was stopped, and the volatiles were removed under reduced pressure. The resulting yellow oil was identified as the desired compound (1.2 g, 3.48 mmol, 80%). ^1^H NMR (500 MHz, C_6_D_6_) δ 12.63 (s, 1H), 7.41–7.33 (m, 3H), 7.27–7.20 (m, 3H), 7.20–7.06 (m, 9H), 3.12–3.03 (m, 2H), 2.21–2.11 (m, 2H), 2.08 (s, 3H), 1.30 (s, 3H). ^13^C NMR (126 MHz, C_6_D_6_) δ 194.2, 160.6, 142.0, 141.8, 138.9, 138.7, 138.5, 133.2, 133.0, 132.7, 132.6 (d, *J* = 2.6 Hz), 131.6, 131.5–131.2 (m), 131.0, 130.9, 129.1, 128.8 (dd, *J* = 13.6, 6.7 Hz), 126.9, 126.6, 109.9, 40.8, 40.6, 37.3, 37.1, 37.0, 32.3, 30.2, 29.9, 29.8, 29.5, 29.2, 29.1, 23.1, 22.8, 21.2, 16.4, 16.2, 14.4, 1.4. ^31^P{H} NMR (202 MHz, C_6_D_6_) δ −18.04.

### Synthesis of (2,4)-*N*-(2-(Diphenylphosphaneyl)ethyl)-4-((2-(diphenylphosphaneyl)ethyl)imino)-3-phenylpent-2-en-2-amine, PhBDI

Inside the glovebox, (2,4)-*N*-(2-(diphenylphosphaneyl)ethyl)-4-((2-(diphenylphosphaneyl)ethyl)imino)-3-phenylpent-2-en-2-amine (1.4 g, 4.36 mmol) and dimethyl sulfate (0.4 mL, 4.36 mmol) were stirred for 30 min. During this time, fume appeared, and the mixture became dense. The reaction mixture was maintained overnight without stirring, producing a very dense red-orange oily mixture. A solution of 2-diphenylphosphinoethylamine (1 g, 4.36 mmol) in 2 mL of dry and degassed methanol was added. 20 min after the addition, a white dense solid appeared, which was diluted in 2 mL of methanol to make the reaction mixture easier to stir. After 3 h, a solution of sodium methoxide (0.23 g, 4.36 mmol) in 10 mL of dry and degassed methanol was added and immediately a white precipitate appeared. Stirring continued for another 3 h. The resulting white solid was filtered off, washed with 100 mL of MeOH, and dried under vacuum (900 mg, 1.53 mmol, 35%). ^1^H NMR (500 MHz, C_6_D_6_) δ 13.27 (s, 1H), 7.60–7.50 (m, 7H), 7.29–7.19 (m, 8H), 7.19–7.05 (m, 10H), 3.52 (dt, *J* = 9.6, 7.4 Hz, 4H), 2.63 (dd, *J* = 8.2, 6.6 Hz, 4H), 1.57 (s, 6H). ^13^C NMR (126 MHz, C_6_D_6_) δ 160.2, 144.4, 139.9, 139.8, 133.4, 133.3 (d, *J* = 5.5 Hz), 126.2, 44.3, 44.1, 31.6 (d, *J* = 10.6 Hz), 17.8. ^31^P{H} NMR (202 MHz, C_6_D_6_) δ −17.19. Anal. Calcd: C, 78.24; H, 6.73; N, 4.68. Found: C, 78.18; H, 6.66; N, 4.53; ESI-Mass: calcd for *m*/*z*: [C_31_H_40_N_2_P] = 598.3. Found [M + H]^+^*m*/*z*: 599.29.

### Synthesis of 1(BF_4_)_2_

In a round-bottom flask equipped with a reflux condenser, Nickel(II) tetrafluoroborate hexahydrate (60 mg, 0.177 mmol) was dissolved in 30 mL of anhydrous and degassed acetonitrile affording a blue solution. Ligand HBDI (100 mg, 0.177 mmol) was dissolved in 15 mL of DCM inside the glovebox, and the resulting yellow solution was transferred to the dissolved nickel salt solution by a cannula outside the glovebox. Upon addition, the color of the reaction changed from blue to red. The reaction mixture was stirred at 65 °C under nitrogen overnight. The volatiles were removed under reduced pressure, leaving a red oily compound, which was dissolved in the minimum amount of acetonitrile. To this acetonitrile solution, Et_2_O was added, and the color of the solution became cloudy. It was placed at −30 °C, causing green crystals to appear that were filtered and washed with Et_2_O (40 mg, 0.0637 mmol, 36%). Crystals suitable for single-crystal X-ray diffraction analysis were grown by slow diffusion of Et_2_O to a saturated acetonitrile solution. ^1^H NMR (500 MHz, CD_3_CN) δ 7.55 (t, *J* = 7.5 Hz, 3H), 7.47–7.43 (m, 9H), 7.35 (t, *J* = 7.6 Hz, 8H), 4.29 (s, 2H), 3.66 (s, 4H), 2.74–2.68 (m, 4H), 2.27 (s, 6H). ^13^C NMR (126 MHz, CD_3_CN) δ 180.0, 133.9, 133.2, 129.8117.6, 53.7, 52.7, 23.4. ^31^P{H} NMR (202 MHz, CD_3_CN) δ 49.64.

^1^H NMR (500 MHz, DMF-d_7_) δ 7.63 (ddd, *J* = 8.2, 6.7, 3.1 Hz, 8H), 7.55 (t, *J* = 7.5 Hz, 4H), 7.36 (t, *J* = 7.6 Hz, 8H), 5.31 (s, 1H), 3.45 (ddt, *J* = 19.6, 13.3, 6.5 Hz, 4H), 2.88 (p, *J* = 6.4 Hz, 4H), 2.22 (s, 6H). ^13^C NMR (126 MHz, DMF) δ 135.1 (t, *J* = 5.2 Hz), 133.2, 130.1, 128.3–126.9 (m), 102.2, 52.0, 33.8, 24.6. ^31^P{H} NMR (202 MHz, DMF(d_7_)) δ 57.64. Anal. Calcd: [C_33_H_36_N_2_NiP_2_](BF_4_)_2_ + Et_2_O: C, 52.55; H, 5.17; N, 3.51. Found: [C_33_H_36_N_2_NiP_2_](BF_4_)_2_ + Et_2_O: C, 52.30; H, 4.60; N, 3.50; ESI-Mass: calcd for *m*/*z*: [C_33_H_36_N_2_NiP_2_]^2+^ = 580.2. Found: [M – H]^+^*m*/*z*: 579.16.

### Synthesis of 2(BF_4_)_2_

In a round-bottom flask equipped with a reflux condenser, Nickel(II) tetrafluoroborate hexahydrate (56 mg, 0.167 mmol) was dissolved in 30 mL of anhydrous and degassed acetonitrile under nitrogen affording a blue solution. Ligand PhBDI (100 mg, 0.167 mmol) was dissolved in 15 mL of DCM inside the glovebox, and the resulting yellow solution was transferred to the nickel salt solution by a cannula outside the glovebox. Upon the addition of the ligand, the color of the reaction changed from blue to red. The reaction mixture was heated to 65 °C under nitrogen overnight. Removing volatiles under reduced pressure afforded a red oily compound, which was dissolved in the minimum amount of acetonitrile. To this acetonitrile solution, Et_2_O was added, which caused the precipitation of a yellow solid that was filtered and washed with Et_2_O (80 mg, 0.16 mmol, 96%). Crystals suitable for single-crystal X-ray diffraction analysis were grown by slow diffusion of Et_2_O to an acetonitrile solution of **2(OTf)_2_**. ^1^H NMR (500 MHz, CD_3_CN) δ 7.76–7.70 (m, 2H), 7.66–7.54 (m, 4H), 7.52–7.26 (m, 16H), 7.26–7.19 (m, 3H), 5.50 (s, 1H), 3.75 (ddt, *J* = 29.1, 14.8, 8.0 Hz, 5H), 2.97 (dp, *J* = 12.6, 6.9 Hz, 2H), 2.62 (dt, *J* = 13.9, 7.0 Hz, 3H), 2.31 (d, *J* = 0.9 Hz, 7H). ^13^C NMR (126 MHz, CD_3_CN) δ 181.52, 133.6–133.5 (m), 133.4, 133.1, 130.6, 130.2, 129.7, 128.9, 117.6, 68.0, 53.0, 38.3, 28,3, 23.2. ^31^P{H} NMR (202 MHz, CD_3_CN) δ 47.15.

^1^H NMR (500 MHz, DMF-d7) δ 8.04 (s, 2H), 7.70–7.33 (m, 10H), 7.22–7.14 (m, 1H), 3.72 (s, 14H), 3.56 (dt, *J* = 29.9, 6.2 Hz, 1H), 3.56 (s, 1H), 2.97–2.89 (m, 2H), 2.80–2.74 (m, 3H), 1.95 (s, 2H). ^13^C NMR (101 MHz, DMF) δ 144.8, 134.1, 132.2 ,130.1–128.5, 128.3–125.6, 113.0, 52.1, 34.26, 23.3. ^31^P{H} NMR (162 MHz, DMF-d_7_) δ 57.87. Anal. Calcd: [C_39_H_40_N_2_NiP_2_](BF_4_)_2_ + Et_2_O: C, 56.21; H, 5.18; N, 3.37. Found: [C_39_H_40_N_2_NiP_2_](BF_4_)_2_ + Et_2_O: C, 56.20; H, 5.10; N, 3.20; ESI-Mass: calcd for *m*/*z*: [C_39_H_40_N_2_NiP_2_]^2+^ = 656.2. Found: [M – H]^+^ = 655.0.

## Supporting Information Available

Additional experimental details, materials, and methods including NMR, UV–vis spectra, and (spectro)electrochemical data (PDF)
