# Supplementary material for: Interplay between β-Diimino and β-Diketiminato Ligands in Nickel Complexes Active in the Proton Reduction Reaction
Source: Inorg Chem. 2022 Oct 5;61(42):16639–49. doi: 10.1021/acs.inorgchem.2c02150 (PMC9597662; doi:10.1021/acs.inorgchem.2c02150)
Supplement: Supplementary file 1 — ic2c02150_si_001.pdf [file ic2c02150_si_001.pdf]

Interplay between β-diimino and β-diketiminato ligands in nickel complexes active in the proton reduction reaction

Navid Jameei-Moghaddam;[1,2] Marcos Gil-Sepulcre,[1] Jia-Wei Wang,[1] Jordi Benet-Buchholz,[1] Carolina Gimbert-Suriñach,[1,3]* Antoni Llobet[1,3]*

[1]Institute of Chemical Research of Catalonia (ICIQ), Barcelona Institute of Science and Technology (BIST), Avda. Països Catalans 16, 43007 Tarragona, Spain.

[2]Departament de Química Física i Inorgànica, Universitat Rovira i Virgili, Marcel·lí Domingo s/n, 43007 Tarragona, Spain.

[3]Departament de Química, Universitat Autònoma de Barcelona, Cerdanyola del Vallès, 08193 Barcelona, Spain.

Corresponding authors: carolina.gimbert@uab.cat; allobet@iciq.cat

Supporting information

Table of contents

	Page
1. General methods and instrumentations	S3
2. NMR Spectra of $\mathbf{1}^{2+}$ and $\mathbf{2}^{2+}$	S6
3. Electrochemistry and Spectroelectrochemistry of $\mathbf{1}^{2+}$ and $\mathbf{2}^{2+}$	S20
4. UV-Vis Spectroscopy of $\mathbf{1}^{2+}$ and $\mathbf{2}^{2+}$	S28
5. Electrochemistry and Gas Detection Experiments in the presence of acid	S29
5.1 Determination of faradic efficiency, Turnover Number (TON), Turnover Frequency (TOF) and overpotential of the HER in DMF.	S33
6. Additional Schemes	S38

1- General Methods and Instrumentations:

1.1- Crystallography data collection:

Measured crystals were prepared under inert conditions immersed in perfluoropolyether as protecting oil for manipulation.

Crystal structure determination for **1(BF$_4$)$_2$** and **2(BF$_4$)$_2$** was carried out using an Apex DUO Kappa 4-axis goniometer equipped with an APPEX 2 4K CCD area detector, a Microfocus Source E025 IuS using MoK$_\alpha$ radiation, Quazar MX multilayer Optics as monochromator and an Oxford Cryosystems low temperature device Cryostream 700 plus (T = -173 °C). Full-sphere data collection was used with ω and φ scans. *Programs used:* Data collection APEX-2[1], data reduction Bruker Saint[2] V/.60A and absorption correction SADABS[3]

1.2- Structure solution and refinement

Crystal structure solution was achieved using the computer program SHELXT[4] . Visualization was performed with the program SHELXle[5]. Missing atoms were subsequently located from difference Fourier synthesis and added to the atom list. Least-squares refinement on F^2 using all measured intensities was carried out using the program SHELXL 2015[6]. All non-hydrogen atoms were refined including anisotropic displacement parameters.

1.3- Comments to the structure: Compound 1(BF$_4$)$_2$

The asymmetric unit contains one molecule of the Nickel metal-complex, two BF_4^- anions and 0.15 molecules of water. In the main molecule three of the aromatic rings are disordered in two orientations (approximated ratio 60:40). The BF_4^- anions are also disordered in two orientations. Additionally one of the disordered BF_4^- anions is sharing its position

[1] Data collection with APEX II version v2013.4-1. Bruker (2007). Bruker AXS Inc., Madison, Wisconsin, USA.
[2] Data reduction with Bruker SAINT version V8.30c. Bruker (2007). Bruker AXS Inc., Madison, Wisconsin, USA.
[3] SADABS: V2012/1 Bruker (2001). Bruker AXS Inc., Madison, Wisconsin, USA. Blessing, Acta Cryst. **1995**, A51, 33-38.
[4]SHELXT; V2018/2. Sheldrick, G.M. *Acta Cryst*. **2015** A71, 3-8.
[5]SHELXle; C.B. Huebschle, G.M. Sheldrick & B. Dittrich; J.Appl.Cryst. (2011) 44, 1281-1284.
[6] SHELXL; SHELXL-2018/3. Sheldrick, G.M. *Acta Cryst*. **2015** C71, 3-8.

(coordinated with the disorder) with 0.15 molecules of water.

Compound 1(BF$_4$)$_2$: The asymmetric unit contains one molecule of the cationic metal-complex and two BF$_4$ anions.

1.4- *Electrochemical methods and instrumentations*:

Glassy Carbon electrodes (1mm diameter), Platinum Disk Electrodes, and reference electrodes (AgNO$_3$/Ag) were purchased from IJ-Cambria Ldt. Pads and alumina for polishing were also purchased from the same company. Cyclic voltammetry experiments were performed with a CHI660D potentiostat in a one-compartment cell, three electrode system using glassy carbon (GC) disk as working electrode, platinum disk as counter electrode and standard calomel electrode (SCE) or AgNO$_3$/Ag reference electrodes.

1.5- *Spectro electrochemistry*:

Spectroelectrochemistry experiments were performed in a custom-made Optically Transparent Thin Layer Electrochemical (OTTLE) cell (University of Reading) with platinum mesh working electrode, platinum wire counter electrode and silver wire pseudo-reference electrode.

1.6- UV-vis equipment:

UV-Vis measurements were carried out on a Lambda 1050 PerkinElmer spectrophotometer equipped with a PMT, InGaAs and PbS detectors system, double beam optics, double monochromator and D2 and W light sources.

1.7- High-resolution mass spectrometry:

(HRMS) was recorded on a Thermo Finnigan MAT95XL spectrometer (for CI) and a ThermoFisher Scientific LTQ-Orbitrap spectrometer (for ESI).

1.8- NMR spectroscopy of ligands HBDI and PhBDI

^{1}H and ^{13}C NMR spectra were recorded on a *Bruker Avance 400* spectrometer fitted with a 5 mm i.d. BBFO+ probe carefully tuned to the recording frequency of 400.13 MHz (for ^{1}H) and 100.61 MHz (for ^{13}C). The spectra are referenced to the solvent in which they were run (7.26 ppm for ^{1}H CDCl$_3$ and 77.16 ppm for ^{13}C CDCl$_3$, 2.5 ppm for ^{1}H DMSO, and

39.52 ppm for ^{13}C DMSO). Chemical shifts (δ) are given in ppm, coupling constants (J) are given in Hz with the following splitting abbreviations: s = singlet, d = doublet, t = triplet, q = quartet, qt = quintet, sx = sextuplet, sp = septuplet, m = massif and br = broad. All assignments were confirmed with the aid of two-dimensional ^{1}H, ^{1}H (COSY), or ^{1}H, ^{13}C (HSQC, HMBC) experiments using standard pulse programs.

1.9- NMR Spectroscopy of Ni complexes

^{1}H NMR, ^{13}C NMR, ^{31}P NMR, and spectra were recorded on a Bruker 400 MHz or a Bruker 500 MHz. Chemical shifts (δ) are reported in ppm and peak multiplicity is designated as s (singlet), d (doublet), t (triplet), m (multiplet), dd (doublet of doublets), dt (doublet of triplets), tt (triplet of triplets) and br (broad). All the collected spectra were referenced on residual solvent signal according to Nudelman et al.[2] Aqueous samples were recorded on an NMR spectrometer Bruker Avance 500 MHz spectrometer equipped with a Cryoprobe™ and denoted with a "*" at the synthetic description.

2- NMR Spectra of 1(BF_4)$_2$ and 2(BF_4)$_2$

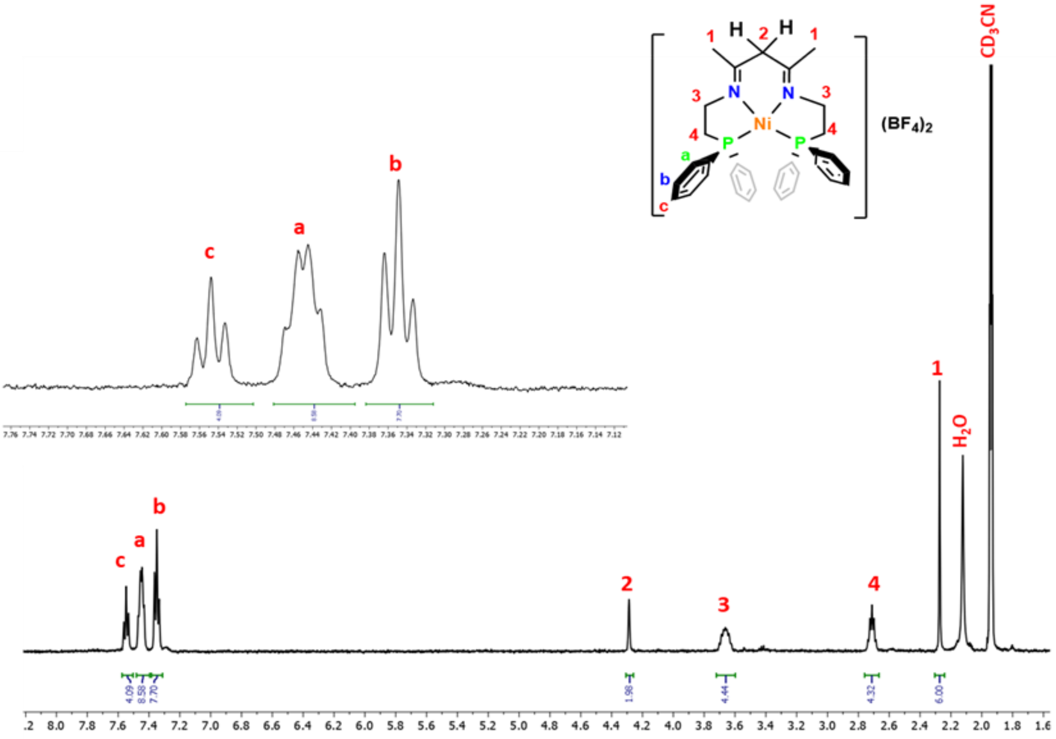

Figure S1. ^{1}H NMR of complex **1(BF_4)$_2$** in CD_3CN

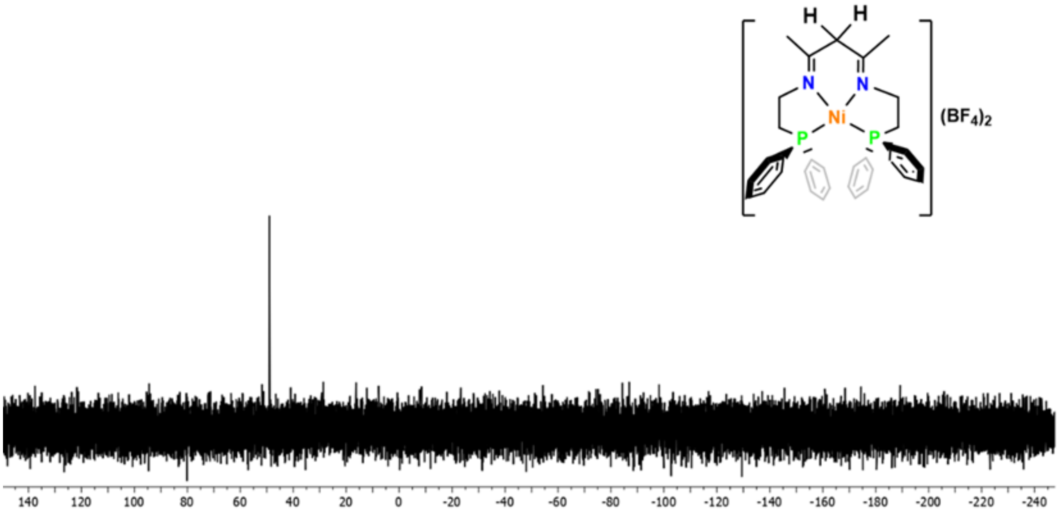

Figure S2. ^{31}P{H} NMR of complex **1(BF_4)$_2$** in CD_3CN.

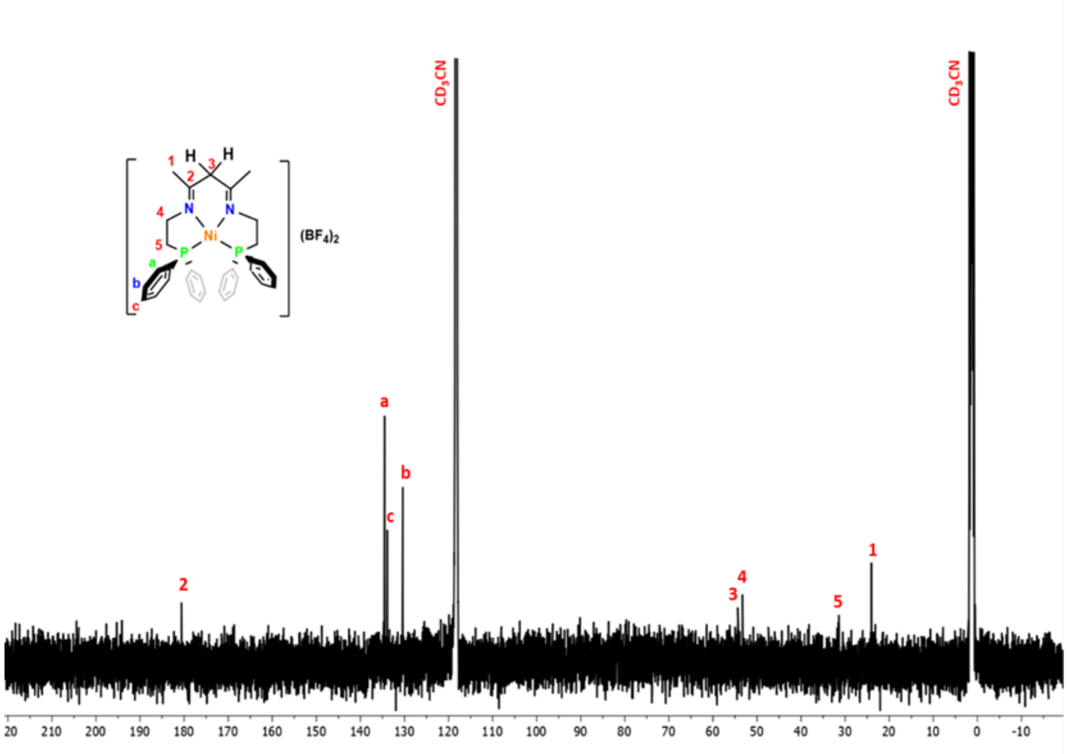

Figure S3. ^{13}C NMR of complex **1(BF$_4$)$_2$** in CD_3CN

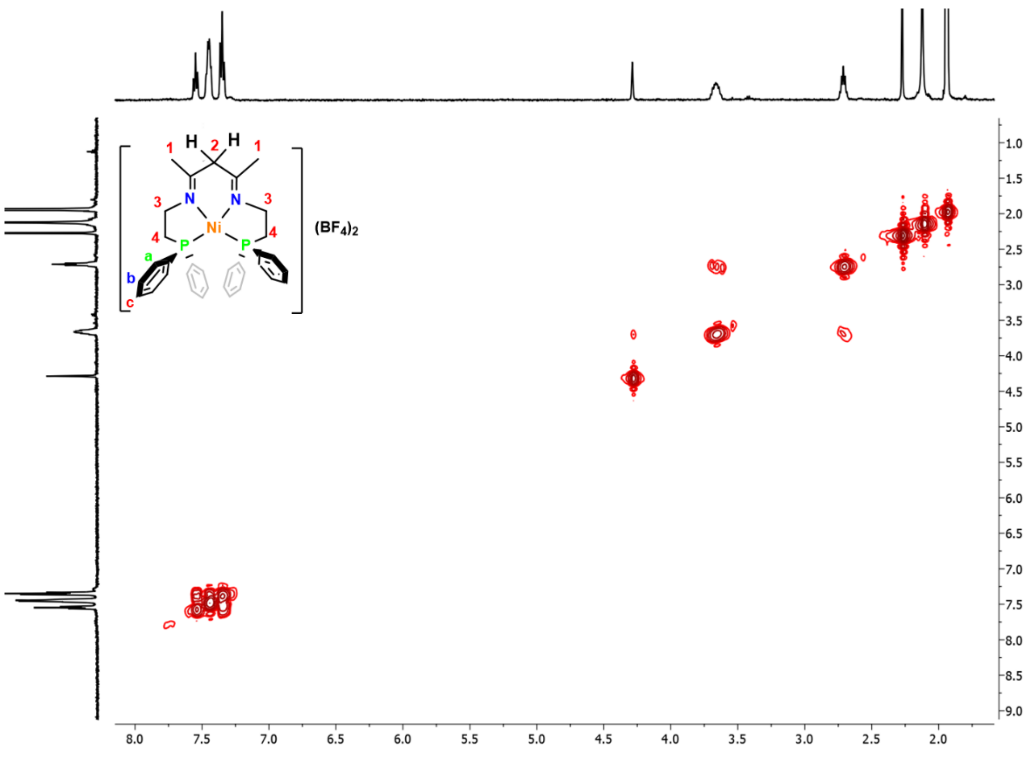

Figure S4. ^{1}H-^{1}H COSY NMR of **1(BF$_4$)$_2$** complex in CD_3CN

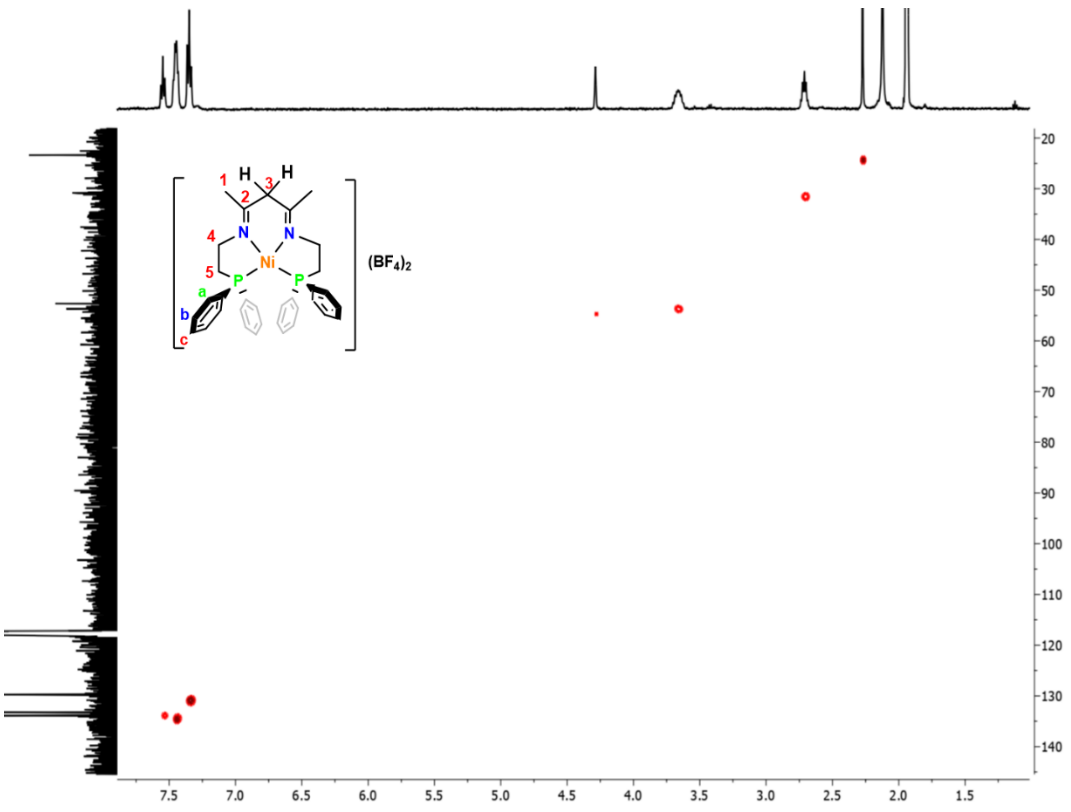

Figure S5. HSQC NMR of **1(BF4)2** complex in CD_3CN

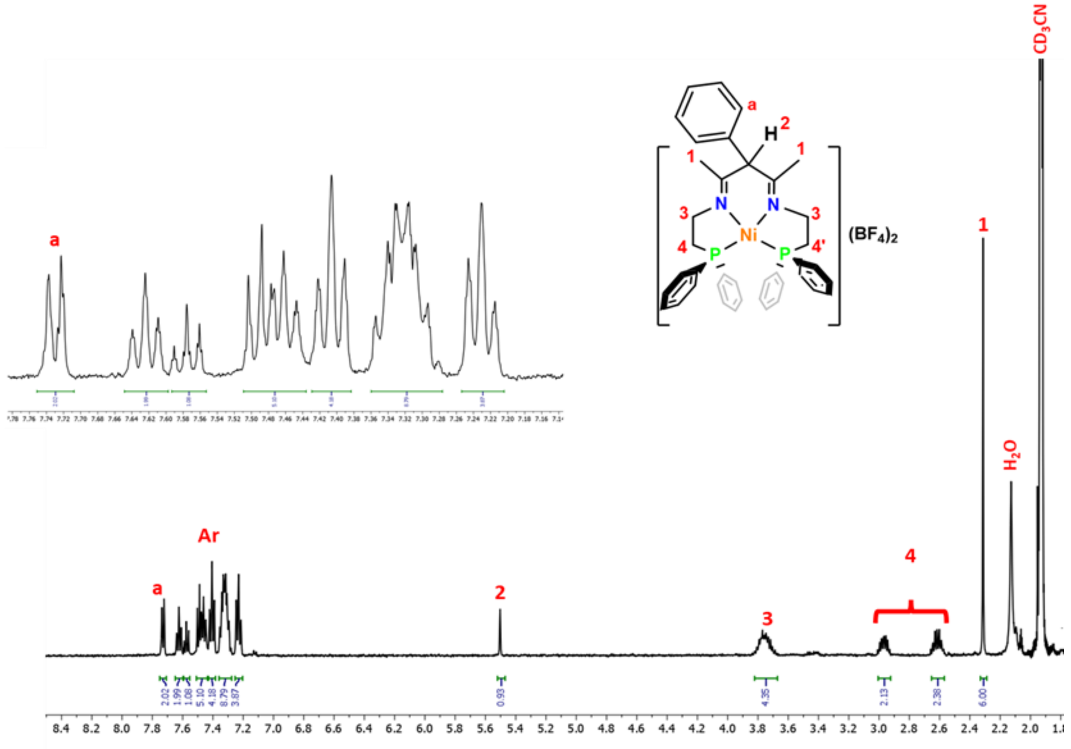

Figure S6. ^{1}H-NMR of complex **2(BF4)2** in CD_3CN

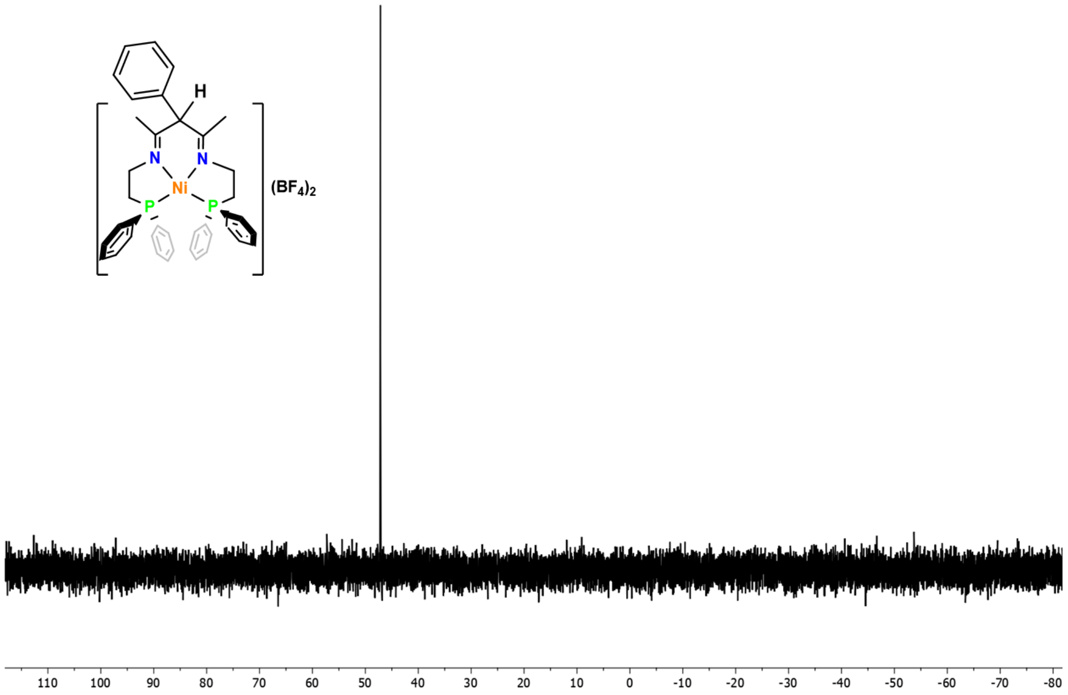

Figure S7. ^{31}P NMR of complex **2(BF$_4$)$_2$** in **(CD$_3$CN)**

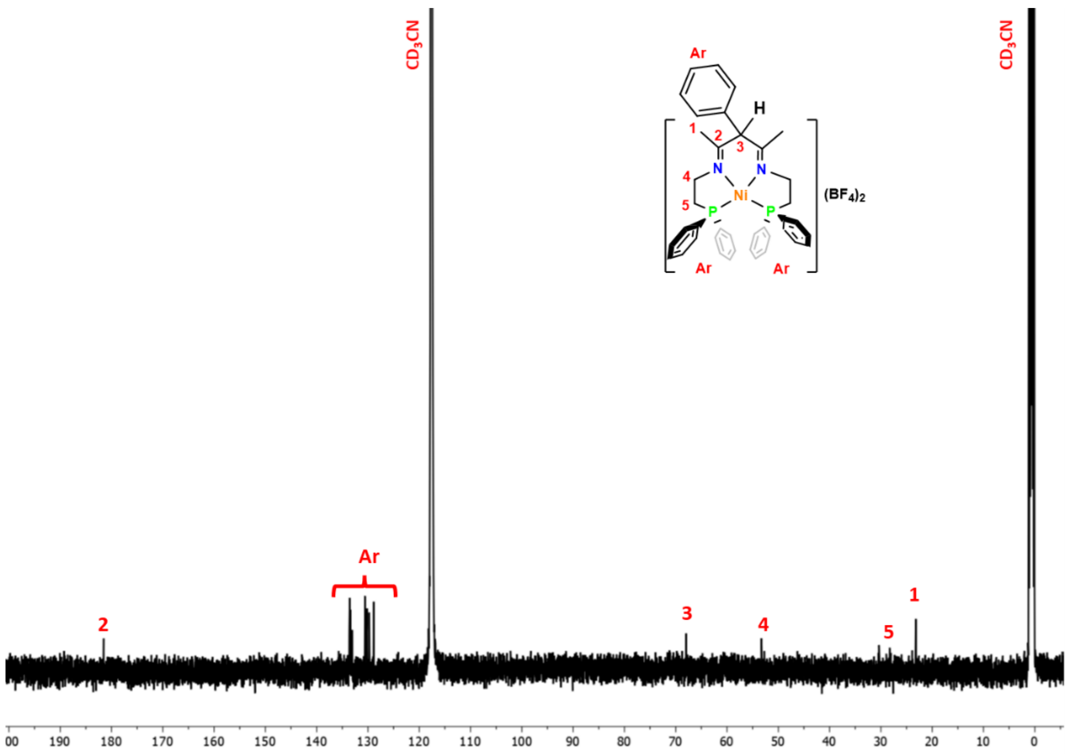

Figure S8. ^{13}C NMR of complex **2(BF$_4$)$_2$** in CD$_3$CN

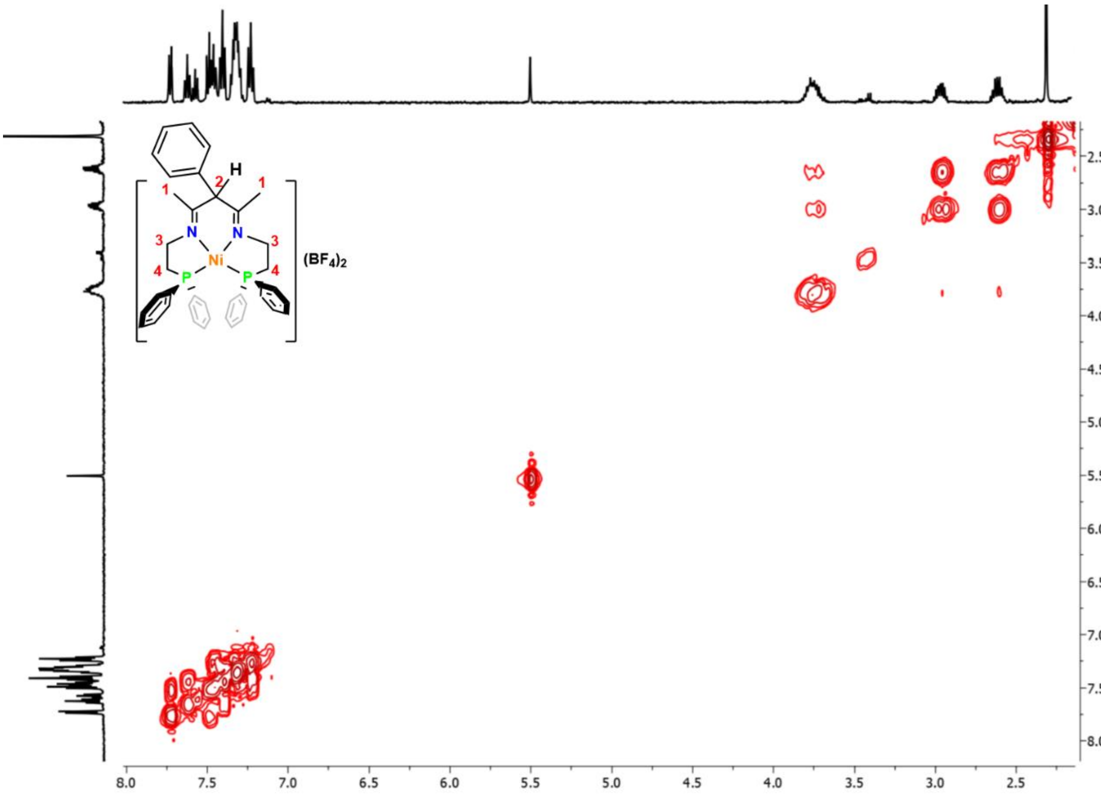

Figure S9. ^1H-^1H COSY NMR of **2(BF$_4$)$_2$** in CD_3CN

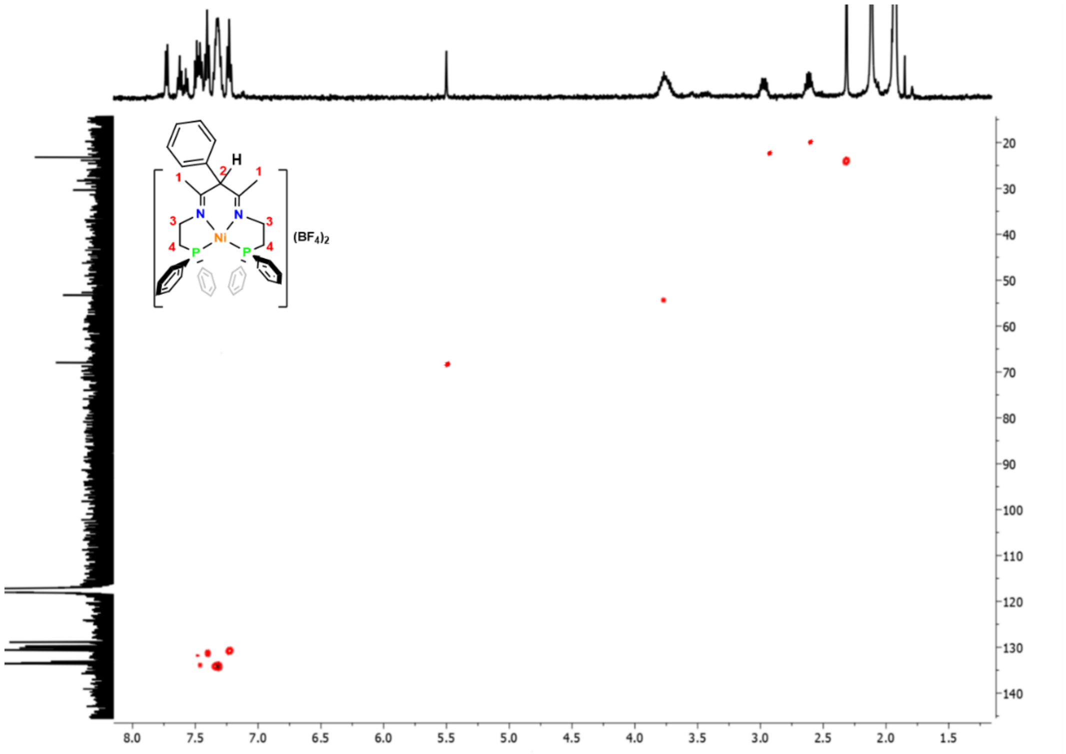

Figure S10. HSQC NMR of **1(BF$_4$)$_2$** complex in **(CD_3CN)**

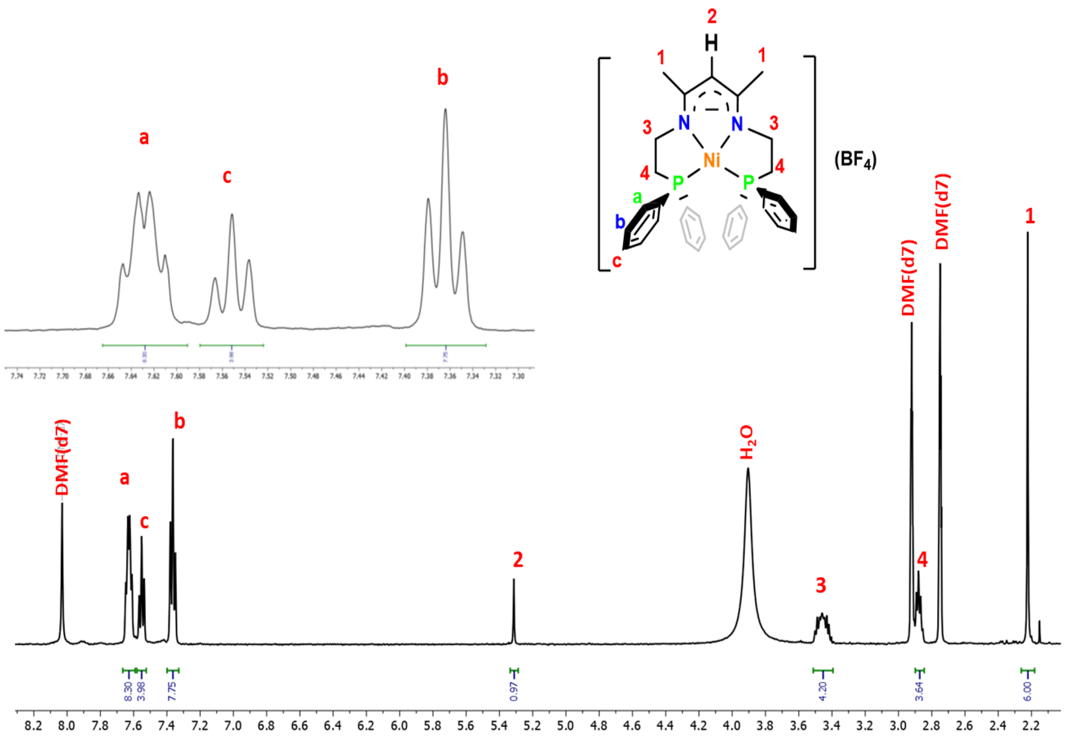

Figure S11. ^{1}H-NMR of complex **1(BF$_4$)$_2$** in **DMF(d$_7$)**

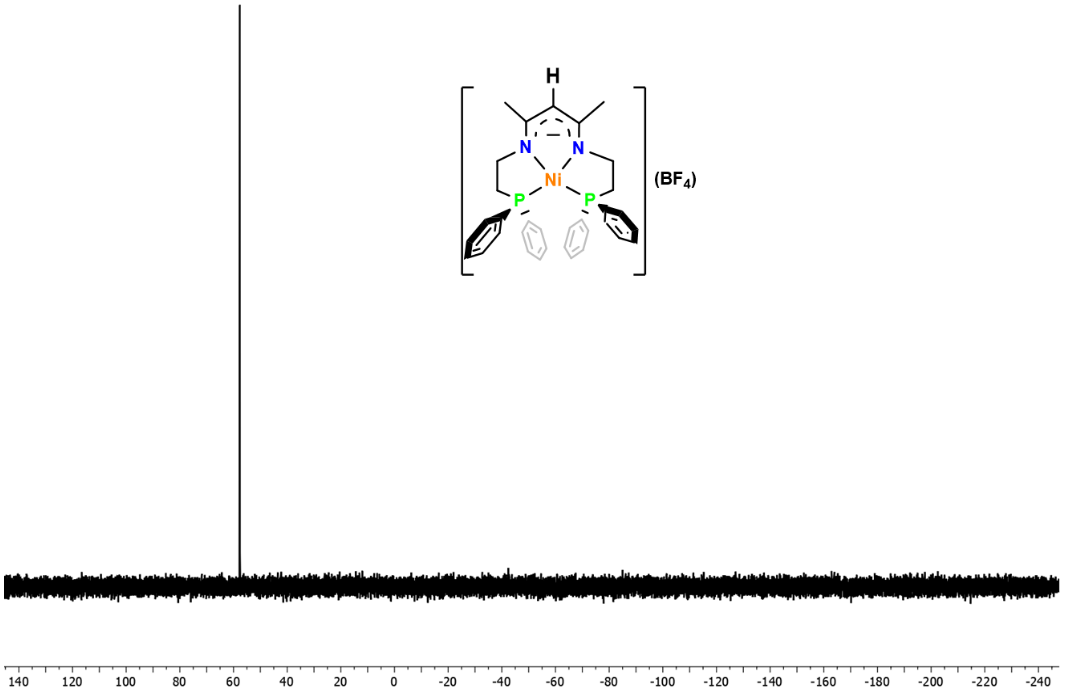

Figure S12. ^{31}P NMR complex **1(BF$_4$)$_2$** in **DMF (d$_7$)**

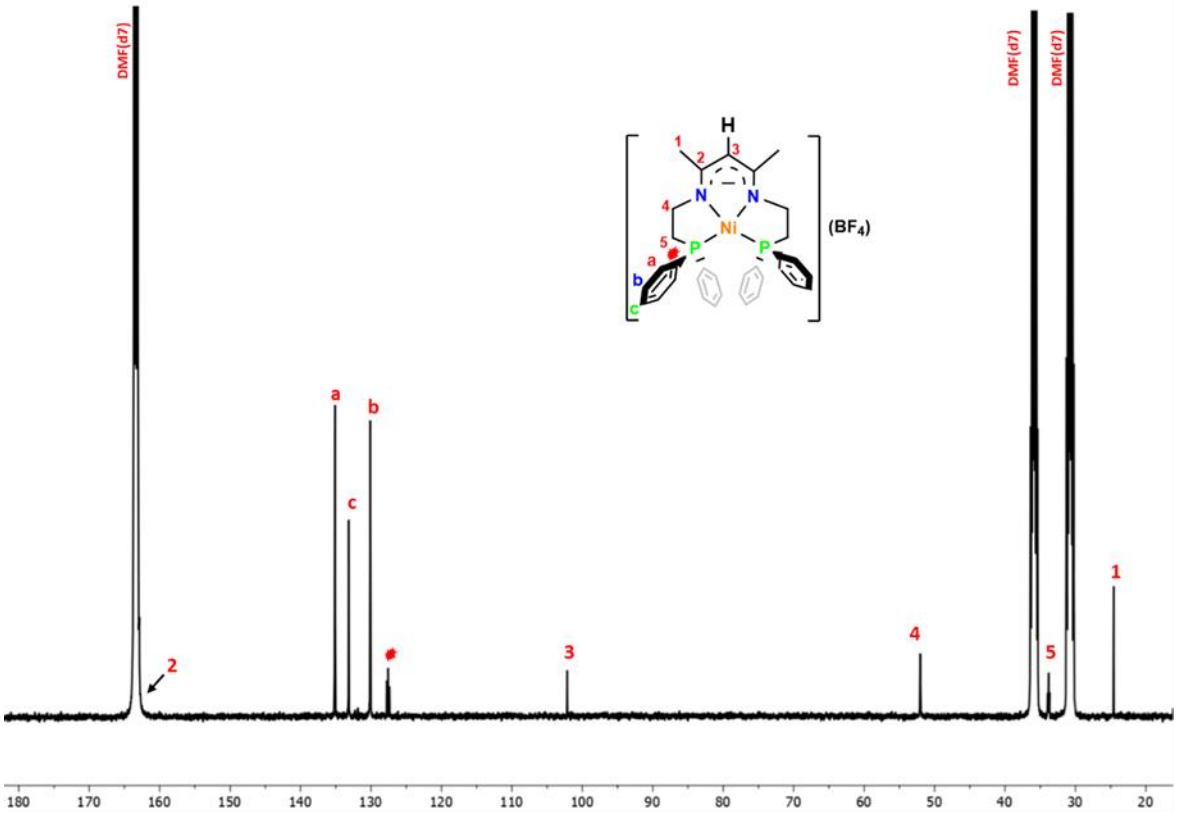

Figure S1. ^{13}C NMR of complex **1(BF$_4$)$_2$** in **DMF (d$_7$)**

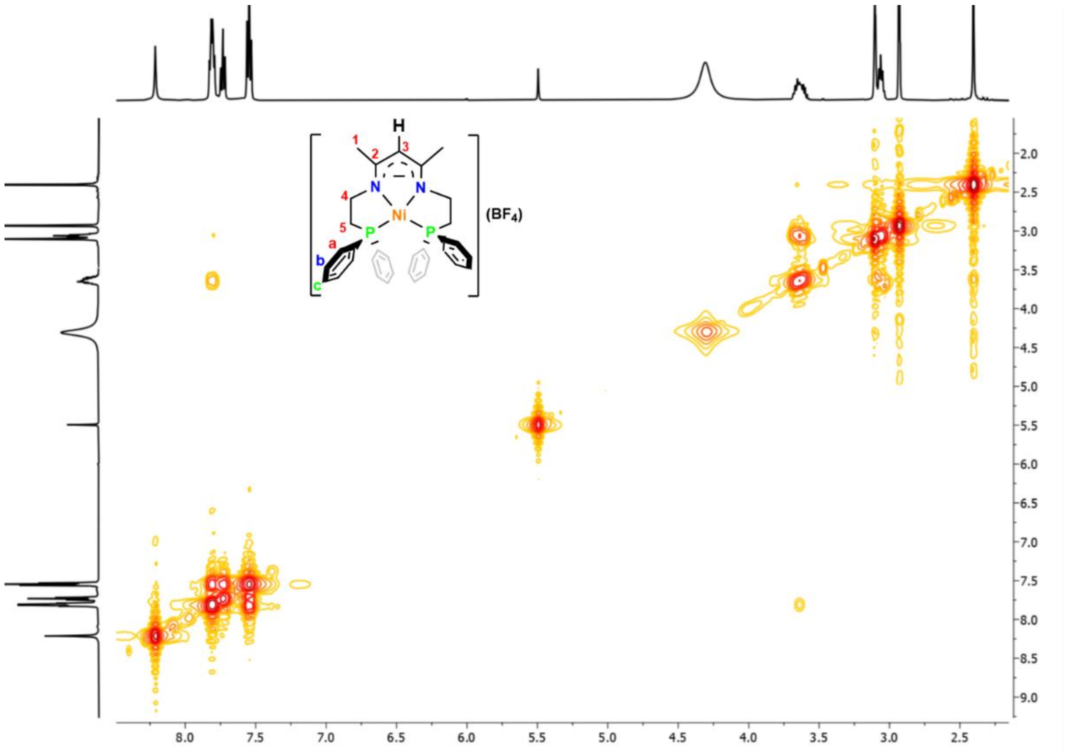

Figure S2. ^{1}H-^{1}H COSY NMR of **1(BF$_4$)$_2$** complex in **DMF (d$_7$)**

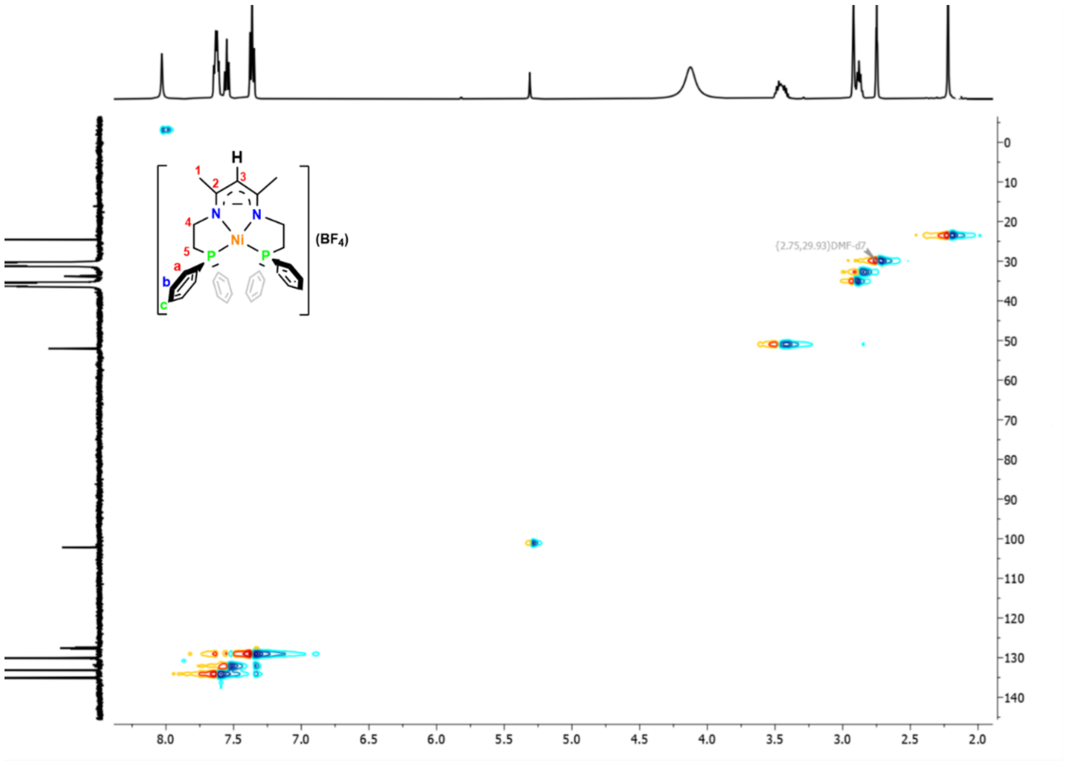

Figure S15- HSQC NMR of **1(BF$_4$)$_2$** complex in **DMF (d$_7$)**

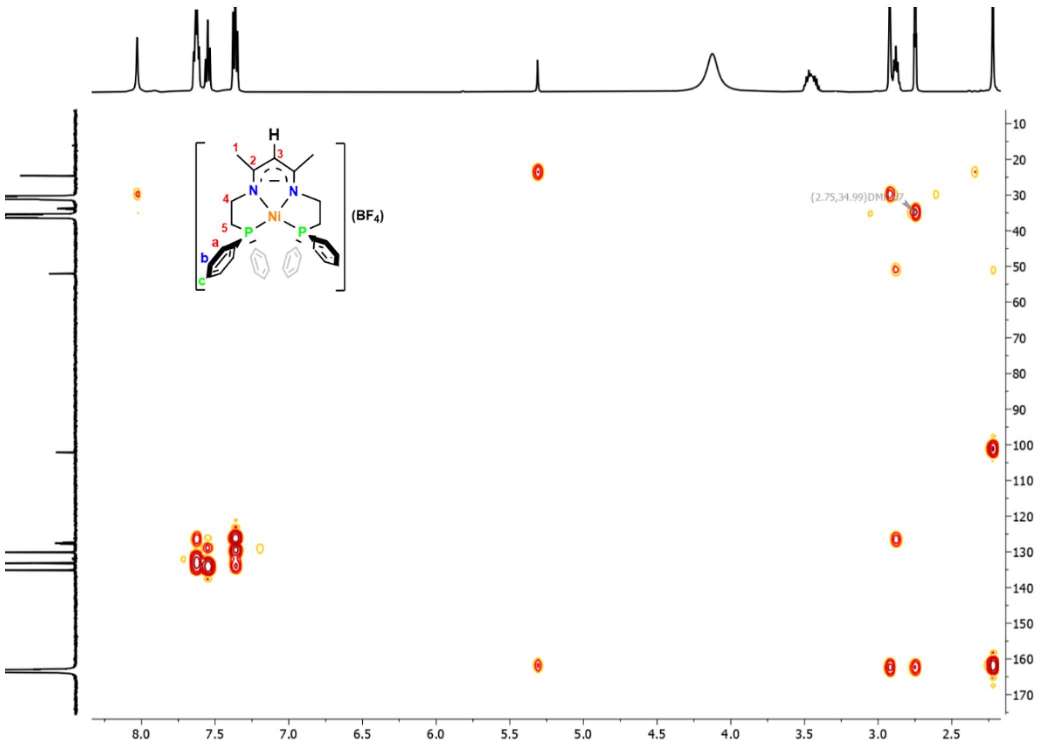

Figure S16- HMBC NMR of **1(BF$_4$)$_2$** complex in **DMF (d$_7$)**

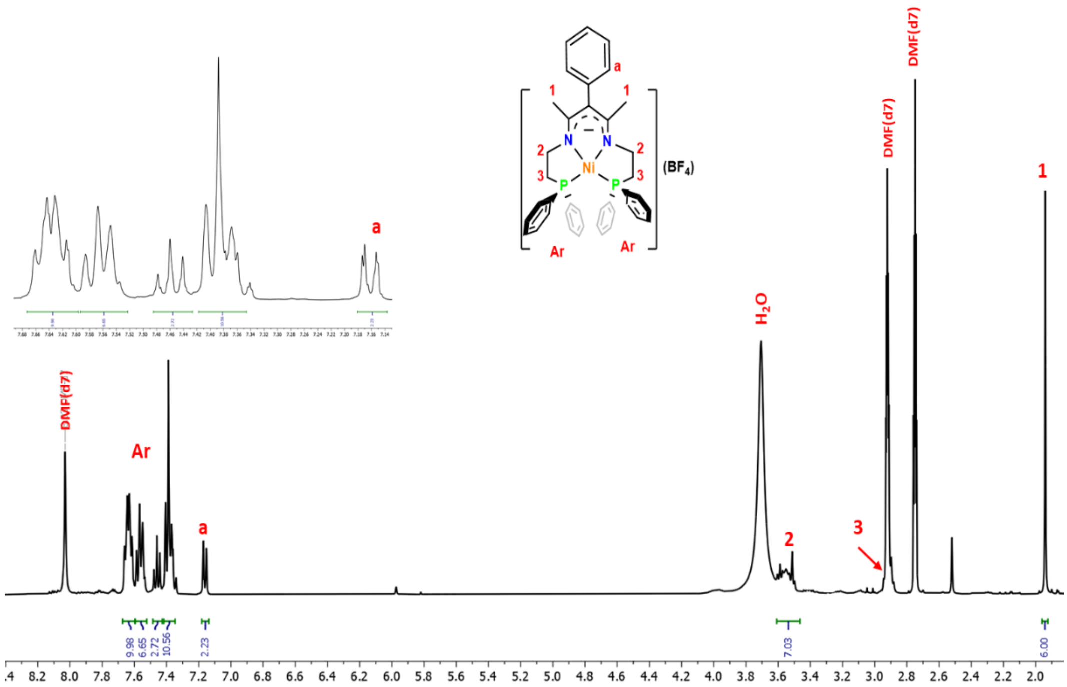

Figure S17-^{1}H-NMR of complex **$2(BF_4)_2$** in **DMF (d_7)**

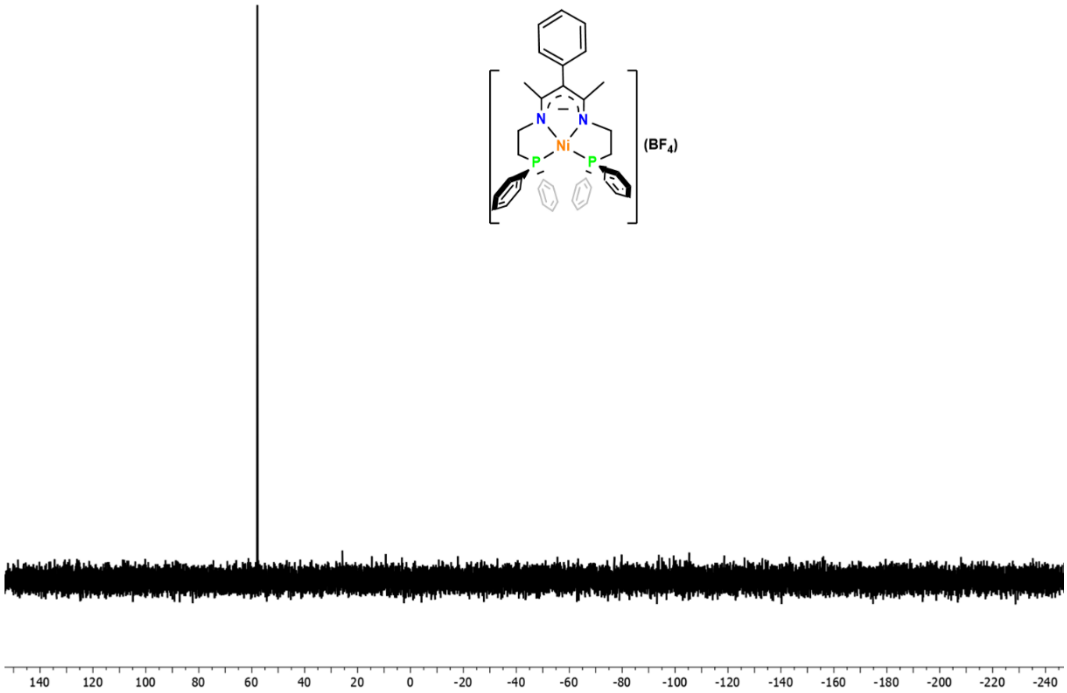

Figure S18-^{31}P NMR of complex **$2(BF_4)_2$** in **DMF (d_7)**

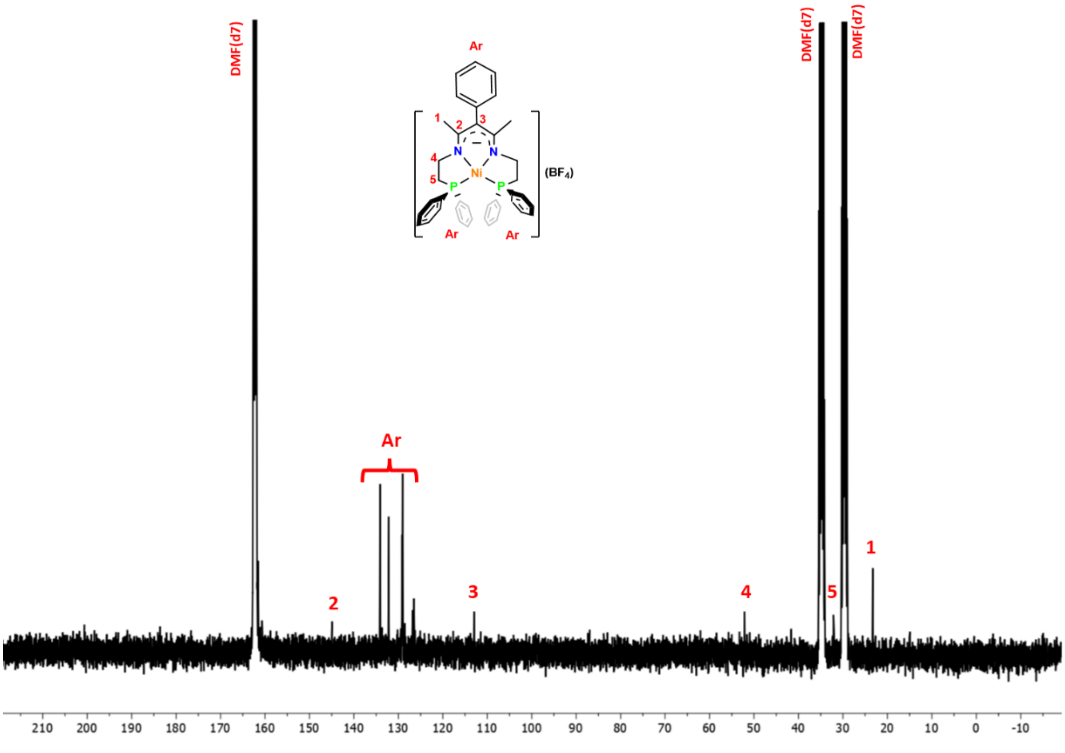

Figure S19-^{13}C NMR of complex **2(BF$_4$)$_2$** in **DMF (d$_7$)**

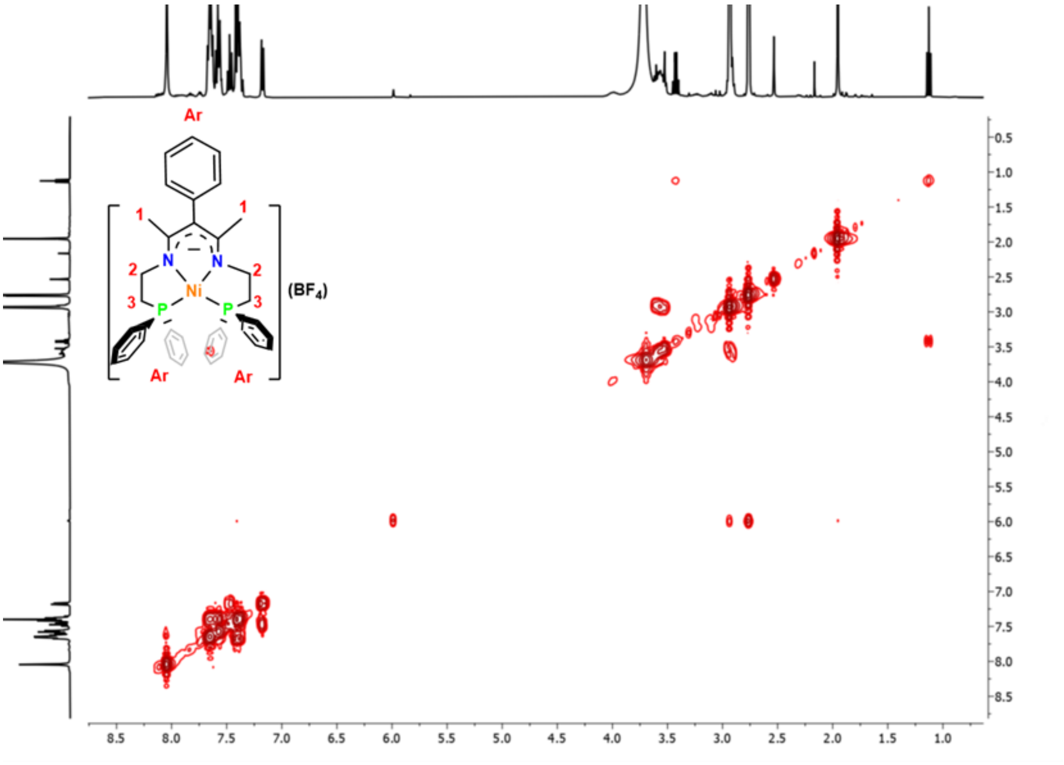

Figure S20-^{1}H-^{1}H COSY NMR of **2(BF$_4$)$_2$** in **DMF (d$_7$)**

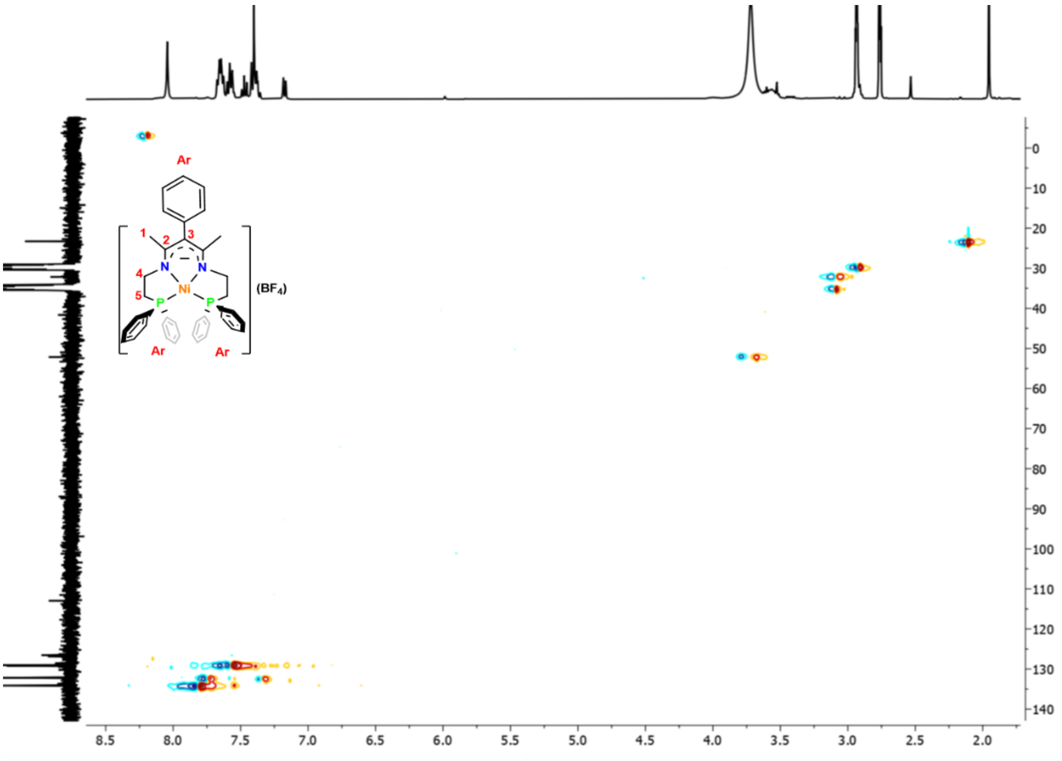

Figure S21- HSQC NMR of complex **2(BF$_4$)$_2$** in **DMF (d$_7$)**

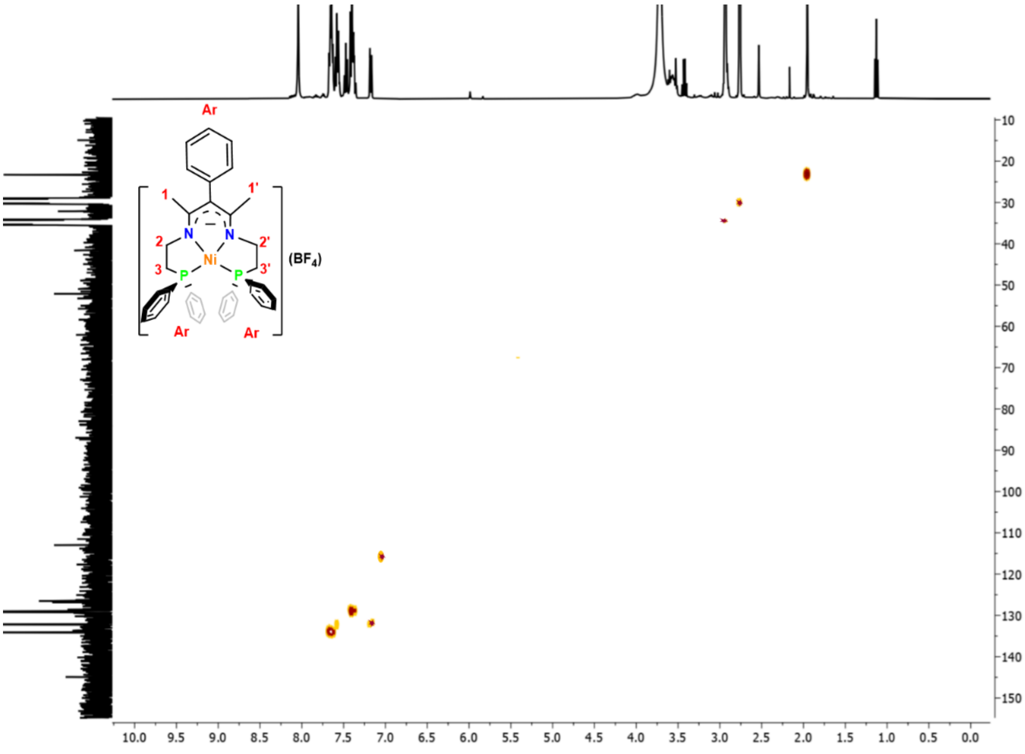

Figure S22- HMBC NMR of complex **2(BF$_4$)$_2$** in **DMF (d$_7$)**

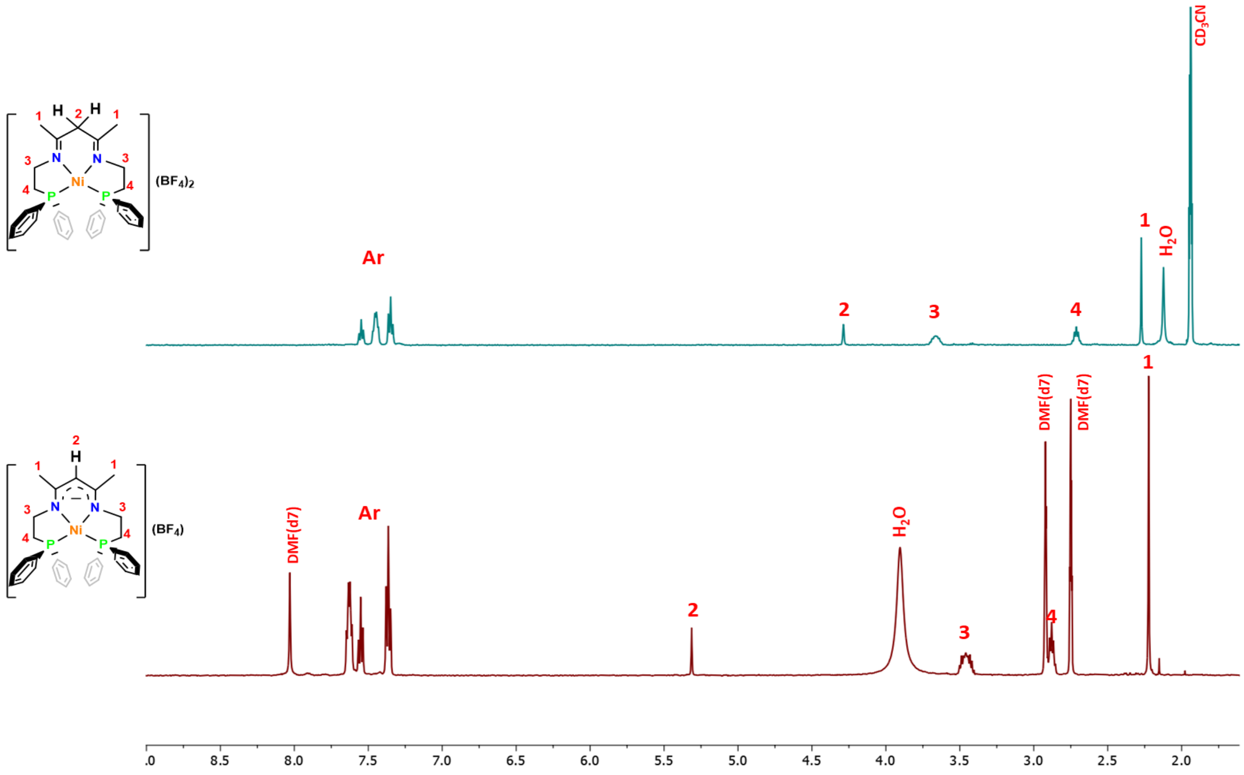

Figure S23- Comparison ^{1}H NMR spectra of Complex **1(BF4)2** in CD_3CN (**top**) and DMF (d_7) (**bottom**)

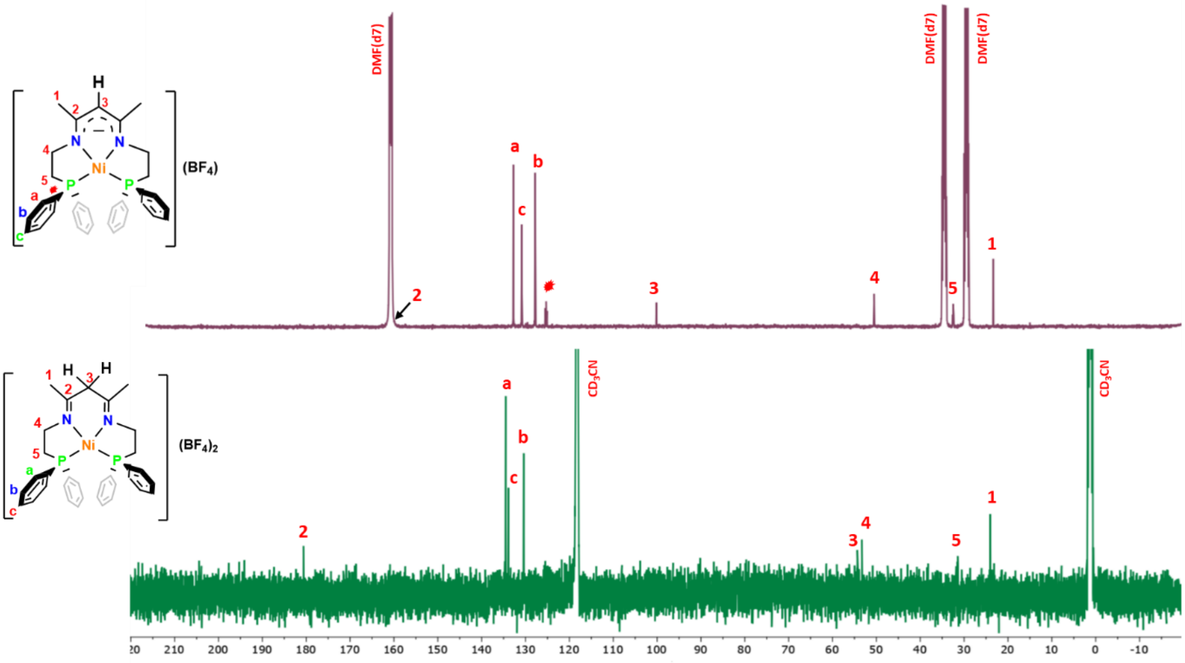

Figure S24- Comparison ^{13}C NMR spectra of Complex **1(BF4)2** in CD_3CN (**Bottom**) and DMF (d_7) (**top**)

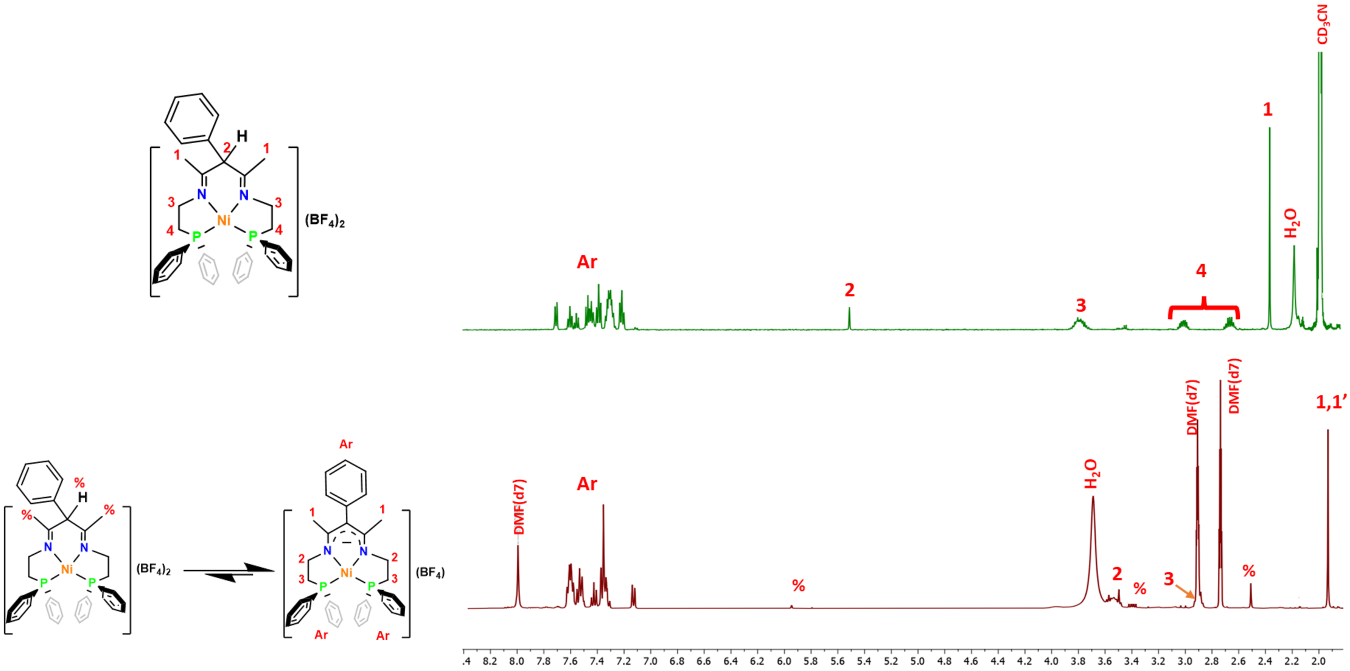

Figure S25- Comparison ^{1}H NMR spectra of Complex **2(BF4)2** in CD_3CN (**top**) and DMF (d_7) (**bottom**)

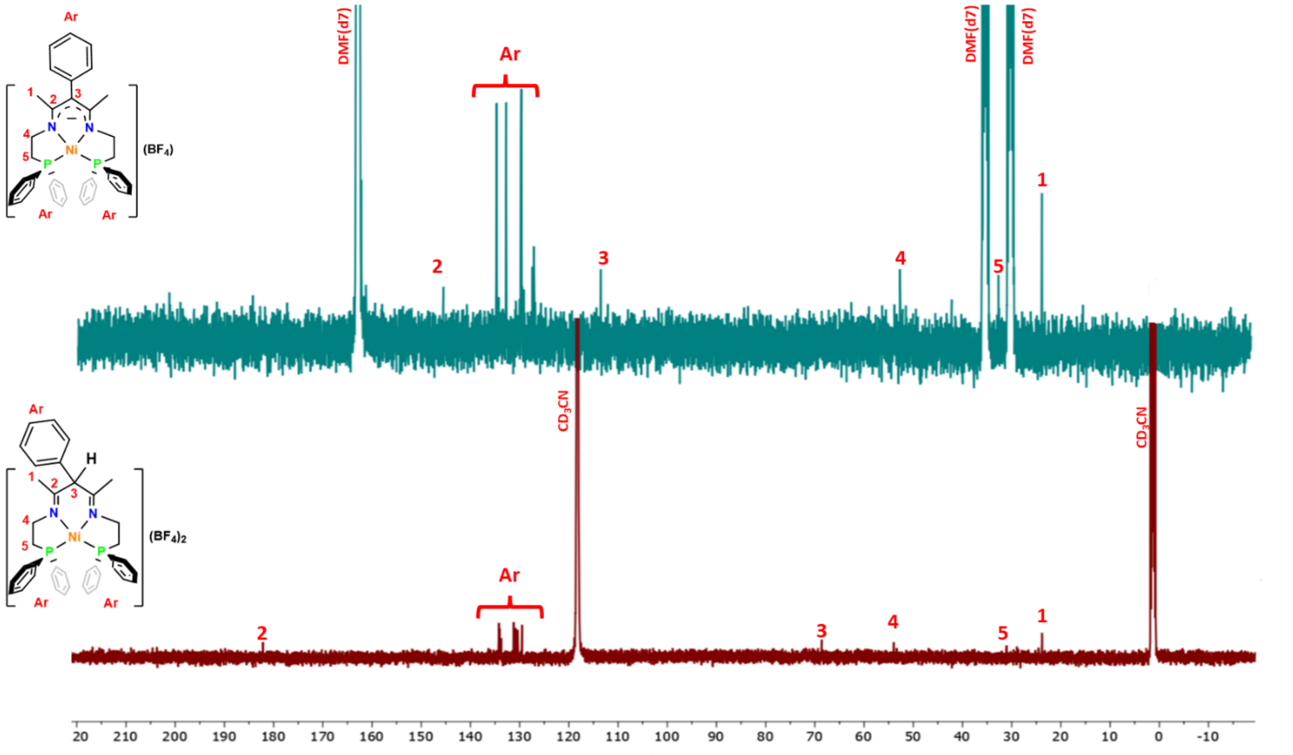

Figure S26- Comparison ^{13}C NMR spectra of Complex **2(BF4)2** in CD_3CN (**bottom**) and DMF (d_7) (**top**)

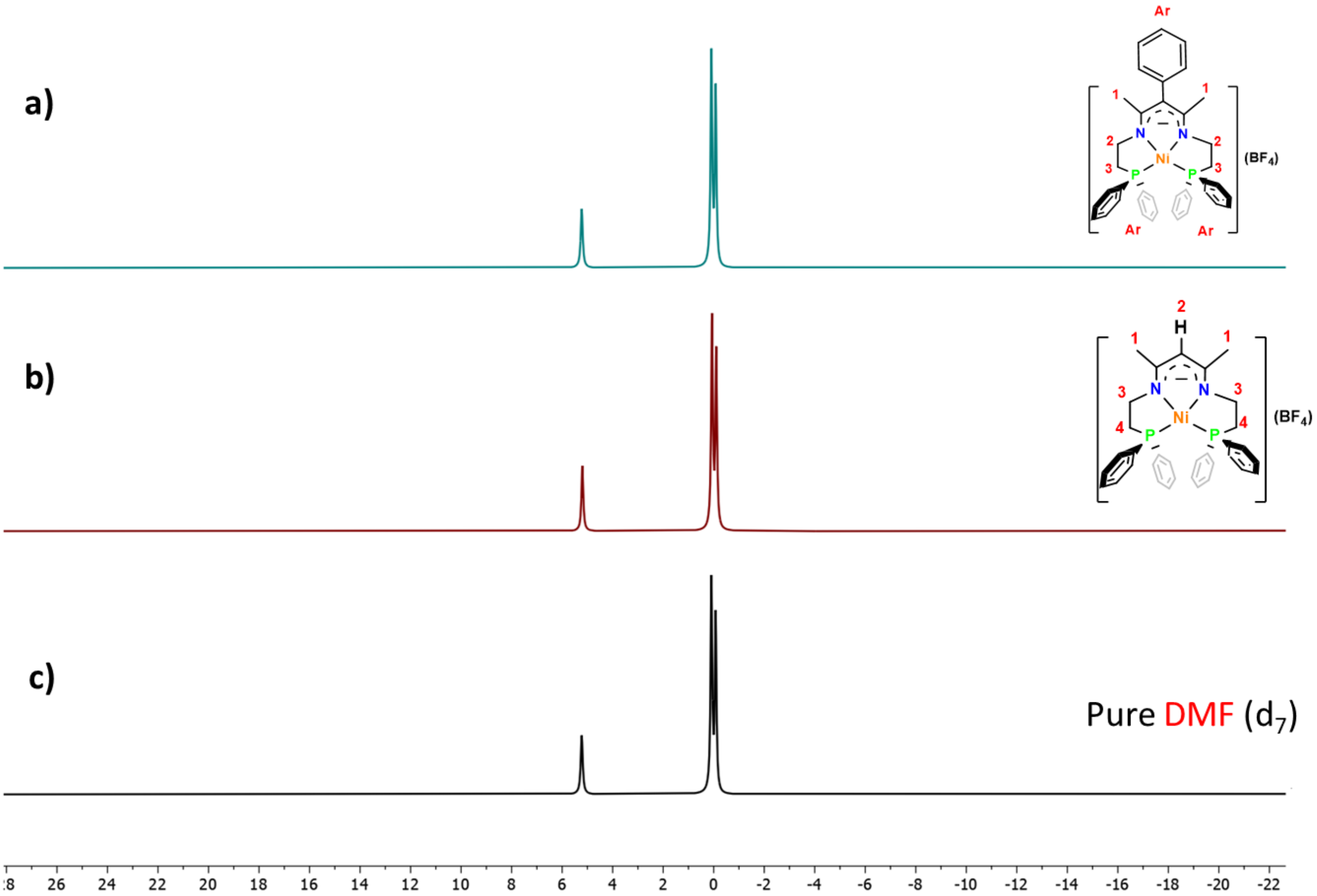

Figure S27- [2]D NMR of **2(BF$_4$)$_2$ (a), 1(BF$_4$)$_2$ (b)** in DMF (d$_7$). (**c**) [2]D NMR of pure DMF (d$_7$). Number of Scans=128 for each sample

3- Electrochemistry and Spectroelectrochemistry of 1^{2+} and 2^{2+}

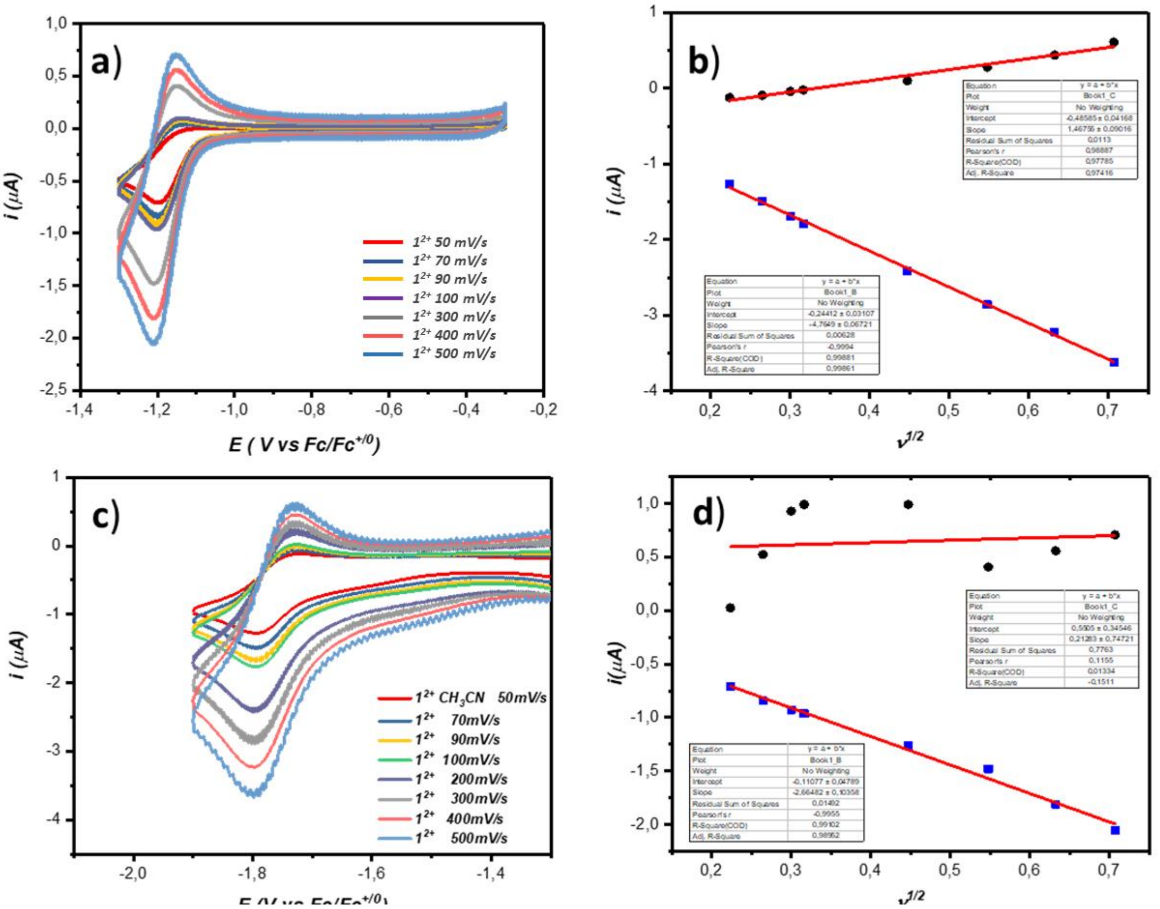

Figure S28. Cyclic voltammograms of 0.5 mM (**1^{2+}**) on first (**a**) and second (**b**) peak position in acetonitrile (**c,d**) Plots of (i vs $\upsilon^{1/2}$) for reduction and oxidation couples in presence of 0.1M $TBAPF_6$ as supporting electrolyte, **WE**: GC(1mm), **CE**: Pt, **REF** electrode: 0.01M $Ag/AgNO_3$

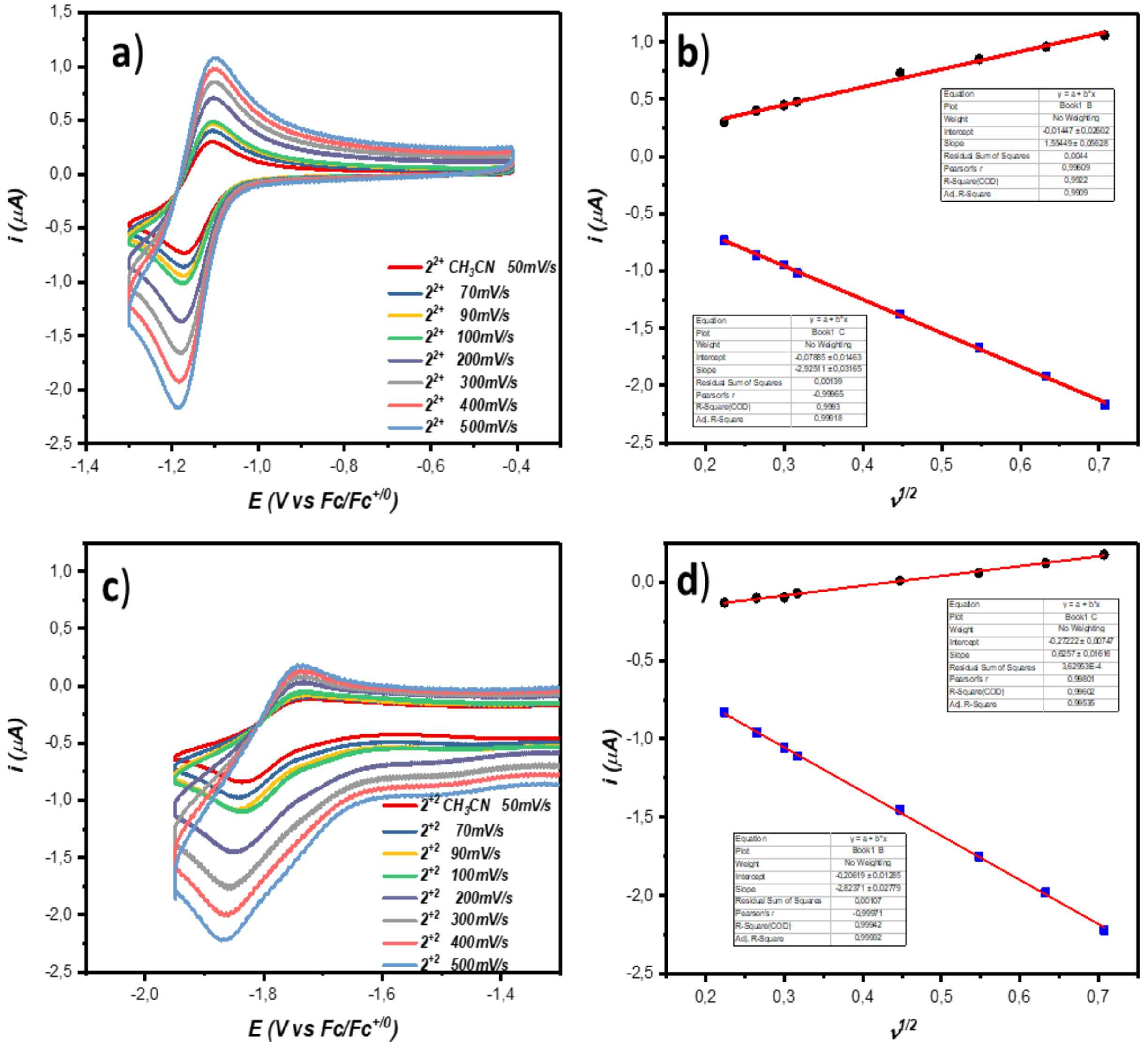

Figure S29- Cyclic voltammograms of 0.5 mM (**2**$^{2+}$) on first (**a**) and second peak position (**b**) in acetonitrile. (**c,d**) Plots of (i vs $\upsilon^{1/2}$) for reduction and oxidation couples in prescence of 0.1M $TBAPF_6$ as supporting electrolyte, **WE**: GC (1mm), **CE**: Pt, **REF** electrode: 0.01M $Ag/AgNO_3$

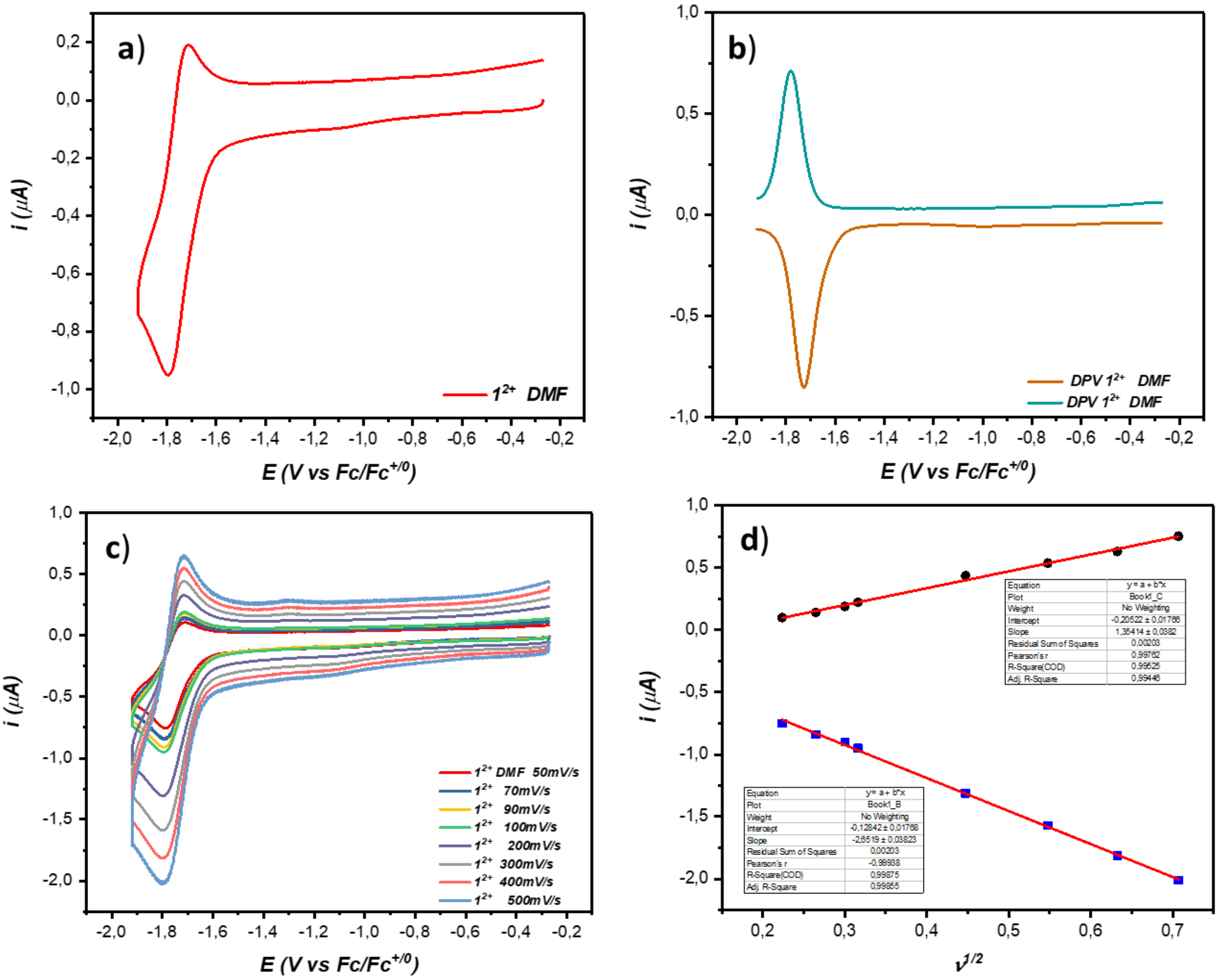

Figure S30. (**a**) cyclic voltammogram of ($\mathbf{1}^{2+}$) in DMF Scan rate 100mV/s. (**b**) DPV of ($\mathbf{1}^{2+}$) amplitude 50mV/s. (**c**) cyclic voltammogram of ($\mathbf{1}^{2+}$) in DMF with different Scan rates. (**d**) Plot of reduction and oxidation peak currents versus square root of Scan rates for ($\mathbf{1}^{2+}$) in DMF

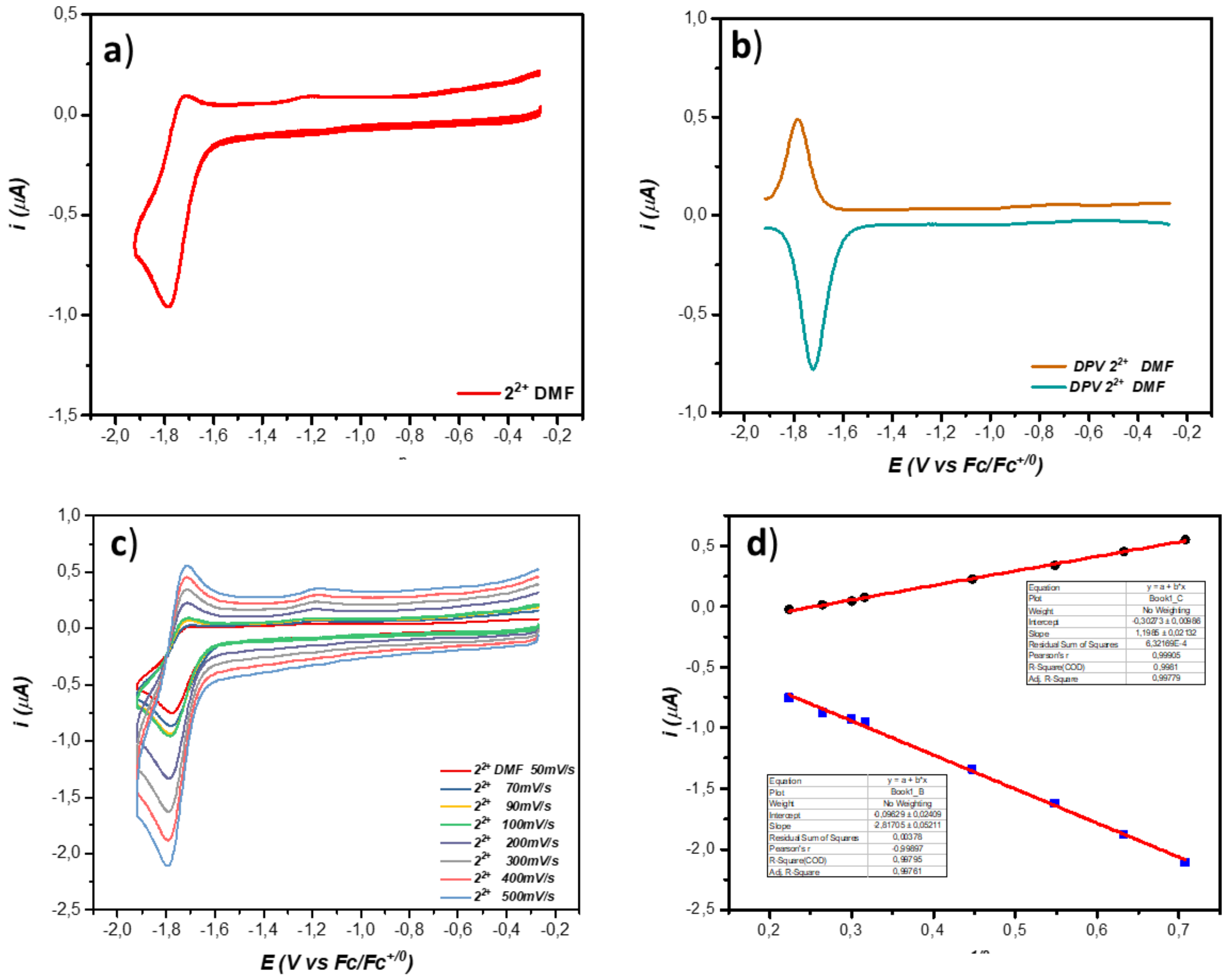

Figure S31. (**a**) cyclic voltammogram of (**2**$^{2+}$) in DMF Scan rate 100mV/s. (**b**) DPV of (**2**$^{2+}$) amplitude 50mV/s. (**c**) cyclic voltammograms of (**2**$^{2+}$) in DMF with different Scan rates. (**d**) Plots of reduction and oxidation peak currents versus square root of Scan rates (**2**$^{2+}$) in DMF

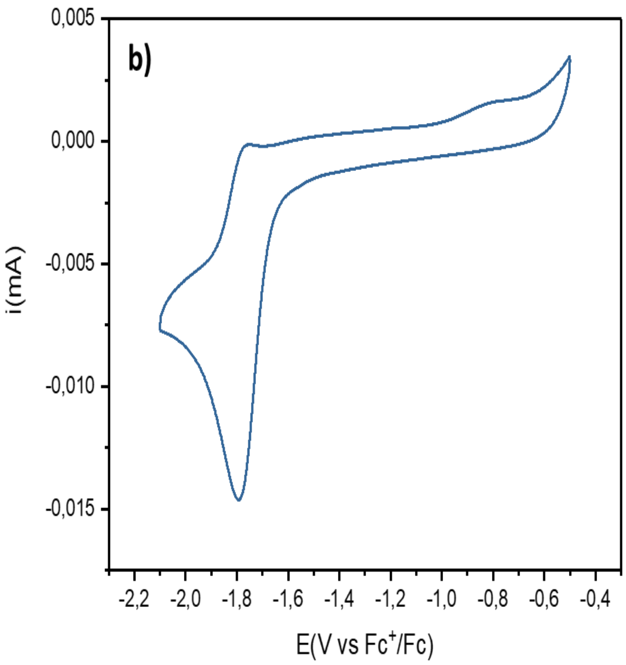

Figure S32. Cyclic voltammetry experiment of a 0.5 mM solution of **2**$^{2+}$ in acetonitrile in the prescence of 10eq triethylamine.

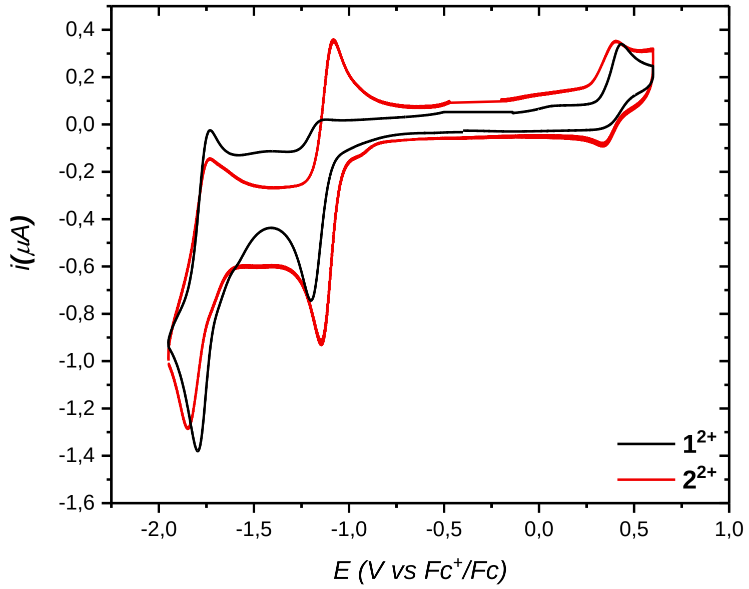

Figure S33. Cyclic voltammograms of 0.5 mM of **1**$^{2+}$ (Black) and **2**$^{2+}$ (red) in acetonitrile in presence of 0.1M $TBAPF_6$ as supporting electrolyte, **WE**: GC (1mm), **CE**: Pt, **REF** electrode: 0.01M $Ag/AgNO_3$. Full range.

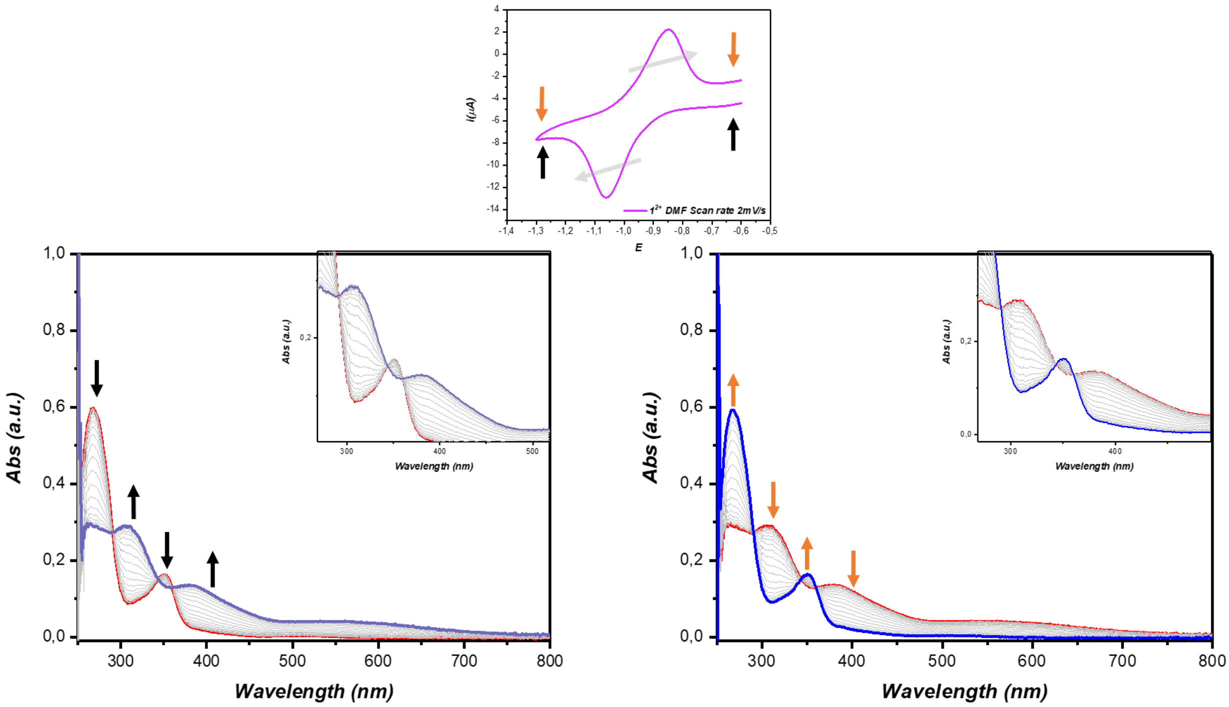

Figure S34. Cyclic voltammetry experiment in the OTTLE cell (WE = Platinum mesh; CE = Platinum wire; RE: Silver pseudo-reference electrode; solvent = dimethylformamide-0.1 M [Bu_4N] PF_6; [$\mathbf{1}^{2+}$] = 2 mM; scan rate = 2 mV/s. Black, and orange arrows show the starting/ending of the UV-vis spectra acquisition. Grey arrows indicate the polarity of the scan.

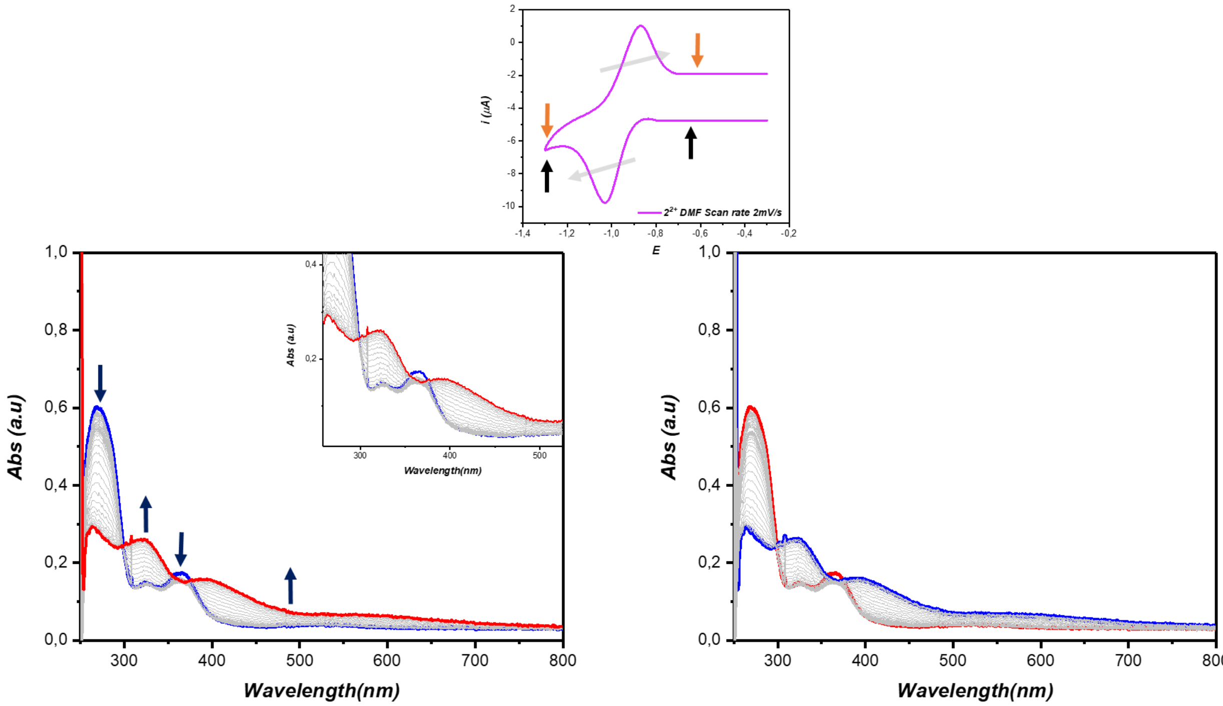

Figure S35- Cyclic voltammetry experiment in the OTTLE cell (WE = Platinum mesh; CE = Platinum wire; RE: Silver pseudo-reference electrode; solvent = dimethylformamide-0.1 M [Bu_4N] PF_6; [$\mathbf{2}^{2+}$] = 2 mM; scan rate = 2 mV/s. Black, and orange arrows show the starting/ending of the UV-vis spectra acquisition. Grey arrows indicate the polarity of the scan

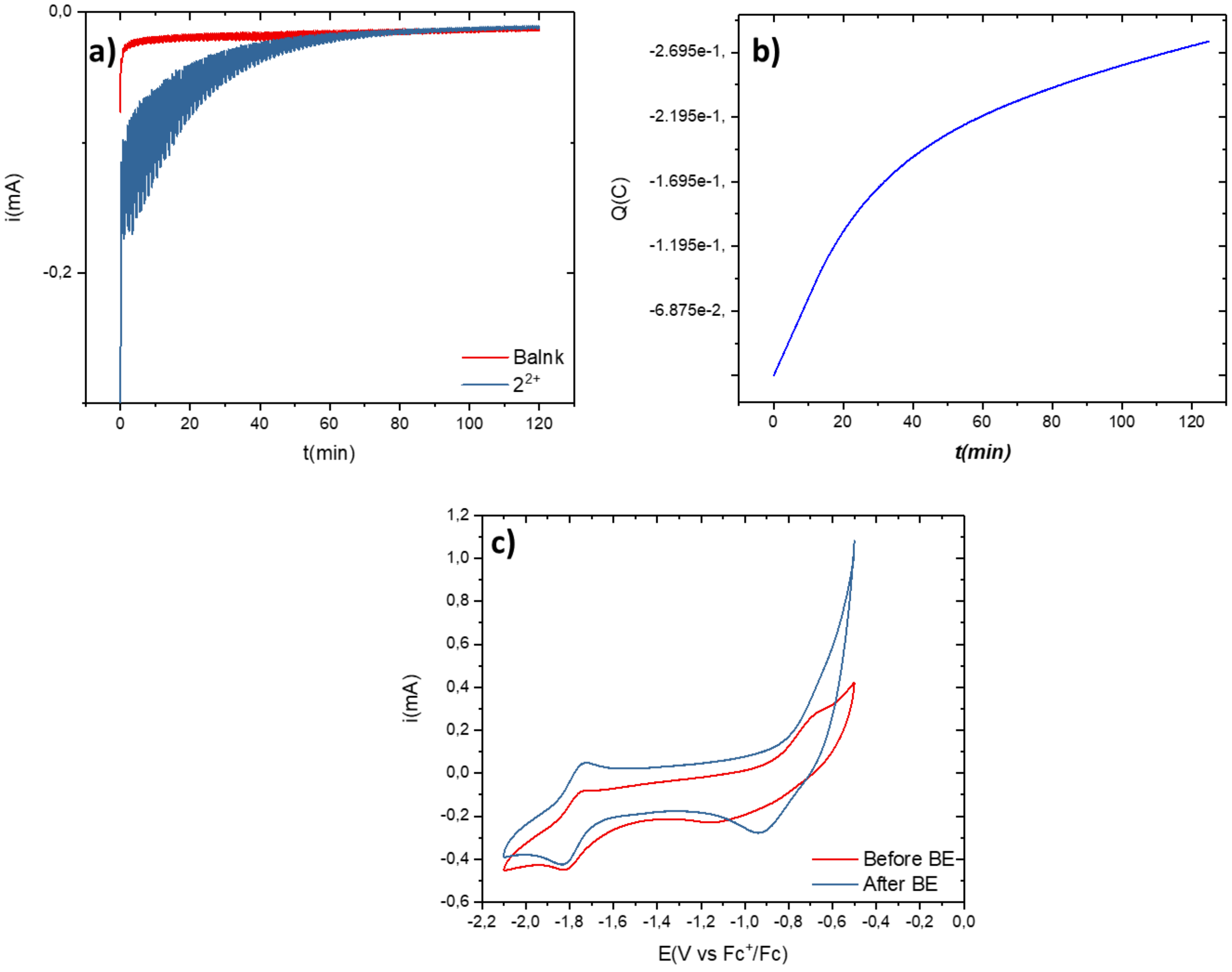

Figure S36- a) Current versus time plot of a bulk electrolysis experiments of a 0.5 mM solution of $\mathbf{2}^{2+}$ in acetonitrile (5mL, 0,1M $TBAPF_6$) at E_{app}= -1.2V vs $Fc/Fc^{+/0}$ in a two-compartment cell with of Pt foil as a counter electrode, GC plate (S = 1 cm^2) as working electrode, and Ag/Ag^+ reference electrode (0.01 $AgNO_3$, 0.1 $TBAPF_6$). **b**) Charge passed through the working electrode during the bulk electrolysis in a). **c**) cyclic voltammetry of $\mathbf{2}^{2+}$ before and after the bulk electrolysis experiment in a).

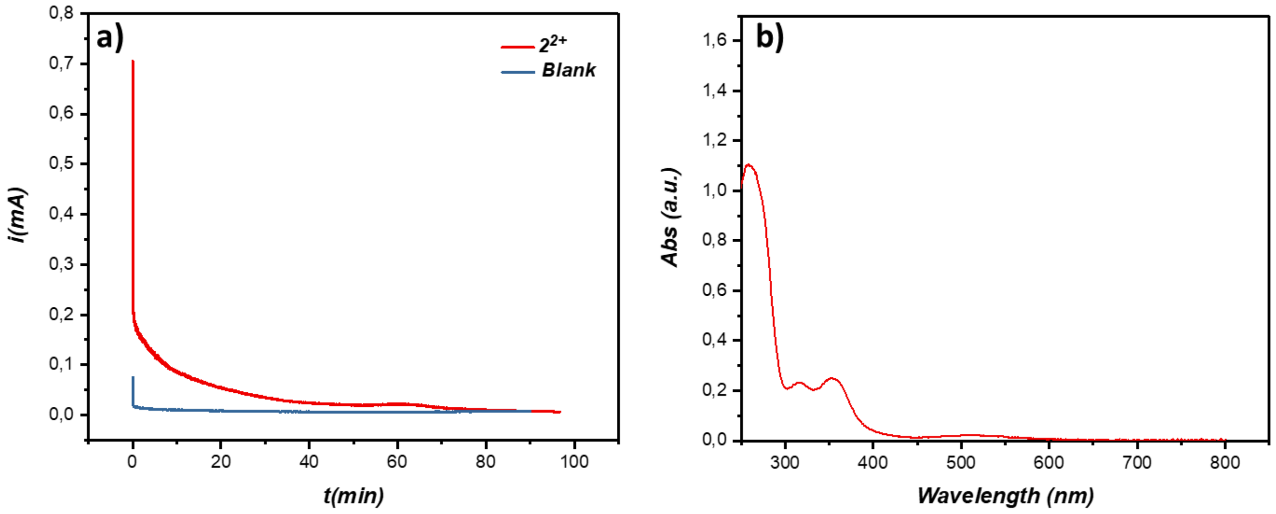

Figure S37- a) Bulk electrolysis experiments of a 0.4 mM solution of $\mathbf{2^{2+}}$ in acetonitrile (5ml, 0,1M $TBAPF_6$) at E_{app}= -1.3V vs $Fc/Fc^{+/0}$ in a two-compartment cell with of Pt mesh as a counter Electrode, GC rod (S = 3,1 cm^2) as working electrode, and Ag/Ag^+ reference electrode (0.01 $AgNO_3$, 0.1 $TBAPF_6$). **a**) Charge passed through the working electrode during bulk electrolysis after subtraction of the background charge gives (Mols of electron/Mols of initial complex= 1 at -1.3V). **b**) UV-vis spectra of $\mathbf{2^{2+}}$ after the bulk electrolysis experiment in CH_3CN

4- UV-Vis Spectroscopy of 1^{2+} and 2^{2+}

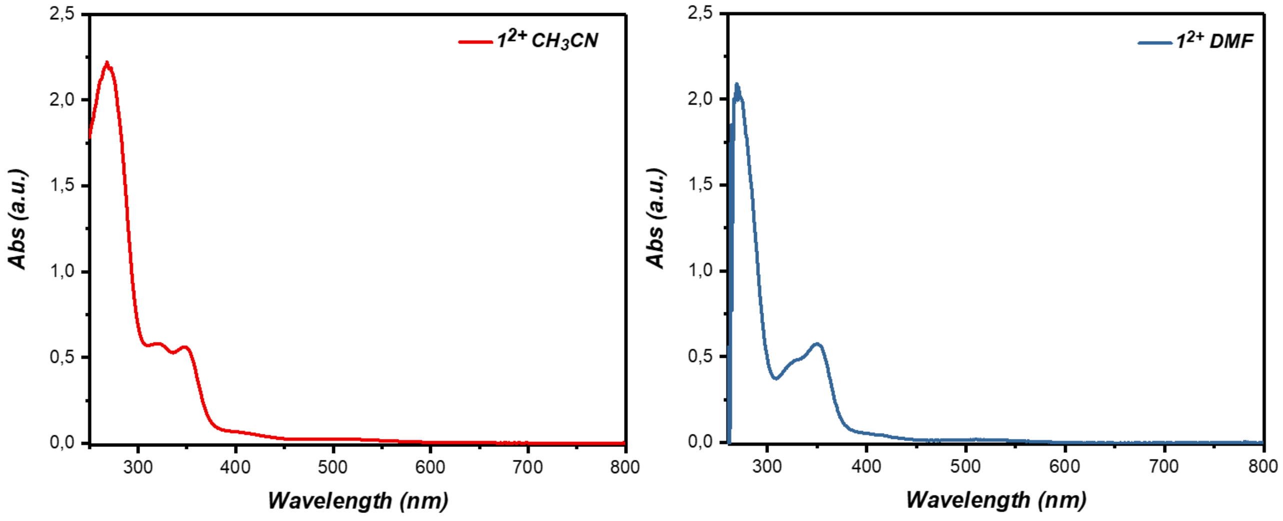

Figure S38- UV-visible spectrum of 0.125mM of $\mathbf{1}^{2+}$ in CH_3CN and DMF. See main manuscript for band position and extinction coefficients.

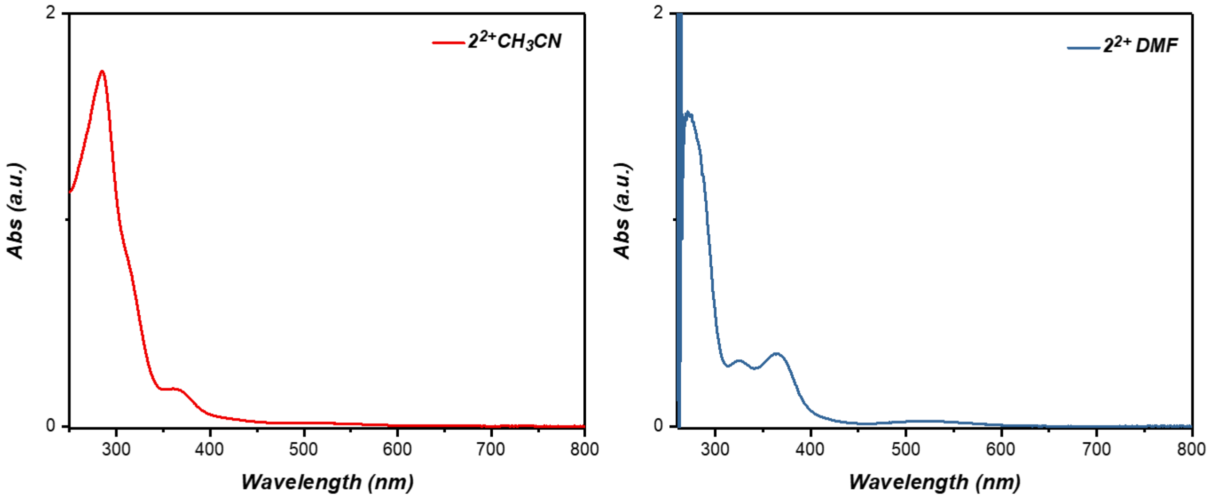

Figure S39- UV visible spectrum of 0.125mM of $\mathbf{2}^{2+}$ in CH_3CN and DMF.

5- Electrochemistry and gas detection experiments in the presence of organic acids

a) b)

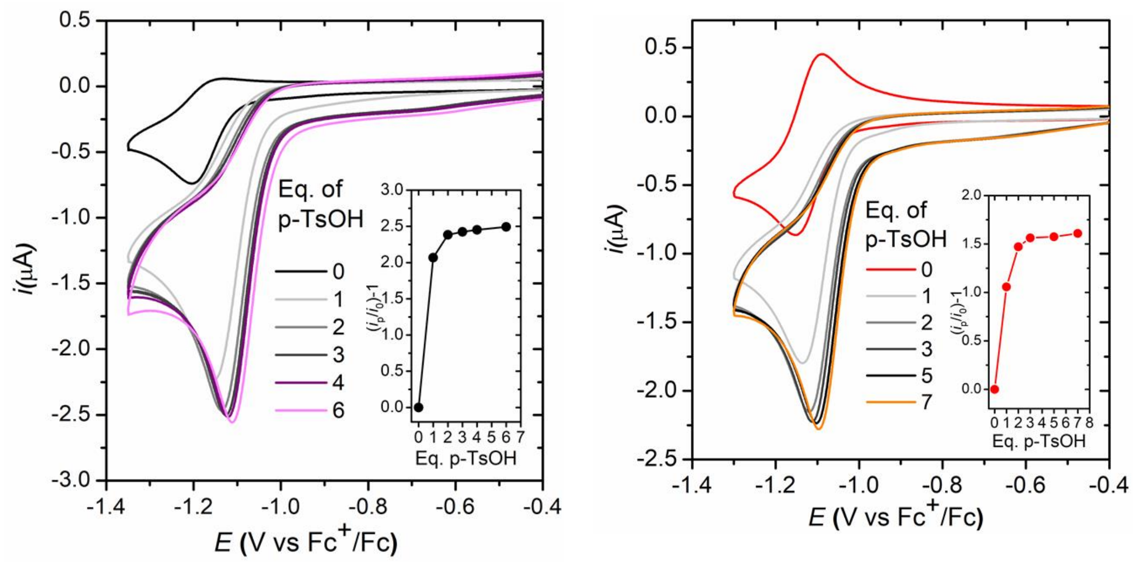

Figure S40: Cyclic voltammetry experiments of **1**$^{2+}$(**a**) and **2**$^{2+}$ (**b**) in CH_3CN solution with successive addition of p-TsOH·H_2O at scan rate 0.1V/s. The inset figures show the growth of peak intensity (i_p) relative to the peak in the absence of acid (i_0).

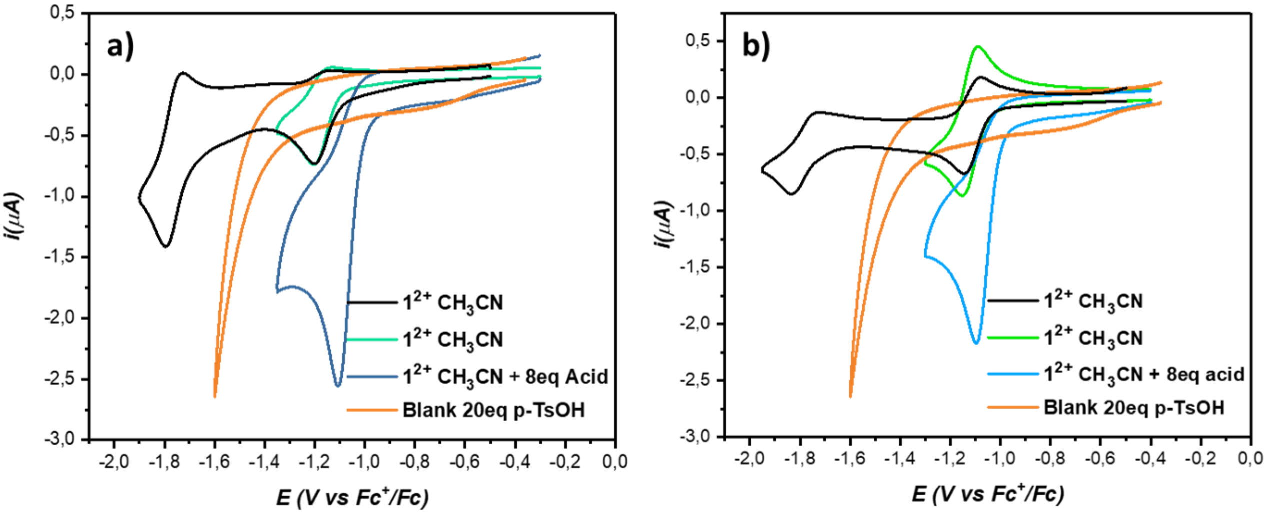

Figure S41-Voltammograms of 0.5 mM **1**$^{2+}$ (**a**) and **2**$^{2+}$ (**b**) in CH_3CN in absence of proton source (Black and green) and in prescence of 8eq p-TsOH in first reduction peak position (blue). Cyclic voltammogram of the blank in prescence of 20eq p-TsOH indicates proton reduction current on working electrode occurs between first and second reduction peak of the complexes (Orange)

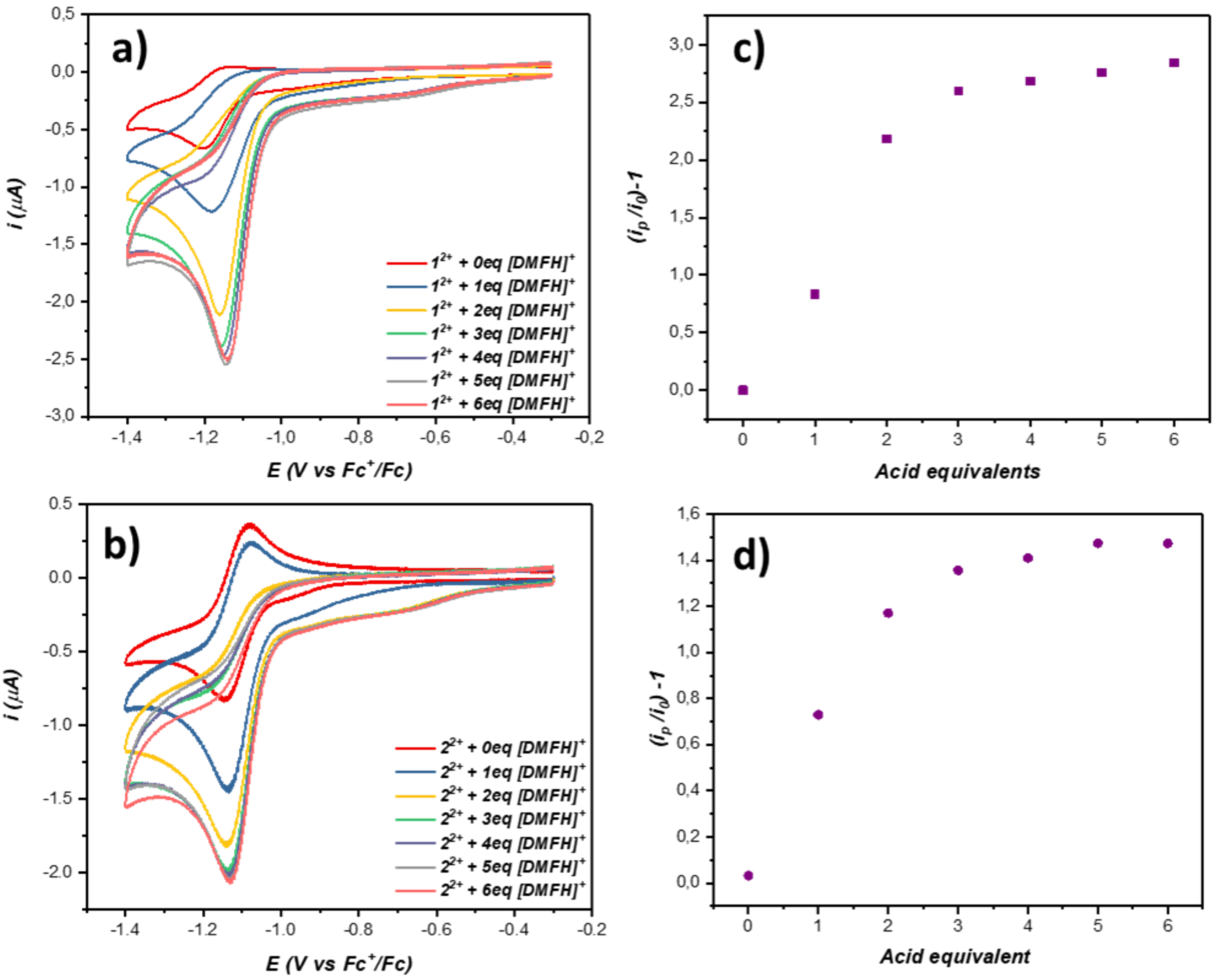

Figure S42. Voltammograms of 0.5 mM **1**$^{2+}$ (**a**) and **2**$^{2+}$ (**b**) with successive additions of [[DMFH]$^{+}$(CF_3O_3)$^{-}$] at Scan rate 0.1V/s in the presence of 0.1 M $TBAPF_6$ as supporting electrolyte in CH_3CN solution. (**c**) and (**d**) show the growth of peak intensity (i_p) relative to the peak in the absence of acid (i_0).

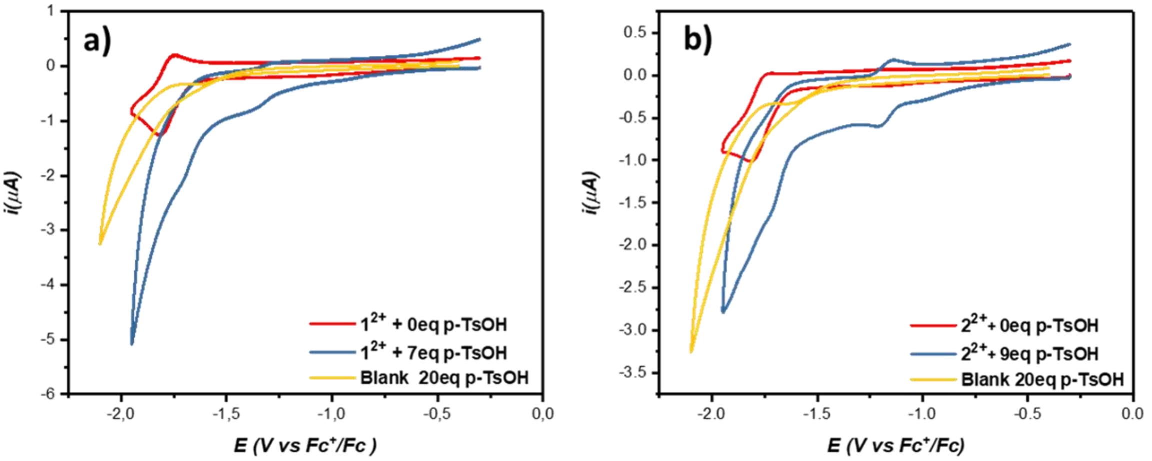

Figure S43- Cyclic Voltammograms of 0.5 mM **1**$^{2+}$ (**a**) and **2**$^{2+}$ (**b**) in the absence of proton source (red) and in prescence of 7eq p-TsOH and 9eq p-TsOH in DMF (blue). Cyclic voltammogram of the blank in prescence of 20eq p-TsOH in DMF (yellow).

a) b)

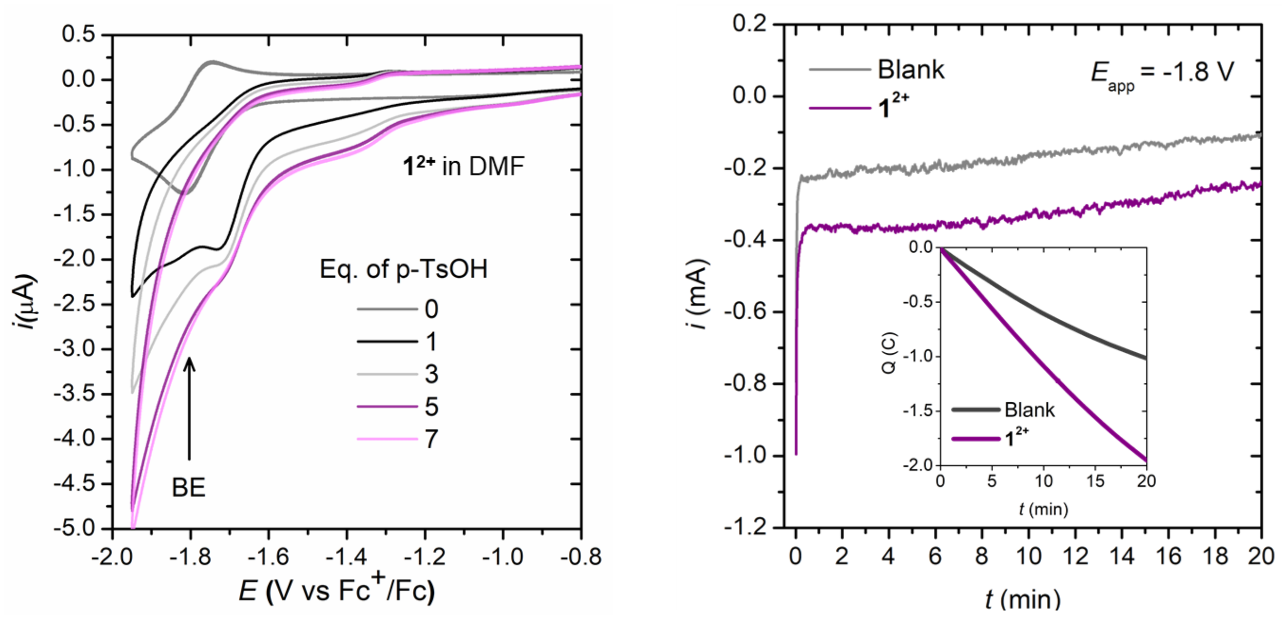

Figure S44. (**a**) Cyclic voltammetry experiments of a 0.5 mM solution of **1**$^{2+}$ in DMF (0.1 M $TBAPF_6$) after consecutive addition of p-TsOH·H_2O at scan rate 0.1V/s; **WE**: GC (Φ = 1mm), **CE**: Pt, **REF** electrode: Ag/$AgNO_3$ (Ag wire in a 0.01 M solution of $AgNO_3$ and 0.1 M $TBAPF_6$) . (**b**) Bulk electrolysis experiments at E_{app} = -1.8V vs Fc/$Fc^{+/0}$ in the presence of 60 eq. p-TsOH·H_2O in the presence (purple) and in the absence (grey) of a 0.5 mM solution of **1**$^{2+}$ in DMF (0.1 M $TBAPF_6$); **WE**; GC rod (S = 3.1 cm^2), **CE**: Pt mesh, **REF** electrode: Ag/$AgNO_3$ (Ag wire in a 0.01 M solution of $AgNO_3$ and 0,1 M $TBAPF_6$), $V_{cathode}$ = V_{anode} = 5 mL.

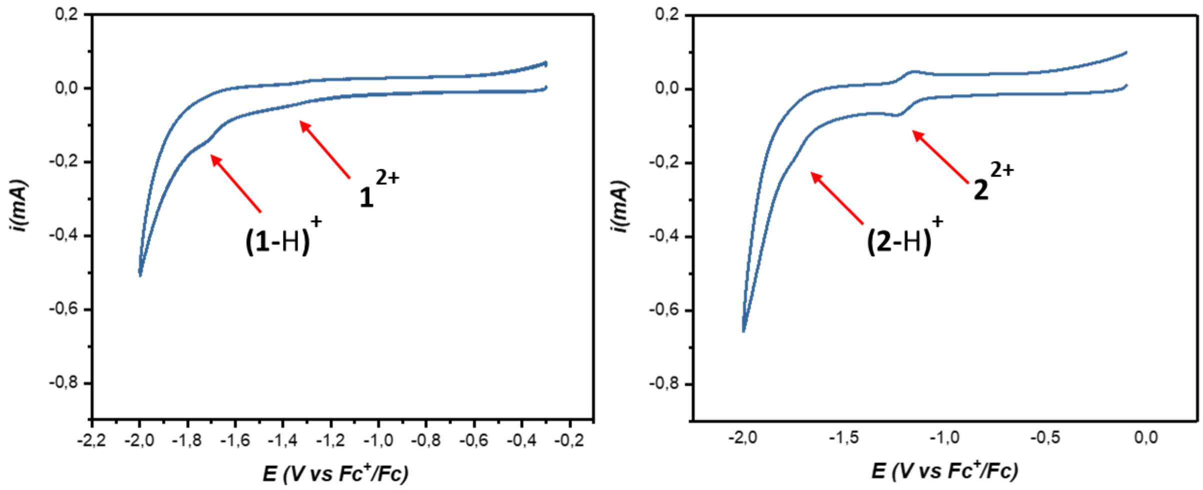

Figure S45- Cyclic Voltammograms of a solution of 0.5 mM solution of **1**$^{2+}$ (**a**) and **2**$^{2+}$ (**b**) immediately after a BE experiment in at -1.8V vs Fc^{+}/Fc DMF in the presence of 60 equivalents of p-TsOH showing the presence of the complex and supporting the molecular nature of the catalysis.

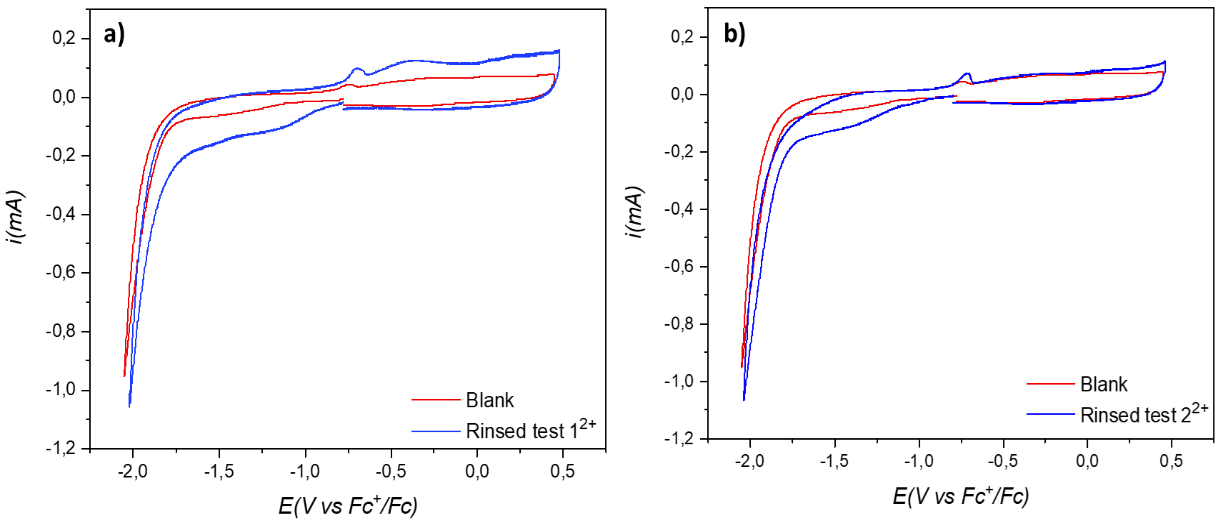

Figure S46- Cyclic Voltammograms of rinsed electrodes after bulk electrolysis with complex **1**$^{2+}$ (**a**) and **2**$^{2+}$ (**b**) at -1.8V vs Fc^{+}/Fc in a DMF solution with 60 equivalents of p-TsOH and 0.1 M $TBAPF_6$ as supporting electrolyte.

5.1- Determination of Faradic efficiency (FE), Turnover Number (TON), Turnover Frequency (TOF) and overpotential of the HER in DMF

The FE and TON are calculated using the following formulas:

$$\boldsymbol{FE}(\%) = \frac{\boldsymbol{n_{H_2\,detected}}}{\boldsymbol{n_{H_2\,theoretical}}} \times \mathbf{100} \qquad \textbf{(eq. S1)}$$

$$\boldsymbol{TON} = \frac{\boldsymbol{n_{H_2\,detected}}}{\boldsymbol{n_{cat}}} \qquad \textbf{(eq. S2)}$$

Where $n_{H2\,detected}$ is the number of mols molecular hydrogen detected by gas chromatography and $n_{H2\,theoretical}$ is the number of theoretical mols of H_2 calculated from the charge that pass through the electrode during bulk electrolysis experiments and n_{cat} is the mols of the catalysts used for molecular hydrogen production.

CP experiments were carried out in a gas-tight two-compartment cell with 6 mL headspace. Complexes were dissolved in 5ml dry acetonitrile containing 0.1M $TBAPF_6$ as supporting electrolyte placed in one compartment with glassy carbon rod (S=3.14cm^2 immersed in solution) as WE . reference electrode $Ag/AgNO_3$ (0.01M $Ag/AgNO_3$ in 0.1M $TBAPF_6$ in acetonitrile as supporting electrolyte) A Pt mesh was used as CE in the other compartment. Before any experiment, both compartments were degassed with N_2 for 30 minutes. The experiment was carried under stirring conditions.

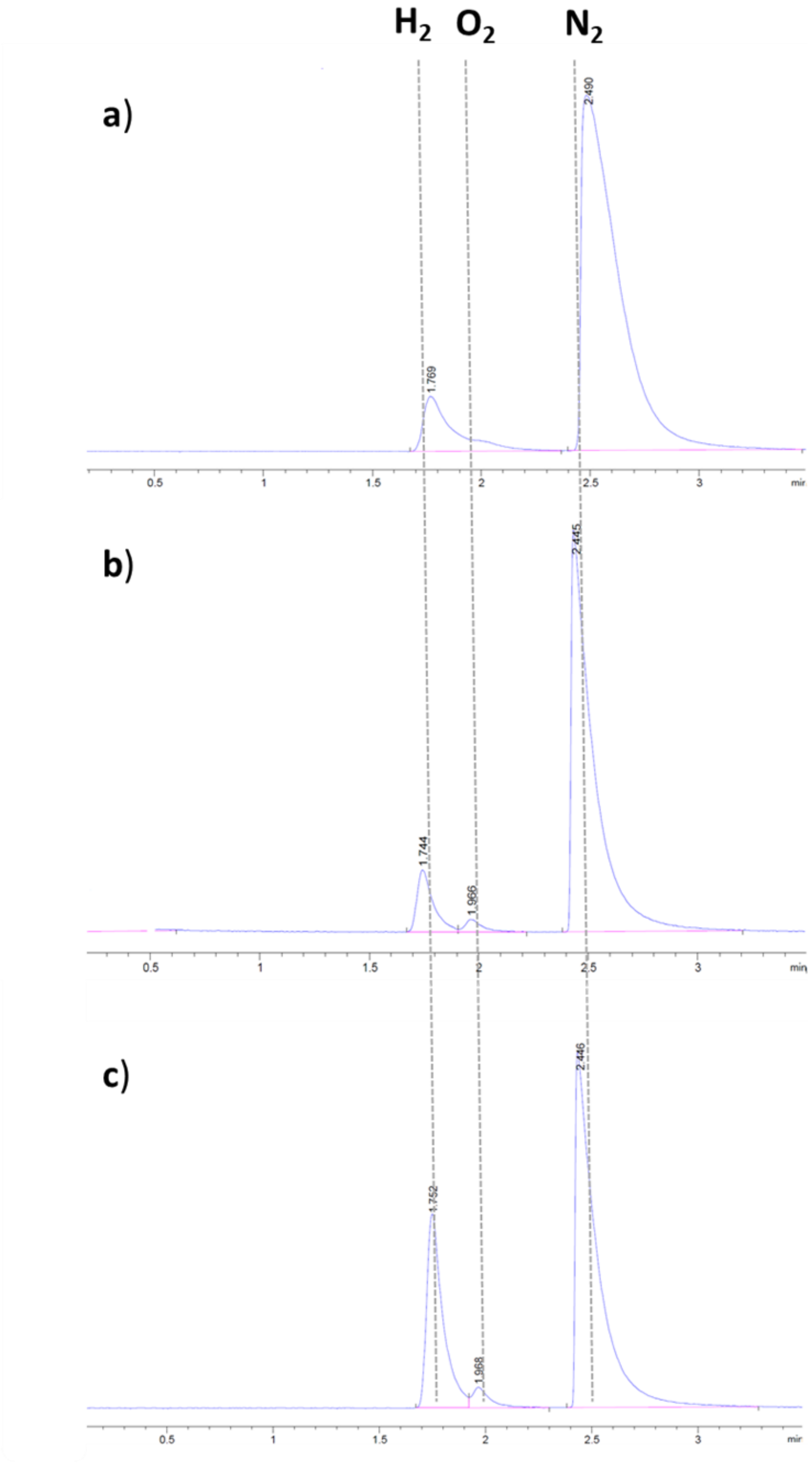

Figure S47- Chromatograms obtained by injection of 50µL of the head space of the cathodic compartment of a bulk electrolysis experiment at -1.8V vs Fc/Fc^+ after 900s, for solutions containing $\mathbf{1}^{2+}$(**a**), $\mathbf{2}^{2+}$(**c**) and blank (**b**). See Figures 9 and 10 of the main manuscript.

Calibration slope is required to correlate the peak area of the gas chromatography to the amount of H_2 contained in the headspace.

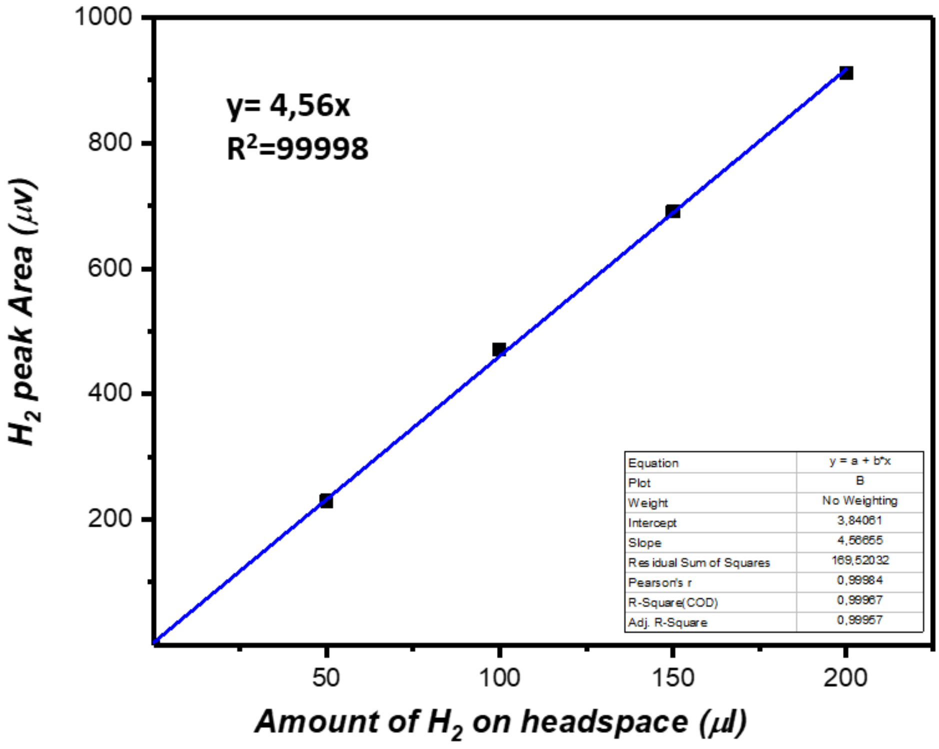

Figure S48- Calibration plot of the gas chromatography.

Table S1- Peak areas and volume of hydrogen calculated from the calibration plot in Figure S42.

Complex	*GC peak area (μV/s*	*V_{H2}(μl)*	*V_{H2}(μl)net[a]*	*Q(C)*	*$Q(C)_{net}$*
1^{2+}	338.3660	74.20	39	-1.952	0.936
2^{2+}	662.48163	145.28	110.08	-3.489	2.473
Blank	160.52	35.20	-	-1.016	-

[a]Volume of H_2 gas after subtraction of blank. [b]Charge after subtraction of blank.

Using the perfect gases equation, we can obtain the number of moles of hydrogen gas produced by the electrode and measured in the headspace.

$$PV = nRT \qquad \textbf{(eq. S3)}$$

Where P is the pressure of the headspace (1atm), R is the gas constant ($0.082\frac{atm \cdot L}{K \cdot mol}$), and T the temperature (298,15K)

To calculate the theoretical number of moles from the electrical charge transferred to the electrode, where n = 2:

$$2H^{+} + 2e^{-} \rightarrow H_2$$

$$n_{H2\ theoretical} = \frac{C}{F \cdot n} \qquad \textbf{(eq. S4)}$$

Table S1- Hydrogen evolution data for complex **1**$^{2+}$, **2**$^{2+}$ and blank (see Figures 9b and 10b in the manuscript).

Complex	$n_{(H2)\ detected}$	$n_{(H2),net}{}^{a}$	n_{Cat}	*TON*	$n_{(H2)theoretical}$	$n_{(H2)theoretical,net}{}^{a}$	*FE (%)*
1^{2+}	3.03×10^{-6}	1.59×10^{-6}	2.5×10^{-6}	0.64	1.01×10^{-5}	4.85×10^{-6}	33
2^{2+}	5.94×10^{-6}	4.5×10^{-6}	2.5×10^{-6}	1.80	1.808×10^{-5}	1.28×10^{-5}	35
Blank	1.44×10^{-6}	-	-	-	5.26×10^{-6}	-	27

[a]Mols of hydrogen after subtraction of the blank.

The overpotential of the reaction has been calculated according to the pK_a of *p*-TsOH in DMF[(1)] and equations S5 and S6,[(1,2)] giving a value of 0.78 V.

$$\boldsymbol{E_{H^+} = E^0_{H^+} - 0.059pK_a} \qquad \textbf{(eq. S5)}$$
$$\boldsymbol{Overpot = \left|E_{H^+} - E_{cat/2}\right|} \qquad \textbf{(eq. S6)}$$

Where $E_{H^+} = -0.77$ V *vs* $Fc^{+/0}$ for the H^+/H_2 couple in DMF, $pK_a = 2.6$ for *p*-TsOH in DMF and $E_{cat/2} \approx$ -1.70 V *vs* $Fc^{+/0}$ for both catalysts $\mathbf{1}^{2+}$ and $\mathbf{2}^{2+}$.[(1,2)]

(1) *Inorg. Chem.* **2006**, *45*, 9181-9184.
(2) *ACS Catal.* **2014**, 4, 630-633.

6- Additional Schemes

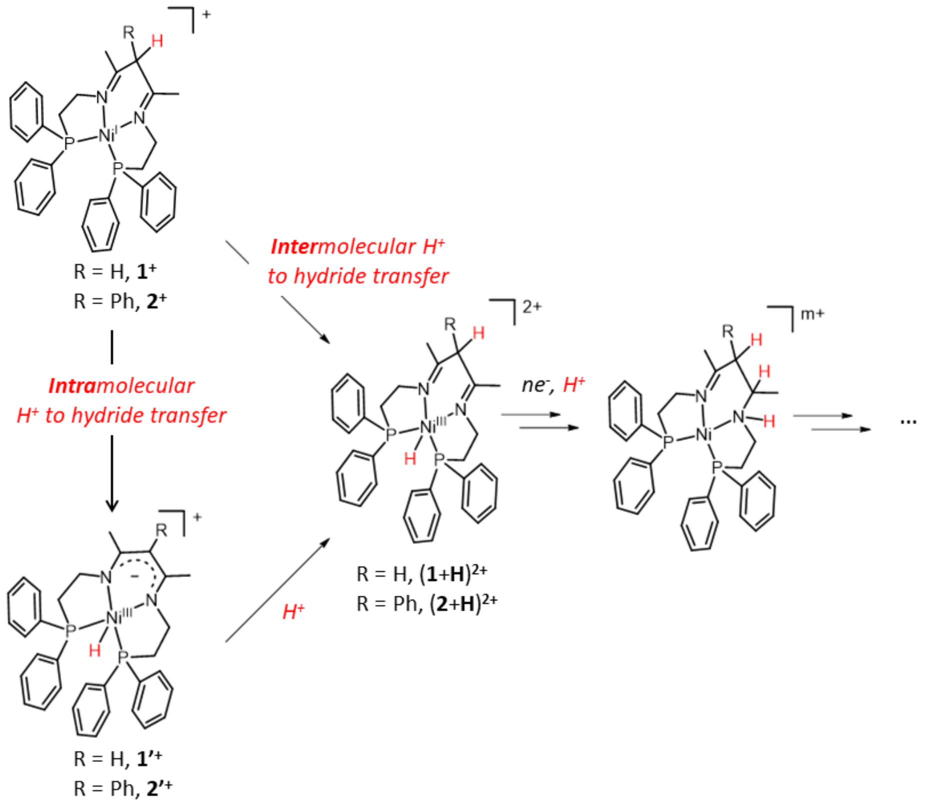

Scheme S1. Proposed reaction pathways after one-electron reduction of $\mathbf{1}^{2+}$ or $\mathbf{2}^{2+}$ in the presence of an acid source.

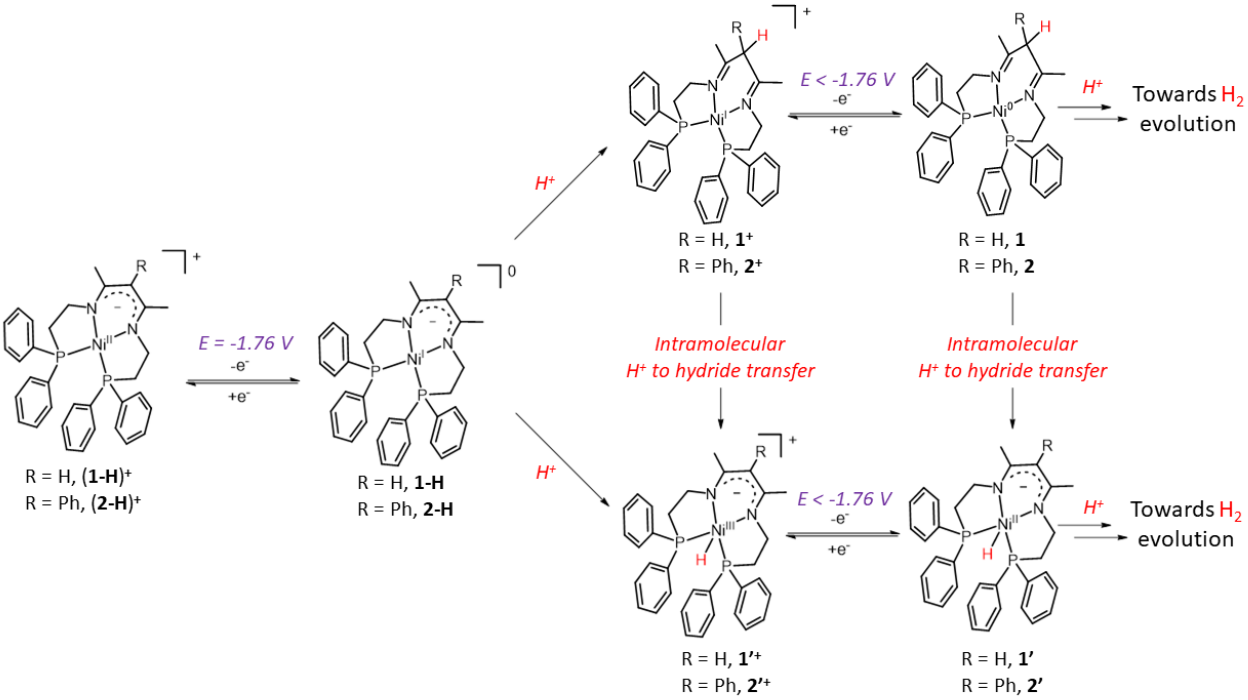

Scheme S2. Proposed routes towards the hydrogen evolution reaction from (**1-H**)$^{+}$ and (2-**H**)$^{+}$.
